# Supplementary material for: Association between sleep duration and hypertension in southwest China: a population-based cross-sectional study
Source: BMJ Open. 2022 Jun 27;12(6):e052193. doi: 10.1136/bmjopen-2021-052193 (PMC9237882; doi:10.1136/bmjopen-2021-052193)
Supplement: Author's manuscript [file bmjopen-2021-052193.draft_revisions.pdf]

BMJ Open is committed to open peer review. As part of this commitment we make the peer review history of every article we publish publicly available.

When an article is published we post the peer reviewers' comments and the authors' responses online. We also post the versions of the paper that were used during peer review. These are the versions that the peer review comments apply to.

The versions of the paper that follow are the versions that were submitted during the peer review process. They are not the versions of record or the final published versions. They should not be cited or distributed as the published version of this manuscript.

BMJ Open is an open access journal and the full, final, typeset and author-corrected version of record of the manuscript is available on our site with no access controls, subscription charges or pay-per-view fees (http://bmjopen.bmj.com).

If you have any questions on BMJ Open's open peer review process please email info.bmjopen@bmj.com

BMJ Open

The association between sleep duration and hypertension in southwest China: a population-based cross-sectional study

Journal:	*BMJ Open*
Manuscript ID	bmjopen-2021-052193
Article Type:	Original research
Date Submitted by the Author:	08-Apr-2021

Complete List of Authors:	Chang, Xiaoyu; Sichuan Center for Disease Control and Prevention, Department of Chronic and Non-communicable Disease Control and Prevention<br>Chen, Xiaofang; Chengdu Medical College, Department of Epidemiology and Statistics<br>Ji, John; Duke Kunshan University, Environmental Research Center; Duke University, Nicholas School of the Environment<br>Luo, Guojin; Pengzhou Center For Disease Control And Prevention<br>Chen, Xiaofang; Pengzhou Center For Disease Control And Prevention<br>Sun, Qiang; Pengzhou Center For Disease Control And Prevention<br>Zhang, Ningmei; Sichuan Center for Disease Control and Prevention<br>Guo, Yu; Chinese Academy of Medical Sciences<br>Pei, Pei; Chinese Academy of Medical Sciences<br>Li, Liming; Peking University, School of Public Health; Peking University, Peking University Center for Public Health and Epidemic Preparedness & Response<br>Chen, Zhengming; University of Oxford, Nuffield Department of Population Health<br>Wu, Xianping ; Sichuan Center for Disease Control and Prevention

Keywords:	Hypertension < CARDIOLOGY, SLEEP MEDICINE, EPIDEMIOLOGY, PUBLIC HEALTH

SCHOLARONE™
Manuscripts

I, the Submitting Author has the right to grant and does grant on behalf of all authors of the Work (as defined in the below author licence), an exclusive licence and/or a non-exclusive licence for contributions from authors who are: i) UK Crown employees; ii) where BMJ has agreed a CC-BY licence shall apply, and/or iii) in accordance with the terms applicable for US Federal Government officers or employees acting as part of their official duties; on a worldwide, perpetual, irrevocable, royalty-free basis to BMJ Publishing Group Ltd ("BMJ") its licensees and where the relevant Journal is co-owned by BMJ to the co-owners of the Journal, to publish the Work in this journal and any other BMJ products and to exploit all rights, as set out in our licence.

The Submitting Author accepts and understands that any supply made under these terms is made by BMJ to the Submitting Author unless you are acting as an employee on behalf of your employer or a postgraduate student of an affiliated institution which is paying any applicable article publishing charge ("APC") for Open Access articles. Where the Submitting Author wishes to make the Work available on an Open Access basis (and intends to pay the relevant APC), the terms of reuse of such Open Access shall be governed by a Creative Commons licence – details of these licences and which Creative Commons licence will apply to this Work are set out in our licence referred to above.

Other than as permitted in any relevant BMJ Author's Self Archiving Policies, I confirm this Work has not been accepted for publication elsewhere, is not being considered for publication elsewhere and does not duplicate material already published. I confirm all authors consent to publication of this Work and authorise the granting of this licence.

For peer review only

The association between sleep duration and hypertension in southwest China: a population-based cross-sectional study

Xiaoyu Chang,[1] Xiaofang Chen,[2] John S Ji,[3] Guojin Luo,[4] Xiaofang Chen,[4] Qiang Sun,[4] Ningmei Zhang,[1] Yu Guo,[5] Pei Pei,[5] Liming Li,[6] Zhengming Chen,[7] Xianping Wu[1]

Correspondence to Xianping Wu, wwwuxp@163.com

Author affiliations

[1]Department of Chronic and Non-communicable Disease Control and Prevention, Sichuan Center for Disease Control and Prevention, Chengdu, China

[2]Department of Epidemiology and Statistics, Chengdu Medical College, Chengdu, China

[3]Environmental Research Center, Duke Kunshan University, Kunshan, China; Nicholas School of the Environment, Duke University, Durham, NC, USA.

[4]Pengzhou Center for Disease Control and Prevention, Pengzhou, China

[5]Chinese Academy of Medical Sciences, Beijing, China

[6]Department of Epidemiology& Biostatistics, School of Public Health, Peking University Health Science Center , Beijing, China; Peking University Center for Public Health and Epidemic Preparedness & Response, Beijing, China

[7]Clinical Trial Service Unit and Epidemiological Studies Unit (CTSU), Nuffield Department of Population Health, University of Oxford, Oxford, UK

Word count: 3983

For peer review only

For peer review only - http://bmjopen.bmj.com/site/about/guidelines.xhtml

ABSTRACT

Objective Hypertension is a major risk factor and cause of many non-communicable diseases in China. While there have been studies on various diet and lifestyle risk factors, we do not know whether sleep duration has an association to blood pressure in southwest China. This predictor is useful in low-resource rural settings. We examined the association between sleep duration and hypertension in southwest China.

Design Population-based cross-sectional study

Setting This study was part of the baseline survey of a large ongoing prospective cohort study, the China Kadoorie Biobank. Participants were enrolled in 15 townships of Pengzhou city in Sichuan province during 2004-2008.

Participants 55 687 participants aged 30 to 79 years were included. Sleep duration was assessed by a self-reported questionnaire.

Main outcome measures Hypertension was defined as systolic blood pressure ≥ 140 mm Hg and/or diastolic blood pressure ≥ 90 mm Hg, or prior physician-diagnosed hypertension in hospitals at the township (community) level.

Results The prevalence of hypertension was 25.17%. The percentages of subjects with sleep durations of <6, 6, 7, 8 and ≥ 9 hours were 17.20%, 16.14%, 20.04%, 31.95% and 14.68%, respectively. In multivariable adjusted analyses, the increased odds ratios (95% CIs) of having hypertension was across those who reported ≥ 9 hours of sleep (men: 1.16, 1.04 -1.30; women: 1.19, 1.08 -1.32; general population: 1.17, 1.08 -1.26). Covariate-adjusted analyses stratified by health-related factors observed that long sleep were associated with elevated hypertension prevalence for those who were ≥ 45 years, regular tea consumers, never or ex- or regular alcohol consumers, never or regular smoker, non-regular fresh fruit consumers and insomnia subgroup.

Conclusion Long sleep duration were significantly associated with hypertension in adults of southwest China, independent of potential confounders. However, this association was not obvious between short sleep duration and hypertension.

Strengths and limitations of this study

For peer review only - http://bmjopen.bmj.com/site/about/guidelines.xhtml

This study examined the association between sleep duration and hypertension in southwest China, and this predictor was useful in low-resource rural settings.

A large sample of rural population was obtained, which could represent most of the rural places in southwest China.

A broad range of covariates were controlled in the analysis, including age, sex, education, occupation, marital status, income, tea, smoking, alcohol, MET, sedentary leisure time, fresh fruit consumption, insomnia, sleep snoring, BMI, WC, DM, stroke and CHD.

The sleep duration was self-reported without more detailed and objective measures of sleep, which was less accurate than actigraphy and polysomnography.

For peer review only

INTRODUCTION

Hypertension is a condition in which the blood vessels have persistently raised pressure.[1] It is the leading risk factor for global cardiovascular disease burden and mortality.[2] In China, hypertension is also a serious public health problem. The China hypertension survey showed that the prevalence of hypertension and pre-hypertension were 23.2% and 41.3% among Chinese adults, which means that there is one hypertensive patient per four people.[3] High systolic blood pressure was the leading risk factors contributing to deaths and DALYs.[4] In addition, hypertension is a growing problem not only in urban but also in rural China. The prevalence of hypertension has sharply increased in rural China,[5] which is similar in rural and urban settings (23.4% versus 23.1%).[3] Disease burden of hypertension is increasing globally, especially as places of poverty become more prosperous.[2]

Previous studies about influencing factors of hypertension mainly focus on diet (high dietary sodium intake), exercise and other lifestyle factors,[6 7] while some studies in recent years documented the relationship between sleep and hypertension. Sleep status influence blood pressure by altering autonomic nervous system function and other physiologic events. Unhealthy sleep status such as sleep apnea, insomnia, long and short sleep duration can increase risk of hypertension. Even though a variety of studies examined sleep duration to be associated with hypertension, the results were not completely consistent. Some studies showed that short sleep duration increased the risk of hypertension,[8-14] others showed that long sleep duration was related to hypertension or the association were J- or U-shaped curves.[15-18] However, most of the study populations were from western countries. A small number of studies were conducted in Asian countries,[9 10 13 19] and there was limited information about the association between sleep duration and hypertension in southwest China. Therefore, the aim of this study is to analyze the baseline data from a large prospective cohort study of CKB to explore the association of sleep duration with hypertension among more than 50 000 adults in Southwest China.

For peer review only - http://bmjopen.bmj.com/site/about/guidelines.xhtml

SUBJECTS AND METHODS

Study population

This study utilizes a large, ongoing prospective cohort study, the China Kadoorie Biobank (CKB). The baseline survey of CKB was conducted in five urban and five rural areas across China from 2004 to 2008. Our data was derived from the baseline survey of CKB in Sichuan province. A total of 55 687 residents aged 30 to 79 years in 15 townships of Pengzhou city in Sichuan province had completed baseline information collection, including a questionnaire, anthropometric measurements, blood sample collection, and a written informed consent. More details about the study design are available in previous publications.[20-22]

Data collection

The baseline survey took place in temporary study clinics, specially set up near participants' residential areas. Information on sociodemographic characteristics (age, sex, education level, occupation, annual household income), behavioural risk factors for chronic diseases (diet, tea consumption, smoking status, alcohol intake, physical activity, sedentary leisure time, sleep), and medical history were collected through an interviewer-administered computerized questionnaire by face-to-face interview with trained interviewers according to standard protocols and procedures. A range of physical measurements were also undertaken, including height, weight, waist circumference and so on.

Outcome measurement ascertainment

Sleep duration was assessed by a self-reported questionnaire with the following question: 'How many hours do you typically sleep per day (including daytime naps)?'. To maintain consistency with previous literatures,[10 17 18 23] sleep duration was categorized as five groups of <6, 6, 7 (which regarded as the reference group for our analysis), 8 and ≥ 9 hours.

As described in the previous study,[22] blood pressure was measured twice using a UA-779 digital monitor recommended by the British Hypertension Society (A & D Instruments Ltd., Abingdon, UK). If the systolic blood pressure (SBP) difference between the first two

For peer review only

For peer review only - http://bmjopen.bmj.com/site/about/guidelines.xhtml

measurements was more than 10 mm Hg, a third measurement was obtained and the last two measurements were recorded. The mean of two blood pressure measurements was used for this study. Hypertension was defined as systolic blood pressure (SBP) ≥ 140 mm Hg and/or diastolic blood pressure (DBP) ≥ 90 mm Hg, or prior physician-diagnosed hypertension in hospitals at the township (community) level.

Several variables were used both as covariates in adjusted models and as variables for stratification. Sociodemographic characteristics included age at baseline survey (30-79 years old), sex (male or female), education level (no formal school, primary school, middle school, high school and above), occupation (famer or others), marital status (married or never married/widowed/separated/divorced), annual household income (< 5 000, 5 000~9 999, ≥ 10 000, yuan). Behavioural risk factors included tea consumption (never or almost never, only occasionally, usually at least once a week), smoking status (never, ex-regular, occasionally, daily or almost every day), alcohol intake (never regular, ex-regular, occasionally or seasonally, usually at least once a week), fresh fruit consumption (daily, 4-6 days per week, 1-3 days per week , monthly, never/rarely), physical activity, sedentary leisure time (hours/week), insomnia symptoms (yes or no), sleep snoring (never, occasionally, usually) , waist circumference (WC) and body mass index (BMI). Medical history included diabetes (DM), stroke and coronary heart disease (CHD) and so on. BMI was calculated as weight in kilograms (kg) / height in metres $(m)^2$. According to the Guidelines for Prevention and Control of Overweight and Obesity in Adults in China,[24] BMI was divided into four groups: underweight (<18.5 kg/m^2), normal weight (18.5-24 kg/m^2), overweight (24-28 kg/m^2) and obesity (≥28 kg/m^2).

Information about type, frequency, and duration of occupational, commuting-related, household and active recreational (leisure-time) physical activities were used to calculate total physical activity in metabolic equivalents (MET, hours/day).[25 26] Sedentary leisure time (hours/week) was assessed by the question of "About how many hours per week did you watch TV or read?". Insomnia symptoms were determined by three questions: Taking >30 minutes to fall asleep after going to bed or waking up in the middle of the night; Waking up early and not being able to go back to sleep; Having difficulty staying alert while at work, eating or meeting

For peer review only - http://bmjopen.bmj.com/site/about/guidelines.xhtml

people during daytime. Those reporting "Yes" for one or more of the three questions were classified as having insomnia symptoms.[27] Participants consumed fresh fruit more than 3 days/week were defined as regular consumers, otherwise, those were never or occasionally consumers.[25]

Statistical Analysis

Continuous variables were expressed as $\bar{\chi}\pm s$ and categorical variables were expressed as composition ratio or rate. The analysis of variance was used for continuous variables and chi-square test for categorical variables to determine the association of the characteristics of the study participants by different sleep duration. Multivariable logistic regression models were used to calculate the odds ratios (ORs) and 95% confidence intervals (CIs) for hypertension between sleep duration subgroups adjusted for potential confounders. In addition, we performed subgroup analysis stratified by health-related factors, such as age, sex, tea consumption, smoking status, alcohol intake, insomnia. A 2-tailed P-value of < 0.05 was considered statistically significant. All data processing and statistical analyses were performed using Statistical Analysis Software, version 9.4 (SAS Institute, Cary, NC) and R version 4.0.3 (R Foundation for Statistical Computing, Vienna, Austria).

Patient and public involvement

No patients were involved in the present study, the interpretation of study results or write-up of the manuscript. The results were presented to study participants by the website of the CKB (http://www.ckbiobank.org/site/) and newsletters.

RESULTS

Baseline characteristics of study population

The baseline characteristics of study population were showed in **Table 1**. A total of 55 687 adults completed baseline information, including 34 372 women and 21 315 men. The mean (±SD) age of study population was 51.03±10.54 years (range: 30–79 years). The mean (±

For peer review only - http://bmjopen.bmj.com/site/about/guidelines.xhtml

SD) sleep duration was 7.05±1.68 hours, which were 7.07±1.61 hours in men and 7.04±1.72 hours in women. The overall means (±SD) of SBP and DBP were 129.24±19.01 mm Hg and 77.23 ± 10.23 mm Hg, respectively. The percentages of the subjects with sleep durations of <6, 6, 7, 8 and ≥ 9 hours were 17.20%, 16.14%, 20.04%, 31.95% and 14.68%, respectively.

Individuals with less hours of sleep per day were more likely to be female, eldly, low education level, famer, single, low annual household income, never or almost never tea consumers, low frequency of fresh fruit consumption, low BMI. Short sleep duration was more likely to be DM, CHD, stroke or hypertensive subjects. With the increase of age, the proportion of subjects who slept for equal to or more than 8 hours decreased, while the proportion of subjects who slept for equal to or less than 6 hours increased. With the increase of education level, the proportion of subjects with less than 6 hours of sleep decreased. With the increase of annual household income and tea consumption, the proportion of subjects who slept for less than 6 hours decreased. With the increase of BMI and fresh fruit consumption, the proportion of subjects who slept for less than 6 hours decreased, while the proportion of subjects who slept for equal to or more than 9 hours increased.

Table 1 Baseline characteristics of study population in different sleep duration subgroups

	**Sleep duration(hours)**							
**Variables**	**<6**	**6**	**7**	**8**	**≥9**	**Overall**	**χ^2/F**	**P**
Number of subjects	9576(17.2)	8988(16.14)	11159(20.04)	17790(31.95)	8174(14.68)	55687(100)		
Gender[a]								
Female	6271(18.24)	5273(15.34)	6594(19.18)	10898(31.71)	5336(15.52)	34372(100)	171.051	<.0001
Male	3305(15.51)	3715(17.43)	4565(21.42)	6892(32.33)	2838(13.31)	21315(100)		
Age(years)[a]								
35~39	604(6.21)	998(10.27)	1802(18.54)	4142(42.60)	2176(22.38)	9722(100)	4406.276	<.0001
40~49	1651(11.01)	2087(13.91)	3170(21.13)	5523(36.82)	2571(17.14)	15002(100)		
50~59	3560(19.69)	3278(18.13)	3815(21.11)	5262(29.11)	2161(11.96)	18076(100)		
60~69	2775(27.41)	2050(20.25)	1962(19.38)	2340(23.12)	996(9.84)	10123(100)		
70~79	986(35.67)	575(20.80)	410(14.83)	523(18.92)	270(9.77)	2764(100)		
Education level[a]								
No formal school	2610(30.38)	1555(18.10)	1452(16.90)	1942(22.60)	1033(12.02)	8592(100)	2440.546	<.0001
Primary school	5146(18.53)	4759(17.14)	5539(19.95)	8401(30.26)	3920(14.12)	27765(100)		
Middle school	1444(9.70)	2040(13.70)	3059(20.55)	5722(38.44)	2621(17.61)	14886(100)		
High school and above	376(8.46)	634(14.27)	1109(24.95)	1725(38.82)	600(13.50)	4444(100)		
Occupation[a]								

For peer review only - http://bmjopen.bmj.com/site/about/guidelines.xhtml

Famer	8674(17.73)	7862(16.07)	9629(19.68)	15531(31.74)	7236(14.79)	48932(100)	102.183	<.0001
Others	902(13.35)	1126(16.67)	1530(22.65)	2259(33.44)	938(13.89)	6755(100)		
Marital status[a]								
Married	8040(15.91)	7990(15.81)	10236(20.26)	16610(32.87)	7650(15.14)	50526(100)	797.729	<.0001
Never Married/Widowed/Separated / Divorced	1536(29.76)	998(19.34)	923(17.88)	1180(22.86)	524(10.15)	5161(100)		
Annual household income (yuan) [a]								
<5,000	3730(24.61)	2454(16.19)	2761(18.22)	3996(26.37)	2215(14.61)	15156(100)	991.831	<.0001
5,000~9,999	3145(15.98)	3168(16.09)	3867(19.65)	6532(33.18)	2972(15.10)	19684(100)		
≥10,000	2701(12.96)	3366(16.15)	4531(21.73)	7262(34.83)	2987(14.33)	20847(100)		
Tea consumption[a]								
Never or almost never	2311(23.51)	1534(15.61)	1768(17.99)	2692(27.39)	1525(15.51)	9830(100)	581.800	<.0001
Only occasionally	4822(17.76)	4488(16.53)	5385(19.83)	8695(32.02)	3764(13.86)	27154(100)		
Usually at least once a week	2443(13.06)	2966(15.86)	4006(21.42)	6403(34.24)	2885(15.43)	18703(100)		
Smoking status[a]								
Never	4896(16.38)	4418(14.78)	5771(19.30)	9998(33.44)	4814(16.10)	29897(100)	350.690	<.0001
Ex-regular smoker	835(22.14)	675(17.90)	786(20.84)	996(26.41)	479(12.70)	3771(100)		
Occasional smoker	892(20.14)	827(18.67)	892(20.14)	1272(28.71)	547(12.35)	4430(100)		
Daily or almost every day	2953(16.79)	3068(17.44)	3710(21.09)	5524(31.41)	2334(13.27)	17589(100)		
Alcohol intake[a]								
Never regular drinker	3591(18.80)	2926(15.32)	3575(18.72)	6031(31.57)	2979(15.60)	19102(100)	204.099	<.0001
Ex-regular drinker	463(22.43)	371(17.97)	439(21.27)	512(24.81)	279(13.52)	2064(100)		
Occasional or seasonal drinker	3548(16.40)	3456(15.97)	4439(20.51)	7118(32.89)	3078(14.22)	21639(100)		
Usually at least once a week	1974(15.32)	2235(17.35)	2706(21.01)	4129(32.05)	1838(14.27)	12882(100)		
Fresh fruit consumption[a]								
Daily	736(11.53)	863(13.52)	1376(21.56)	2218(34.76)	1188(18.62)	6381(100)	1185.806	<.0001
4-6 days per week	716(12.75)	814(14.50)	1134(20.20)	1931(34.39)	1020(18.17)	5615(100)		
1-3 days per week	3688(15.35)	3720(15.49)	4654(19.38)	8347(34.75)	3611(15.03)	24020(100)		
Monthly	3401(21.93)	2890(18.63)	3210(20.69)	4262(27.48)	1748(11.27)	15511(100)		
Never/rarely	1035(24.88)	701(16.85)	785(18.87)	1032(24.81)	607(14.59)	4160(100)		
BMI(Kg/m2) [a]								
<18.5	625(25.31)	436(17.66)	462(18.71)	642(26.00)	304(12.31)	2469(100)	161.222	<.0001
18.5~23.9	5523(17.33)	5094(15.99)	6399(20.08)	10209(32.04)	4642(14.57)	31867(100)		
24~27.9	2700(16.12)	2718(16.23)	3393(20.26)	5416(32.34)	2519(15.04)	16746(100)		
≥28	728(15.81)	740(16.07)	905(19.65)	1523(33.07)	709(15.40)	4605(100)		
DM[a]								
No	9050(16.89)	8606(16.06)	10751(20.07)	17257(32.21)	7908(14.76)	53572(100)	118.858	<.0001
Yes	526(24.87)	382(18.06)	408(19.29)	533(25.20)	266(12.58)	2115(100)		
CHD[a]								

For peer review only

No	9501(17.15)	8929(16.11)	11101(20.03)	17734(32.00)	8147(14.70)	55412(100)	37.361	<.0001
Yes	75(27.27)	59(21.45)	58(21.09)	56(20.36)	27(9.82)	275(100)		
Stroke[a]								
No	9509(17.17)	8941(16.14)	11115(20.06)	17719(31.99)	8112(14.64)	55396(100)	23.279	0.0001
Yes	67(23.02)	47(16.15)	44(15.12)	71(24.40)	62(21.31)	291(100)		
Hypertension[a]								
No	6530(15.67)	6527(15.66)	8473(20.33)	13865(33.27)	6278(15.06)	41673(100)	362.709	<.0001
Yes	3046(21.74)	2461(17.56)	2686(19.17)	3925(28.01)	1896(13.53)	14014(100)		
age(y)[b]	56.30±9.94	53.37±10.19	50.8±10.07	48.60±10.09	47.9±10.31	51.03±10.54	1224.030	<.0001
MET(h/day)[b]	21.08±11.47	21.98±11.89	22.67±12.03	22.72±11.65	21.12±11.70	22.07±11.76	51.170	<.0001
WC(cm)[b]	77.45±9.27	78.27±9.11	78.16±8.98	78.07±8.96	77.99±9.05	78.00±9.06	11.800	<.0001
BMI(Kg/m2)[b]	23.00±3.29	23.29±3.21	23.32±3.18	23.38±3.15	23.39±3.19	23.29±3.20	25.120	<.0001
Sedentary leisure time(h/week)[b]	25.15±10.22	25.83±10.45	25.98±10.50	26.85±10.53	27.28±11.39	26.28±10.61	64.490	<.0001
SBP(mmHg)[b]	132.28±20.24	130.23±19.23	128.61±18.73	127.81±18.18	128.52±18.96	129.24±19.01	99.140	<.0001
DBP(mmHg)[b]	77.65±10.51	77.34±10.34	76.96±10.24	77.02±9.97	77.45±10.28	77.23±10.23	8.940	<.0001

a. Data are expressed as n (%). The percentages in table are row percent. χ^2 tests were conducted to test for differences in percentage for categorical variables.

b. Data are expressed as mean±standard deviation . One-way anova test were conducted to test for differences in means for categorical variables.

Abbreviations: BMI, body mass index; DM, diabetes mellitus; CHD, Coronary Heart Disease; WC, waist circumference; MET, Metabolic Equivalent; SBP, Systolic Blood Pressure; DBP, diastolic blood pressure.

The relationship between sleep duration and hypertension

Results of the logistic regression analyses examined relationship between sleep duration and hypertension were showed in **Table 2**. The prevalence of hypertension was 25.17% (14 014/55 687). A quarter of participants were considered hypertensive, slightly more in men (28.67%, 6111/21 315) than in women (22.99%, 7903/34 372). Compared to 7-hours sleepers (reference group), the unadjusted analyses showed that short sleep duration increased risk of hypertension, with short sleepers (< 6 hours and 6 hours) having higher odds of hypertension (OR = 1.47 and 1.19, respectively; P < 0.05). After adjustment for age, sex, education level, occupation, marital

For peer review only

For peer review only - http://bmjopen.bmj.com/site/about/guidelines.xhtml

status, annual household income, tea consumption, smoking status, alcohol intake, MET, sedentary leisure time, fresh fruit consumption, insomnia, sleep snoring, BMI, WC, DM, stroke and CHD, the increased odds of having hypertension was across those who reported ≥ 9 hours of sleep (OR = 1.17, 95% CI : 1.08-1.26; $P < 0.05$). However, short sleep duration was no longer associated with risk of hypertension.

Men and women were similar results after adjustment for potential confounding factors. Long sleep duration was associated with elevated risk of hypertension, with long sleepers (≥9 hours) having higher odds of hypertension in men (OR = 1.16, 95% CI : 1.04-1.30; $P < 0.05$) and women (OR =1.19, 95% CI : 1.08-1.32; $P < 0.05$). The observed association between sleep duration and hypertension was slightly attenuated among men compared with women.

For peer review only - http://bmjopen.bmj.com/site/about/guidelines.xhtml

Table 2 Multivariable adjusted odds ratio by logistic regression analyses of sleep duration associated with hypertension

	Sleep duration(hours)				
**population**	**<6**	**6**	**7**	**8**	**≥9**
Total[a] (n/N, Pre%)	3046/9576(31.81)	2461/8988(27.38)	2686/11159(24.07)	3925/17790(22.06)	1896/8174(23.20)
model1	1.47(1.38-1.56) *	1.19(1.12-1.27) *	1	0.89(0.84-0.94)	0.95(0.89-1.02)
model2	1.02(0.96-1.09)	0.99(0.93-1.06)	1	1.05(0.99-1.11)	1.19(1.10-1.27) *
model3	1.02(0.95-1.09)	0.99(0.93-1.06)	1	1.05(0.99-1.11)	1.18(1.10-1.27) *
model4	0.98(0.91-1.05)	0.98(0.92-1.05)	1	1.05(0.99-1.12)	1.16(1.08-1.24) *
model5	0.96(0.89-1.04)	0.96(0.90-1.03)	1	1.06(0.99-1.12)	1.17(1.08-1.26) *
Male[b] (n/N, Pre%)	1113/3305(33.68)	1108/3715(29.83)	1263/4565(27.67)	1803/6892(26.16)	824/2838(29.03)
model1	1.33(1.21-1.46) *	1.11(1.01-1.22) *	1	0.93(0.85-1.01)	1.07(0.96-1.19)
model2	0.97(0.87-1.07)	0.97(0.88-1.07)	1	1.03(0.94-1.13)	1.19(1.07-1.33) *
model3	0.96(0.86-1.06)	0.97(0.87-1.07)	1	1.03(0.95-1.13)	1.19(1.07-1.33) *
model4	0.92(0.82-1.03)	0.95(0.86-1.05)	1	1.04(0.95-1.14)	1.17(1.05-1.31) *
model5	0.89(0.79-1.00)	0.93(0.84-1.03)	1	1.04(0.95-1.14)	1.16(1.04-1.30) *
Female[b] (n/N, Pre%)	1933/6271(30.82)	1353/5273(25.66)	1423/6594(21.58)	2122/10898(19.47)	1072/5336(20.09)
model1	1.62(1.50-1.75) *	1.25(1.15-1.37) *	1	0.88(0.82-0.95)	0.91(0.84-1.00)
model2	1.05(0.97-1.15)	1.01(0.92-1.11)	1	1.07(0.98-1.15)	1.20(1.09-1.32) *
model3	1.05(0.96-1.14)	1.01(0.92-1.11)	1	1.07(0.99-1.16)	1.20(1.09-1.32) *
model4	1.01(0.91-1.11)	1.00(0.91-1.09)	1	1.07(0.98-1.16)	1.17(1.06-1.29) *
model5	1.00(0.90-1.11)	0.98(0.89-1.07)	1	1.07(0.99-1.17)	1.19(1.08-1.32) *

Data were analyzed as odds ratios (95% confidence intervals) for the "presence of hypertension" relative to "without hypertension" by logistic regression models using the 7 hours sleep duration as the reference group.

*$p < 0.05$.

a. Model 1 was unadjusted; Model 2 was adjusted for age and sex; Model 3 was adjusted for model 2 + education level, occupation, marital status, annual household income; Model 4 was adjusted for model 3 + tea consumption, smoke status, alcohol intake, MET, sedentary leisure time, fresh fruit consumption, insomnia, sleep snoring; Model 5 was adjusted for model 4 + BMI, WC, DM, stroke and CHD.

b. Model 1 was unadjusted; Model 2 was adjusted for age; Model 3 was adjusted for model 2 + education level, occupation, marital status, annual household income; Model 4 was adjusted for model 3 + tea consumption, smoke status, alcohol intake, MET, sedentary leisure time, fresh fruit consumption, insomnia, sleep snoring; Model 5 was adjusted for model 4 + BMI, WC, DM, stroke and CHD.

The relationship between sleep duration and hypertension Stratification by health-related factors

Results of covariate-adjusted analyses stratified by health-related factors examining

For peer review only - http://bmjopen.bmj.com/site/about/guidelines.xhtml

relationships between sleep duration and hypertension are displayed in **Figure 1**. Long sleep was associated with elevated hypertension prevalence for 45-59 and 60-79 years old subgroup, though the relationship was nominally stronger among 45-59 individuals. However, this association was not found among 30-44 years old subgroup. Among those usually at least once a week tea consumers, long sleep was associated with hypertension, and the relationship was not present across never/almost never or only occasionally tea consumers. Among those who were never regular alcohol drinker, ex-regular or usually at least once a week alcohol drinker, long sleep duration was associated with elevated hypertension risk. However, among occasional or seasonal alcohol drinker, the relationship with long sleep was not present. Among different smoke status groups, long sleep was associated with high risk of hypertension among never smoker and daily or almost every day smoker, and this association was not observed among ex-regular and occasional smoker. Long sleep was associated with elevated hypertension prevalence for non-regular fresh fruit consumers, while not among regular fresh fruit consumers. Long sleep was associated with elevated hypertension prevalence for insomnia subgroup, while not among non-insomnia subgroup.

For peer review only

For peer review only - http://bmjopen.bmj.com/site/about/guidelines.xhtml

DISCUSSION

This study examined the relationship between sleep duration and hypertension among more than 50 000 rural adults in southwest China. We found that long sleep duration equal to or more than 9 hours per day was a risk factor for hypertension among men, women and the general population, which was consistent with several previous studies in different countries.[16-18 28] For example, the first study assessed the association between study duration and hypertension conducted by Gottlieb et. al[17] reported that compared to subjects sleeping 7 to less than 8 hours per night, the adjusted ORs (95% CIs) for hypertension of those sleeping 8 hours and 9 or more hours were 1.19 (1.04-1.37) and 1.30 (1.04-1.62), respectively. Long sleep durations of equal to or more than 8 hours per night were associated with a significantly increased risk of hypertension in participants aged 40–100 years. The Nurse' health study[28] found long sleep durations (≥9 hours) was associated with hypertension prevalence in cross-sectional study. A systematic review and meta-analysis conducted by Guo et. al[29] found that long sleep duration were associated with risk of hypertension (OR = 1.11, 95% CI: 1.04-1.18). Wang et. al[30] conducted a meta-analysis, which indicated that the OR (95% CI) for long sleep duration and the risk of prevalent hypertension was 1.11 (1.05-1.17).

However, numerous studies showed that compared with normal sleep duration, short sleep duration was more likely to increase risk of hypertension than long sleep duration.[9-12 31 32] A cross-sectional study conducted among rural population in China showed that short sleep duration was associated with hypertension in women.[32] Another cross-sectional study in China including 43 655 participants found that short sleep duration as less than 6 hours was associated with risk of hypertension in both genders.[10] Results from the Kaiyuan study showed that short sleep duration (⩽ 5 hours per night) was associated with an increased risk for incidence of hypertension during the 3.98 years follow-up period among women and those aged <60 years.[9] A prospective study conducted by Gangwisch et. al reported that short sleep duration (⩽ 5 hours per night) were associated with a significantly increased risk of hypertension (HR = 2.10; 95% CI, 1.58 to 2.79) in subjects between 32 to 59 years old.[11]

For peer review only - http://bmjopen.bmj.com/site/about/guidelines.xhtml

Inconsistent with the 'J' or 'U' pattern in other studies,[15-17 33] the association between short sleep duration and hypertension was not found in this study. There are plausible explanations for the different results of these studies. First, different definition of sleep duration groups and adjustment of different possible influencing factors would lead to differences in statistical analysis. Second, the discrepancy may be due to the difference of study population. Different demographic factors and behavioral habits also would lead to the differences of the relationship between sleep duration and hypertension. Previous studies indicated that both insomnia[34] and snoring[35] could elevated risk of hypertension, and our study adjusted these factors for multivariate analysis.

The association between sleep duration and hypertension could reflect a variety of mechanisms. Several studies indicated that potential pathophysiological mechanisms supporting the association between short sleep duration and hypertension in humans may be sleep deprivation. Sleep deprivation has been reported as a cause of over-activity of the sympathetic nervous system, which may elevate blood pressure.[11 36] In addition, sleep deprivation could disrupt circadian rhythmicity, contribute to over-activity of the renin-angiotensin aldosterone system, pro-inflammatory responses, endothelial dysfunction, and renal impairment. Moreover, sleep deprivation could increase appetite and suppress renal salt fluid excretion, leading to elevated caloric consumption, excessive salt intake and weight gain, which are proved to be risk factors of hypertension.[11 37] However, the mechanisms of long sleep duration with hypertension are even not known. Possible explanations may be as follows. First, some other risk factors might impact this association. Long sleepers may have less physical activities,[38] and be related to sleep disorders (sleep apnea, etc.) or poor sleep quality, which could lead to adverse healthy outcomes.[16] In previous studies, long sleep was associated with increased all-cause mortality, high risk of diabetes and other unhealthy outcomes.[38-40] Second, sleep duration may reflect sleep need, which is different in individuals due to genetic, behavioral, and environmental factors.[17] Individuals with increased sleep needs may have a reduced physiologic reserve, reducing their ability to survive serious illness.[38] Third, sleep habits may be a marker of health status rather than a cause for disease, so it is likely that long

For peer review only - http://bmjopen.bmj.com/site/about/guidelines.xhtml

sleep duration is an epiphenomenon instead of an etiological factor for hypertension.[18 41]

The relationship between sleep duration and hypertension may varied by health-related factors. This study made stratified analyses of sleep duration and hypertension stratified by age, tea consumption, alcohol intake, smoking status, fresh fruit consumption and insomnia. Long sleep were associated with elevated hypertension prevalence among participants aged 45 to 79 years old. However, this association was not found among those aged 30-44 years old. This finding was different from several previous studies. For example, a Korean study found that short sleep durations were associated with hypertension prevalence only in those aged less than 60 years.[42] A Spanish study demonstrated that sleep duration was not associated with hypertension in older adults.[43] A cross sectional study conducted in northeast China observed an association between short sleep duration and an increased risk of hypertension in young adults aged 18 to 44 years, and the relationship was not found for participants aged 45 to 79 years.[19] Changes in sleep quality and quantity in different stage of life may be related to this age-dependent association.[44] In addition, healthy sleep was influenced by a lot of factors. Besides sleep duration, sleep disorders such as insomnia,[45 46] obstructive sleep apnea[47] and other sleep quality problems[48-50] have also been shown to be risk factors for hypertension. Moreover, the number of younger subjects was larger than that of older subjects, so the lack of an association between long sleep duration and hypertension in the younger subjects could be attribute to a lack of statistical power.

In this study, long sleep were associated with elevated hypertension prevalence for non-regular fresh fruit consumers, while not among regular fresh fruit consumers. Fresh fruit intake could reduce the risk and mortality of cardiovascular disease. Result from the previous prospective analyses of CKB showed that compared with participants who never or rarely consumed fresh fruit, those who ate fresh fruit daily had lower systolic blood pressure (by 4.0 mm Hg, P<0.001). The adjusted hazard ratios for daily consumers were 0.60 (95% CI, 0.54 to 0.67) for cardiovascular death, and 0.66 (95% CI, 0.58 to 0.75), 0.75 (95% CI, 0.72 to 0.79), and 0.64 (95% CI, 0.56 to 0.74) for incident major coronary events, ischemic stroke, and hemorrhagic stroke, respectively.[51] Another prospective analyses of CKB found that fresh fruit

For peer review only - http://bmjopen.bmj.com/site/about/guidelines.xhtml

intake reduced all-cause mortality, CVD mortality, cancer mortality and mortality from chronic obstructive pulmonary disease (COPD).[52] These findings may explain the difference of relationship between long sleep duration and hypertension among non-regular and regular fresh fruit consumers. The association between long sleep duration and hypertension was more pronounced in individuals who had insomnia symptoms than non-insomnia subgroup. Two factors may explain the differential associations. First, insomnia has been proved to be a risk factor for hypertension.[34] Among insomnia participants, long sleep duration was more likely to be an unusual schedule, which could affect health outcomes in humans. Second, the sample size of non-insomnia subgroup was much higher than that of the insomnia group, which may lead to statistical difference.

Our study had several strengths. This study was part of CKB, which was a large prospective cohort study in southwest China. CKB had a series of standardized procedures and good quality control measures, so the data was relatively reliable. The sample size was large in this study, which could be a representative population in southwest China. Although this study revealed an association between long sleep duration and hypertension, several potential limitations of this study should be noted and need to be addressed in future work. First, our study was a cross-sectional study, so the directionality of the causation could not be established which did not allow for inferences regarding causation to be made. Further prospective cohort studies would be needed to confirm this association. Second, the data of sleep duration was self-reported without more detailed and objective measures of sleep, which might have information bias and was less accurate than actigraphy and polysomnography. Third, although we have attempted to adjust for various potential confounders, our results may still be subject to residual confounding from unmeasured or unknown influencing factors.

CONCLUSIONS

In conclusion, this study identified long sleep duration is a risk factor for hypertension in adults of southwest China. However, this association was not found between short sleep duration and hypertension. It is important to sustain proper sleep duration to promote health. Further

For peer review only - http://bmjopen.bmj.com/site/about/guidelines.xhtml

prospective studies are required to confirm these findings.

For peer review only

For peer review only - http://bmjopen.bmj.com/site/about/guidelines.xhtml

Acknowledgments We thank Chinese Center for Disease Control and Prevention, Chinese Ministry of Health, National Health and Family Planning Commission of China, and 10 provincial/regional Health Administrative Departments. The most important acknowledgement is to the participants in the study and the members of the survey teams in each of the 10 regional centers, as well as to the project development and management teams based at Beijing, Oxford and the 10 regional centers.

Contributors XW and GL conceived and designed the paper. LL and ZC designed and supervised the conduct of the CKB study, obtained funding and together with YG and PP acquired the data. XC, XC and JJ analysed the data. XC, XC, QS and NZ drafted the manuscript. XC, XC, JJ, GL, XC, QS, NZ and XW contributed to the interpretation of the results and critical revision of the manuscript. All authors contributed to and approved the final manuscript. XW is the study guarantor.

Funding This work was supported by grants from the Tianfu famous doctor of Tianfu Ten Thousand People Plan in Sichuan Province, grants (2016YFC0900500, 2016YFC0900501, 2016YFC0900504) from the National Key Research and Development Program of China, grants from the Kadoorie Charitable Foundation in Hong Kong, grants (212946/Z/18/Z, 202922/Z/16/Z, 104085/Z/14/Z, 088158/Z/09/Z) from Wellcome Trust in the UK. The funders had no role in the study design, data collection, data analysis and interpretation, writing of the report or the decision to submit the article for publication.

Competing interests None declared.

Patient consent for publication Not required.

Ethics approval The Ethical Review Committee of the Chinese Center for Disease Control and Prevention (Beijing, China) (Ethics ID number of approval: 005/2004) and the Oxford Tropical Research Ethics Committee, University of Oxford (UK) (Ethics ID number of approval: OXTREC 025-04).

Provenance and peer review Not commissioned; externally peer reviewed.

Data availability statement The dataset supporting the conclusions of this article is available from the study website (http://www.ckbiobank.org), along with the access policy and

For peer review only
For peer review only - http://bmjopen.bmj.com/site/about/guidelines.xhtml

procedures.

For peer review only

For peer review only - http://bmjopen.bmj.com/site/about/guidelines.xhtml

REFERENCES

1 Organization WH. A global brief on hypertension : silent killer, global public health crisis: World Health Day 2013. In. Geneva: World Health Organization; 2013.

2 Wang Y, Peng X, Nie X*, et al.* Burden of hypertension in China over the past decades: Systematic analysis of prevalence, treatment and control of hypertension. *Eur J Prev Cardiol* 2016; 23:792-800.

3 Wang Z, Chen Z, Zhang L*, et al.* Status of Hypertension in China: Results From the China Hypertension Survey, 2012-2015. *Circulation* 2018; 137:2344-56.

4 Zhou M, Wang H, Zeng X*, et al.* Mortality, morbidity, and risk factors in China and its provinces, 1990-2017: a systematic analysis for the Global Burden of Disease Study 2017. *Lancet* 2019; 394:1145-58.

5 Cao D, Zhou Z, Si Y*, et al.* Prevalence and income-related equity in hypertension in rural China from 1991 to 2011: differences between self-reported and tested measures. *BMC Health Serv Res* 2019; 19:437.

6 Liu X, Zhang D, Liu Y*, et al.* Dose-Response Association Between Physical Activity and Incident Hypertension: A Systematic Review and Meta-Analysis of Cohort Studies. *Hypertension* 2017; 69:813-20.

7 Forman JP, Stampfer MJ, Curhan GC. Diet and lifestyle risk factors associated with incident hypertension in women. *Jama* 2009; 302:401-11.

8 Li H, Ren Y, Wu Y*, et al.* Correlation between sleep duration and hypertension: a dose-response meta-analysis. *J Hum Hypertens* 2019; 33:218-28.

9 Song Q, Liu X, Wang X*, et al.* Age- and gender-specific associations between sleep duration and incident hypertension in a Chinese population: the Kailuan study. *J Hum Hypertens* 2016; 30:503-7.

10 Guan Y, Zhang M, Zhang X*, et al.* Association between sleep duration and hypertension of migrant workers in China: a national cross-sectional surveillance study. *BMJ Open* 2019; 9:e031126.

11 Gangwisch JE, Heymsfield SB, Boden-Albala B*, et al.* Short sleep duration as a risk factor for hypertension: analyses of the first National Health and Nutrition Examination Survey. *Hypertension* 2006; 47:833-9.

12 Cappuccio FP, Stranges S, Kandala NB*, et al.* Gender-specific associations of short sleep duration with prevalent and incident hypertension: the Whitehall II Study. *Hypertension* 2007; 50:693-700.

13 Kim J, Jo I. Age-dependent association between sleep duration and hypertension in the adult Korean population. *Am J Hypertens* 2010; 23:1286-91.

14 Knutson KL, Van Cauter E, Rathouz PJ*, et al.* Association between sleep and blood pressure in midlife: the CARDIA sleep study. *Arch Intern Med* 2009; 169:1055-61.

15 Grandner M, Mullington JM, Hashmi SD*, et al.* Sleep Duration and Hypertension: Analysis of > 700,000 Adults by Age and Sex. *J Clin Sleep Med* 2018; 14:1031-39.

16 Fang J, Wheaton AG, Keenan NL*, et al.* Association of sleep duration and hypertension among US adults varies by age and sex. *Am J Hypertens* 2012; 25:335-41.

17 Gottlieb DJ, Redline S, Nieto FJ*, et al.* Association of usual sleep duration with hypertension: the Sleep Heart Health Study. *Sleep* 2006; 29:1009-14.

18 Magee CA, Kritharides L, Attia J*, et al.* Short and long sleep duration are associated with prevalent cardiovascular disease in Australian adults. *J Sleep Res* 2012; 21:441-7.

19 Li M, Yan S, Jiang S*, et al.* Relationship between sleep duration and hypertension in northeast China: a cross-sectional study. *BMJ Open* 2019; 9:e023916.

20 Chen Z, Lee L, Chen J*, et al.* Cohort profile: the Kadoorie Study of Chronic Disease in China (KSCDC). *Int J Epidemiol* 2005; 34:1243-9.

For peer review only - http://bmjopen.bmj.com/site/about/guidelines.xhtml

21 Chen Z, Chen J Fau - Collins R, Collins R Fau - Guo Y*, et al.* China Kadoorie Biobank of 0.5 million people: survey methods, baseline characteristics and long-term follow-up.

22 Chen X, Du H, Zhang J*, et al.* Adiposity and blood pressure among 55 000 relatively lean rural adults in southwest of China. *J Hum Hypertens* 2015; 29:522-9.

23 Lima-Costa MF, Peixoto SV, Rocha FL. Usual sleep duration is not associated with hypertension in Brazilian elderly: The Bambui Health Aging Study (BHAS). *Sleep Med* 2008; 9:806-7.

24 Zhou BF. Predictive Values of Body Mass Index and Waist Circumference for Risk Factors of Certain Related Diseases in Chinese Adults - Study on Optimal Cut-off Points of Body Mass Index and Waist Circumference in Chinese Adults. *Biomedical & Environmental Sciences* 2002; 11:S685-S93.

25 Tian X, Du H. Fruit consumption and physical activity in relation to all-cause and cardiovascular mortality among 70,000 Chinese adults with pre-existing vascular disease. PLoS One 2017; 12:e0173054..

26 Du H, Bennett D, Li L*, et al.* Physical activity and sedentary leisure time and their associations with BMI, waist circumference, and percentage body fat in 0.5 million adults: the China Kadoorie Biobank study. *Am J Clin Nutr* 2013; 97:487-96.

27 Zheng B, Yu C, Lin L*, et al.* Associations of domain-specific physical activities with insomnia symptoms among 0.5 million Chinese adults. *J Sleep Res* 2017; 26:330-37.

28 Gangwisch JE, Feskanich D, Malaspina D*, et al.* Sleep duration and risk for hypertension in women: results from the nurses' health study. *Am J Hypertens* 2013; 26:903-11.

29 Guo X, Zheng L, Wang J*, et al.* Epidemiological evidence for the link between sleep duration and high blood pressure: a systematic review and meta-analysis. *Sleep Med* 2013; 14:324-32.

30 Wang Q, Xi B, Liu M*, et al.* Short sleep duration is associated with hypertension risk among adults: a systematic review and meta-analysis. *Hypertens Res* 2012; 35:1012-8.

31 Makarem N, Shechter A, Carnethon MR*, et al.* Sleep Duration and Blood Pressure: Recent Advances and Future Directions. *Curr Hypertens Rep* 2019; 21:33.

32 Wang H, Zee P, Reid K*, et al.* Gender-specific association of sleep duration with blood pressure in rural Chinese adults. *Sleep Med* 2011; 12:693-9.

33 Kuciene R, Dulskiene V. Associations of short sleep duration with prehypertension and hypertension among Lithuanian children and adolescents: a cross-sectional study. *BMC Public Health* 2014; 14:255.

34 Jarrin DC, Alvaro PK, Bouchard MA*, et al.* Insomnia and hypertension: A systematic review. *Sleep Med Rev* 2018; 41:3-38.

35 Khazaie H, Negahban S, Ghadami MR, et al. Among middle-aged adults, snoring predicted hypertension independently of sleep apnoea. J Int Med Res 2018; 46:1187-96.

36 Lusardi P, Zoppi A, Preti P*, et al.* Effects of insufficient sleep on blood pressure in hypertensive patients: a 24-h study. *Am J Hypertens* 1999; 12:63-8.

37 Spiegel K, Tasali E, Penev P*, et al.* Brief communication: Sleep curtailment in healthy young men is associated with decreased leptin levels, elevated ghrelin levels, and increased hunger and appetite. *Ann Intern Med* 2004; 141:846-50.

38 Ayas NT, White DP, Al-Delaimy WK*, et al.* A prospective study of self-reported sleep duration and incident diabetes in women. *Diabetes Care* 2003; 26:380-4.

39 Ayas NT, White DP, Manson JE*, et al.* A prospective study of sleep duration and coronary heart disease in women. *Arch Intern Med* 2003; 163:205-9.

For peer review only - http://bmjopen.bmj.com/site/about/guidelines.xhtml

40 Suzuki E, Yorifuji T, Ueshima K, *et al.* Sleep duration, sleep quality and cardiovascular disease mortality among the elderly: a population-based cohort study. *Prev Med* 2009; 49:135-41.

41 Cappuccio FP, Cooper D, D'Elia L, *et al.* Sleep duration predicts cardiovascular outcomes: a systematic review and meta-analysis of prospective studies. *Eur Heart J* 2011; 32:1484-92.

42 Choi KM, Lee JS, Park HS, *et al.* Relationship between sleep duration and the metabolic syndrome: Korean National Health and Nutrition Survey 2001. *Int J Obes (Lond)* 2008; 32:1091-7.

43 Lopez-Garcia E, Faubel R, Guallar-Castillon P, *et al.* Self-reported sleep duration and hypertension in older Spanish adults. *J Am Geriatr Soc* 2009; 57:663-8.

44 Crowley K. Sleep and sleep disorders in older adults. *Neuropsychol Rev* 2011; 21:41-53.

45 Fernandez-Mendoza J, Vgontzas AN, Liao D, *et al.* Insomnia with objective short sleep duration and incident hypertension: the Penn State Cohort. *Hypertension* 2012; 60:929-35.

46 Budhiraja R, Roth T, Hudgel DW, *et al.* Prevalence and polysomnographic correlates of insomnia comorbid with medical disorders. *Sleep* 2011; 34:859-67.

47 Floras JS. Hypertension and Sleep Apnea. *Can J Cardiol* 2015; 31:889-97.

48 Kara B, Tenekeci EG. Sleep Quality and Associated Factors in Older Turkish Adults With Hypertension: A Pilot Study. *J Transcult Nurs* 2017; 28:296-305.

49 Liu RQ, Qian Z, Trevathan E, *et al.* Poor sleep quality associated with high risk of hypertension and elevated blood pressure in China: results from a large population-based study. *Hypertens Res* 2016; 39:54-9.

50 Fiorentini A, Valente R, Perciaccante A, *et al.* Sleep's quality disorders in patients with hypertension and type 2 diabetes mellitus. *Int J Cardiol* 2007; 114:E50-2.

51 Du H, Li L, Bennett D, *et al.* Fresh Fruit Consumption and Major Cardiovascular Disease in China. *N Engl J Med* 2016; 374:1332-43.

52 Du H, Li L, Bennett D, *et al.* Fresh fruit consumption and all-cause and cause-specific mortality: findings from the China Kadoorie Biobank. *Int J Epidemiol* 2017; 46:1444-55.

For peer review only

For peer review only - http://bmjopen.bmj.com/site/about/guidelines.xhtml

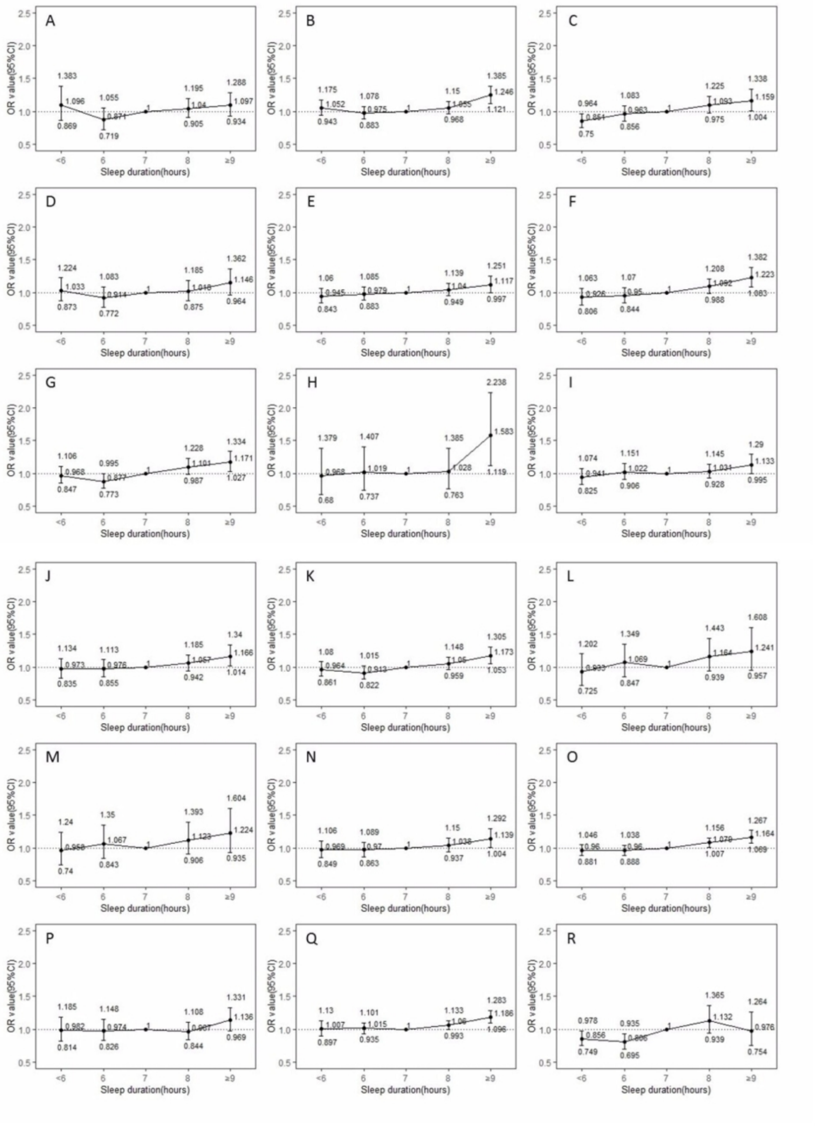

Figure 1 Adjusted relationships between sleep duration and hypertension stratified by health-related factors. ORs in each group are presented as points with 95% CIs represented by extended lines. The horizontal reference dashed line represents an OR value of '1'. The full line represents the effects of different sleep durations on hypertension. A, 30-44 years old subgroup; B, 45-59 years old subgroup; C, 60-79 years old subgroup; D, Never or almost never tea consumers; E, Only occasionally tea consumers; F, Usually at least once a week tea consumers; G, Never regular alcohol drinker; H, Ex-regular alcohol drinker; I, Occasional or seasonal alcohol drinker; J, Usually at least once a week alcohol drinker; K, Never smoker; L, Ex-regular smoker; M, Occasional smoker; N, Smoked daily or almost every day; O, Non-regular fresh fruit consumers; P, regular fresh fruit consumers; Q, insomnia subgroup; R, non-insomnia subgroup. Multivariable logistic model adjusted age, sex, education level, occupation, marital status, income, tea, smoking status, alcohol intake, MET, sedentary leisure time, fresh fruit consumption, insomnia, sleep snoring, BMI, WC, DM, stroke and CHD.

118x157mm (300 x 300 DPI)

For peer review only - http://bmjopen.bmj.com/site/about/guidelines.xhtml

STROBE Statement—Checklist of items that should be included in reports of ***cross-sectional studies***

	Item No	Recommendation	Page No
**Title and abstract**	1	(*a*) Indicate the study's design with a commonly used term in the title or the abstract	1
		(*b*) Provide in the abstract an informative and balanced summary of what was done and what was found	2
**Introduction**			
Background/rationale	2	Explain the scientific background and rationale for the investigation being reported	4
Objectives	3	State specific objectives, including any prespecified hypotheses	4
**Methods**			
Study design	4	Present key elements of study design early in the paper	5
Setting	5	Describe the setting, locations, and relevant dates, including periods of recruitment, exposure, follow-up, and data collection	5
Participants	6	(*a*) Give the eligibility criteria, and the sources and methods of selection of participants	5
Variables	7	Clearly define all outcomes, exposures, predictors, potential confounders, and effect modifiers. Give diagnostic criteria, if applicable	5-7
Data sources/ measurement	8*	For each variable of interest, give sources of data and details of methods of assessment (measurement). Describe comparability of assessment methods if there is more than one group	5-7
Bias	9	Describe any efforts to address potential sources of bias	5-7
Study size	10	Explain how the study size was arrived at	5
Quantitative variables	11	Explain how quantitative variables were handled in the analyses. If applicable, describe which groupings were chosen and why	5-7
Statistical methods	12	(*a*) Describe all statistical methods, including those used to control for confounding	7
		(*b*) Describe any methods used to examine subgroups and interactions	7
		(*c*) Explain how missing data were addressed	None
		(*d*) If applicable, describe analytical methods taking account of sampling strategy	5-7
		(*e*) Describe any sensitivity analyses	5-7
**Results**			
Participants	13*	(a) Report numbers of individuals at each stage of study—eg numbers potentially eligible, examined for eligibility, confirmed eligible, included in the study, completing follow-up, and analysed	7-10
		(b) Give reasons for non-participation at each stage	None
		(c) Consider use of a flow diagram	None
Descriptive data	14*	(a) Give characteristics of study participants (eg demographic, clinical, social) and information on exposures and potential confounders	7-10
		(b) Indicate number of participants with missing data for each variable of interest	7-10
Outcome data	15*	Report numbers of outcome events or summary measures	10-13

For peer review only

Main results	16	(*a*) Give unadjusted estimates and, if applicable, confounder-adjusted estimates and their precision (eg, 95% confidence interval). Make clear which confounders were adjusted for and why they were included	10-13
		(*b*) Report category boundaries when continuous variables were categorized	10-13
		(*c*) If relevant, consider translating estimates of relative risk into absolute risk for a meaningful time period	10-13
Other analyses	17	Report other analyses done—eg analyses of subgroups and interactions, and sensitivity analyses	12-13
**Discussion**			
Key results	18	Summarise key results with reference to study objectives	14-18
Limitations	19	Discuss limitations of the study, taking into account sources of potential bias or imprecision. Discuss both direction and magnitude of any potential bias	17
Interpretation	20	Give a cautious overall interpretation of results considering objectives, limitations, multiplicity of analyses, results from similar studies, and other relevant evidence	14-18
Generalisability	21	Discuss the generalisability (external validity) of the study results	14-18
**Other information**			
Funding	22	Give the source of funding and the role of the funders for the present study and, if applicable, for the original study on which the present article is based	19

*Give information separately for exposed and unexposed groups.

Note: An Explanation and Elaboration article discusses each checklist item and gives methodological background and published examples of transparent reporting. The STROBE checklist is best used in conjunction with this article (freely available on the Web sites of PLoS Medicine at http://www.plosmedicine.org/, Annals of Internal Medicine at http://www.annals.org/, and Epidemiology at http://www.epidem.com/). Information on the STROBE Initiative is available at www.strobe-statement.org.

For peer review only

BMJ Open

The association between sleep duration and hypertension in southwest China: a population-based cross-sectional study

Journal:	*BMJ Open*
Manuscript ID	bmjopen-2021-052193.R1
Article Type:	Original research
Date Submitted by the Author:	17-Sep-2021
Complete List of Authors:	Chang, Xiaoyu; Sichuan Center for Disease Control and Prevention, Department of Chronic and Non-communicable Disease Control and Prevention<br>Chen, Xiaofang; Chengdu Medical College, Department of Epidemiology and Statistics<br>Ji, John; Duke Kunshan University, Environmental Research Center; Duke University, Nicholas School of the Environment<br>Luo, Guojin; Pengzhou Center For Disease Control And Prevention<br>Chen, Xiaofang; Pengzhou Center For Disease Control And Prevention<br>Sun, Qiang; Pengzhou Center For Disease Control And Prevention<br>Zhang, Ningmei; Sichuan Center for Disease Control and Prevention<br>Guo, Yu; Chinese Academy of Medical Sciences<br>Pei, Pei; Chinese Academy of Medical Sciences<br>Li, Liming; Peking University, School of Public Health; Peking University, Peking University Center for Public Health and Epidemic Preparedness & Response<br>Chen, Zhengming; University of Oxford, Nuffield Department of Population Health<br>Wu, Xianping ; Sichuan Center for Disease Control and Prevention
**Primary Subject Heading**:	Epidemiology
Secondary Subject Heading:	Public health
Keywords:	Hypertension < CARDIOLOGY, SLEEP MEDICINE, EPIDEMIOLOGY, PUBLIC HEALTH

SCHOLARONE™
Manuscripts

I, the Submitting Author has the right to grant and does grant on behalf of all authors of the Work (as defined in the below author licence), an exclusive licence and/or a non-exclusive licence for contributions from authors who are: i) UK Crown employees; ii) where BMJ has agreed a CC-BY licence shall apply, and/or iii) in accordance with the terms applicable for US Federal Government officers or employees acting as part of their official duties; on a worldwide, perpetual, irrevocable, royalty-free basis to BMJ Publishing Group Ltd ("BMJ") its licensees and where the relevant Journal is co-owned by BMJ to the co-owners of the Journal, to publish the Work in this journal and any other BMJ products and to exploit all rights, as set out in our licence.

The Submitting Author accepts and understands that any supply made under these terms is made by BMJ to the Submitting Author unless you are acting as an employee on behalf of your employer or a postgraduate student of an affiliated institution which is paying any applicable article publishing charge ("APC") for Open Access articles. Where the Submitting Author wishes to make the Work available on an Open Access basis (and intends to pay the relevant APC), the terms of reuse of such Open Access shall be governed by a Creative Commons licence – details of these licences and which Creative Commons licence will apply to this Work are set out in our licence referred to above.

Other than as permitted in any relevant BMJ Author's Self Archiving Policies, I confirm this Work has not been accepted for publication elsewhere, is not being considered for publication elsewhere and does not duplicate material already published. I confirm all authors consent to publication of this Work and authorise the granting of this licence.

For peer review only

The association between sleep duration and hypertension in southwest China: a population-based cross-sectional study

Xiaoyu Chang,[1] Xiaofang Chen,[2] John S Ji,[3] Guojin Luo,[4] Xiaofang Chen,[4] Qiang Sun,[4] Ningmei Zhang,[1] Yu Guo,[5] Pei Pei,[5] Liming Li,[6] Zhengming Chen,[7] Xianping Wu[1]

Correspondence to Professor Xianping Wu; wwwuxp@163.com

Name: Xianping Wu; Postal address: No. 6 Zhongxue Road, Wuhou District, Chengdu, Sichuan Province, China (Postal Code: 610041); E-mail: wwwuxp@163.com; Telephone: 86-28-85589582; Fax numbers: 86-28-85582320.

Author affiliations

[1]Department of Chronic and Non-communicable Disease Control and Prevention, Sichuan Center for Disease Control and Prevention, Chengdu, China

[2]Department of Epidemiology and Statistics, Chengdu Medical College, Chengdu, China

[3]Environmental Research Center, Duke Kunshan University, Kunshan, China; Nicholas School of the Environment, Duke University, Durham, NC, USA

[4]Pengzhou Center for Disease Control and Prevention, Pengzhou, China

[5]Chinese Academy of Medical Sciences, Beijing, China

[6]Department of Epidemiology & Biostatistics, School of Public Health, Peking University Health Science Center , Beijing, China; Peking University Center for Public Health and Epidemic Preparedness & Response, Beijing, China

[7]Clinical Trial Service Unit and Epidemiological Studies Unit (CTSU), Nuffield Department of Population Health, University of Oxford, Oxford, UK

Word count: 4962

For peer review only - http://bmjopen.bmj.com/site/about/guidelines.xhtml

ABSTRACT

Objective Hypertension is a major risk factor and cause of many non-communicable diseases in China. While there have been studies on various diet and lifestyle risk factors, we do not know whether sleep duration has an association to blood pressure in southwest China. This predictor is useful in low-resource rural settings. We examined the association between sleep duration and hypertension in southwest China.

Design Population-based cross-sectional study

Setting This study was part of the baseline survey of a large ongoing prospective cohort study, the China Kadoorie Biobank. Participants were enrolled in 15 townships of Pengzhou city in Sichuan province during 2004-2008.

Participants 55 687 participants aged 30 to 79 years were included. Sleep duration was assessed by a self-reported questionnaire.

Main outcome measures Hypertension was defined as systolic blood pressure ≥ 140 mm Hg and/or diastolic blood pressure ≥ 90 mm Hg, or prior physician-diagnosed hypertension in hospitals at the township (community) level or above.

Results The prevalence of hypertension was 25.17%. The percentages of subjects with sleep durations of <6, 6, 7, 8 and ≥ 9 hours were 17.20%, 16.14%, 20.04%, 31.95% and 14.68%, respectively. In multivariable adjusted analyses, the increased odds ratios (95% CIs) of having hypertension was across those who reported ≥ 9 hours of sleep (men: 1.16, 1.04 -1.30; women: 1.19, 1.08 -1.32; general population: 1.17, 1.08 -1.26). The odds of hypertension was relatively flat until around 6.81 hours of sleep duration and then started to increase rapidly afterwards in subjects and a J-shaped pattern was observed. There was a U-shaped relationship between sleep duration and hypertension in females.

Conclusion Long sleep duration was significantly associated with hypertension and a J-shaped pattern was observed among rural adults in southwest China, independent of potential confounders. However, this association was not obvious between short sleep duration and hypertension.

Strengths and limitations of this study

For peer review only - http://bmjopen.bmj.com/site/about/guidelines.xhtml

This study examined the association between sleep duration and hypertension in southwest China, and this predictor was useful in low-resource rural settings.

A large sample of rural population was obtained, which could represent most of the rural places in southwest China.

A broad range of covariates were controlled in the analysis, including age, sex, education, occupation, marital status, income, tea, smoking, alcohol, MET, sedentary leisure time, fresh fruit consumption, insomnia, sleep snoring, BMI, WC, DM, stroke and CHD.

The dose-response relationship between sleep duration and hypertension was explored and visualized by restricted cubic splines regression.

The sleep duration was self-reported without more detailed and objective measures of sleep, which was less accurate than actigraphy and polysomnography.

For peer review only - http://bmjopen.bmj.com/site/about/guidelines.xhtml

INTRODUCTION

Hypertension is a condition in which the blood vessels have persistently raised pressure.[1] It is the leading risk factor for global cardiovascular disease burden and mortality.[2] In China, hypertension is also a serious public health problem. The China hypertension survey showed that the prevalence of hypertension and pre-hypertension were 23.2% and 41.3% among Chinese adults, which means that there is one hypertensive patient per four people.[3] High systolic blood pressure was the leading risk factors contributing to deaths and DALYs.[4] In addition, hypertension is a growing problem not only in urban but also in rural China. The prevalence of hypertension has sharply increased in rural China,[5] which is similar in rural and urban settings (23.4% versus 23.1%).[3] Disease burden of hypertension is increasing globally, especially as places of poverty become more prosperous.[2]

Previous studies about influencing factors of hypertension mainly focus on diet (high dietary sodium intake), exercise and other lifestyle factors,[6 7] while some studies in recent years documented the relationship between sleep and hypertension. Sleep status influence blood pressure by altering autonomic nervous system function and other physiologic events. Unhealthy sleep status such as sleep apnea, insomnia, long and short sleep duration can increase risk of hypertension. Even though a variety of studies examined sleep duration to be associated with hypertension, the results were not completely consistent. Some studies showed that short sleep duration increased the risk of hypertension,[8-14] others showed that long sleep duration was related to hypertension or the association were J- or U-shaped curves.[15-18] However, most of the study populations were from western countries. A small number of studies were conducted in Asian countries,[9 10 13 19] and there was limited information about the association between sleep duration and hypertension in southwest China. Therefore, the aim of this study is to analyze the baseline data from a large prospective cohort study of CKB to explore the association of sleep duration with hypertension among more than 50 000 adults in Southwest China.

For peer review only - http://bmjopen.bmj.com/site/about/guidelines.xhtml

SUBJECTS AND METHODS

Study population

This study utilizes a large, ongoing prospective cohort study, the China Kadoorie Biobank (CKB). The baseline survey of CKB was conducted in five urban and five rural areas across China from 2004 to 2008. Our data was derived from the baseline survey of CKB in Sichuan province. A total of 55 687 residents aged 30 to 79 years in 15 townships of Pengzhou city in Sichuan province had completed baseline information collection, including a questionnaire, anthropometric measurements, blood sample collection, and a written informed consent. More details about the study design are available in previous publications.[20-22]

Data collection

The baseline survey took place in temporary study clinics, specially set up near participants' residential areas. Information on sociodemographic characteristics (age, sex, education level, occupation, annual household income), behavioural risk factors for chronic diseases (diet, tea consumption, smoking status, alcohol intake, physical activity, sedentary leisure time, sleep), and medical history were collected through an interviewer-administered computerized questionnaire by face-to-face interview with trained interviewers according to standard protocols and procedures. A range of physical measurements were also undertaken, including height, weight, waist circumference and so on.

Outcome measurement ascertainment

Participants were asked to report the number of hours they slept a day during the last 12 months. Sleep duration was assessed by a self-reported questionnaire with the following question: "On average, how many hours do you typically sleep per day (including daytime naps)?". Respondents could report in only 1-hour increments. To maintain consistency with previous literatures,[10 17 18 23] sleep duration was categorized as five groups of <6, 6, 7 (which regarded as the reference group for our analysis), 8 and ≥ 9 hours.

As described in the previous study,[22] blood pressure was measured twice using a UA-779

For peer review only - http://bmjopen.bmj.com/site/about/guidelines.xhtml

digital monitor recommended by the British Hypertension Society (A & D Instruments Ltd., Abingdon, UK). If the systolic blood pressure (SBP) difference between the first two measurements was more than 10 mm Hg, a third measurement was obtained and the last two measurements were recorded. The mean of two blood pressure measurements was used for this study. Hypertension was defined as systolic blood pressure (SBP) ≥ 140 mm Hg and/or diastolic blood pressure (DBP) ≥ 90 mm Hg, or prior physician-diagnosed hypertension in hospitals at the township (community) level or above.

Several variables were used both as covariates in adjusted models and as variables for stratification. Sociodemographic characteristics included age at baseline survey (30-79 years old), sex (male or female), education level (no formal school, primary school, middle school, high school and above), occupation (famer or others), marital status (married or never married/widowed/separated/divorced), annual household income (< 5 000, 5 000~9 999, ≥10 000, RMB, yuan). Behavioural risk factors included tea consumption (never or almost never, only occasionally, usually at least once a week), smoking status (never, ex-regular, occasionally, daily or almost every day), alcohol intake (never regular, ex-regular, occasionally or seasonally, usually at least once a week), fresh fruit consumption (daily, 4-6 days per week, 1-3 days per week , monthly, never/rarely), physical activity, sedentary leisure time (hours/week), insomnia symptoms (yes or no), sleep snoring (never, occasionally, usually) , waist circumference (WC) and body mass index (BMI). Medical history included diabetes mellitus (DM), stroke and coronary heart disease (CHD). BMI was calculated as weight in kilograms (kg) / height in metres $(m)^2$. According to the Guidelines for Prevention and Control of Overweight and Obesity in Adults in China,[24] BMI was divided into four groups: underweight (BMI <18.5 kg/m^2), normal weight (18.5 ≤ BMI <24 kg/m^2), overweight (24 ≤ BMI <28 kg/m^2) and obesity (BMI ≥28 kg/m^2). Waist circumference beyond 85 cm for men and 80 cm for women were recommended as central obesity.

Information about type, frequency, and duration of occupational, commuting-related, household and active recreational (leisure-time) physical activities were used to calculate total physical activity in metabolic equivalents (MET, hours/day).[25 26] Sedentary leisure time

For peer review only - http://bmjopen.bmj.com/site/about/guidelines.xhtml

(hours/week) was assessed by the question of "About how many hours per week did you watch TV or read?". Insomnia symptoms were determined by three questions: Taking >30 minutes to fall asleep after going to bed or waking up in the middle of the night; Waking up early and not being able to go back to sleep; Having difficulty staying alert while at work, eating or meeting people during daytime. Those reporting "Yes" for one or more of the three questions were classified as having insomnia symptoms.[27] Participants consumed fresh fruit more than 3 days/week were defined as regular consumers, otherwise, those were never or occasionally consumers.[25]

Statistical Analysis

Continuous variables were expressed as mean and standard deviation (SD). Frequency and percentage distributions were reported for categorical variables. Continuous variables included age, MET, sedentary leisure time, BMI, WC, SBP, DBP. Categorical variables included age groups, gender, education level, occupation, marital status, income, tea consumption, smoking status, alcohol intake, fresh fruit consumption, insomnia, sleep snoring, BMI groups, DM, stroke and CHD. Prevalence of hypertension was calculated for the overall population and different subgroups, including sleep duration and some potential confounding factors. Chi-square tests were conducted to test for group differences in prevalence or percentage, and the Cochran-Armitage tests for trend were used to examine the trends for ordered categorical variables. The analysis of variance was used for continuous variables of study participants by different sleep durations. The statistical significance of associations for the continuous and categorical variables with sleep duration was estimated. Multivariable logistic regression models were used to calculate the odds ratios (ORs) and 95% confidence intervals (CIs) for hypertension between sleep duration subgroups adjusted for potential confounders, including age, gender, education level, occupation, marital status, income, tea consumption, smoking status, alcohol intake, MET, sedentary leisure time, fresh fruit consumption, insomnia, sleep snoring, BMI, WC, DM, stroke and CHD. In addition, we performed subgroup analysis stratified by health-related factors, such as age, sex, tea consumption, smoking status, alcohol

For peer review only - http://bmjopen.bmj.com/site/about/guidelines.xhtml

intake, insomnia. We also used restricted cubic splines regression with five knots at the 5th, 35th, 50th, 65th, and 95th centiles to flexibly model the association of sleep duration and hypertension. Multiple linear regression model was used to explore the relationship for sleep duration with SBP and DBP. A two-tailed *P* value of less than 0.05 was considered statistically significant. All data processing and statistical analyses were performed using Statistical Analysis Software, version 9.4 (SAS Institute, Cary, NC) and R version 4.0.3 (R Foundation for Statistical Computing, Vienna, Austria).

Patient and public involvement

No patients were involved in the present study, the interpretation of study results or write-up of the manuscript. The results were presented to study participants by the website of the CKB (http://www.ckbiobank.org/site/) and newsletters.

For peer review only

For peer review only - http://bmjopen.bmj.com/site/about/guidelines.xhtml

RESULTS

Different demographic characteristics of study population across sleep duration groups

The baseline characteristics of study population for subgroups according to sleep duration were presented in **Table 1**. A total of 55 687 adults completed baseline information, including 34 372 women and 21 315 men. The mean (±SD) age of study population was 51.03±10.54 years (range: 30–79 years). The mean (±SD) sleep duration was 7.05±1.68 hours, which were 7.07±1.61 hours in men and 7.04±1.72 hours in women. The overall means (±SD) of SBP and DBP were 129.24±19.01 mm Hg and 77.23 ± 10.23 mm Hg, respectively. The percentages of the subjects with sleep durations of <6, 6, 7, 8 and ≥ 9 hours were 17.20%, 16.14%, 20.04%, 31.95% and 14.68%, respectively.

Individuals with less hours of sleep per day were more likely to be female, eldly, low education level, famer, single, low annual household income, never or almost never tea consumers, low frequency of fresh fruit consumption, low BMI. Short sleepers were more likely to be DM, CHD, stroke or hypertensive subjects. With the increase of age, the proportion of subjects who slept for equal to or more than 8 hours decreased, while the proportion of subjects who slept for equal to or less than 6 hours increased. With the increase of education level, the proportion of subjects with less than 6 hours of sleep duration decreased. With the increase of annual household income and tea consumption, the proportion of subjects who slept for less than 6 hours decreased. With the increase of BMI and fresh fruit consumption, the proportion of subjects who slept for less than 6 hours decreased, while the proportion of subjects who slept for equal to or more than 9 hours increased.

For peer review only - http://bmjopen.bmj.com/site/about/guidelines.xhtml

Table 1 Distributions of sleep duration by categorical basic characteristics and descriptive statistics of continuous covariates in the sleep duration groups

Variables	Sleep duration(hours) <6	6	7	8	≥9	Overall	χ ^{2}/F	P value
Number of subjects, n(%)	9576(17.2)	8988(16.14)	11159(20.04)	17790(31.95)	8174(14.68)	55687(100)		
Gender, n(%)								
Female	6271(18.24)	5273(15.34)	6594(19.18)	10898(31.71)	5336(15.52)	34372(100)	171.051	<.0001
Male	3305(15.51)	3715(17.43)	4565(21.42)	6892(32.33)	2838(13.31)	21315(100)		
Age(years), n(%)								
30~39	604(6.21)	998(10.27)	1802(18.54)	4142(42.60)	2176(22.38)	9722(100)	4406.276	<.0001
40~49	1651(11.01)	2087(13.91)	3170(21.13)	5523(36.82)	2571(17.14)	15002(100)		
50~59	3560(19.69)	3278(18.13)	3815(21.11)	5262(29.11)	2161(11.96)	18076(100)		
60~69	2775(27.41)	2050(20.25)	1962(19.38)	2340(23.12)	996(9.84)	10123(100)		
70~79	986(35.67)	575(20.80)	410(14.83)	523(18.92)	270(9.77)	2764(100)		
Education level, n(%)								
No formal school	2610(30.38)	1555(18.10)	1452(16.90)	1942(22.60)	1033(12.02)	8592(100)	2440.546	<.0001
Primary school	5146(18.53)	4759(17.14)	5539(19.95)	8401(30.26)	3920(14.12)	27765(100)		
Middle school	1444(9.70)	2040(13.70)	3059(20.55)	5722(38.44)	2621(17.61)	14886(100)		
High school and above	376(8.46)	634(14.27)	1109(24.95)	1725(38.82)	600(13.50)	4444(100)		
Occupation, n(%)								
Famer	8674(17.73)	7862(16.07)	9629(19.68)	15531(31.74)	7236(14.79)	48932(100)	102.183	<.0001
Others	902(13.35)	1126(16.67)	1530(22.65)	2259(33.44)	938(13.89)	6755(100)		
Marital status, n(%)								
Married	8040(15.91)	7990(15.81)	10236(20.26)	16610(32.87)	7650(15.14)	50526(100)	797.729	<.0001
Never Married/Widowed /Separated/Divorced	1536(29.76)	998(19.34)	923(17.88)	1180(22.86)	524(10.15)	5161(100)		
Annual household income (RMB, yuan) , n(%)								
<5,000	3730(24.61)	2454(16.19)	2761(18.22)	3996(26.37)	2215(14.61)	15156(100)	991.831	<.0001
5000~9,999	3145(15.98)	3168(16.09)	3867(19.65)	6532(33.18)	2972(15.10)	19684(100)		
≥10,000	2701(12.96)	3366(16.15)	4531(21.73)	7262(34.83)	2987(14.33)	20847(100)		
Tea consumption, n(%)								
Never or almost never	2311(23.51)	1534(15.61)	1768(17.99)	2692(27.39)	1525(15.51)	9830(100)	581.800	<.0001
Only occasionally	4822(17.76)	4488(16.53)	5385(19.83)	8695(32.02)	3764(13.86)	27154(100)		
Usually at least once a week	2443(13.06)	2966(15.86)	4006(21.42)	6403(34.24)	2885(15.43)	18703(100)		
Smoking status, n(%)								
Never	4896(16.38)	4418(14.78)	5771(19.30)	9998(33.44)	4814(16.10)	29897(100)	350.690	<.0001
Ex-regular smoker	835(22.14)	675(17.90)	786(20.84)	996(26.41)	479(12.70)	3771(100)		
Occasional smoker	892(20.14)	827(18.67)	892(20.14)	1272(28.71)	547(12.35)	4430(100)		
Daily or almost every day	2953(16.79)	3068(17.44)	3710(21.09)	5524(31.41)	2334(13.27)	17589(100)		
Alcohol intake, n(%)								
Never regular drinker	3591(18.80)	2926(15.32)	3575(18.72)	6031(31.57)	2979(15.60)	19102(100)	204.099	<.0001

For peer review only - http://bmjopen.bmj.com/site/about/guidelines.xhtml

Ex-regular drinker	463(22.43)	371(17.97)	439(21.27)	512(24.81)	279(13.52)	2064(100)		
Occasional or seasonal drinker	3548(16.40)	3456(15.97)	4439(20.51)	7118(32.89)	3078(14.22)	21639(100)		
Usually at least once a week	1974(15.32)	2235(17.35)	2706(21.01)	4129(32.05)	1838(14.27)	12882(100)		
Fresh fruit consumption, n(%)								
Daily	736(11.53)	863(13.52)	1376(21.56)	2218(34.76)	1188(18.62)	6381(100)	1185.806	<.0001
4-6 days per week	716(12.75)	814(14.50)	1134(20.20)	1931(34.39)	1020(18.17)	5615(100)		
1-3 days per week	3688(15.35)	3720(15.49)	4654(19.38)	8347(34.75)	3611(15.03)	24020(100)		
Monthly	3401(21.93)	2890(18.63)	3210(20.69)	4262(27.48)	1748(11.27)	15511(100)		
Never/rarely	1035(24.88)	701(16.85)	785(18.87)	1032(24.81)	607(14.59)	4160(100)		
BMI(Kg/m2), n(%)								
<18.5	625(25.31)	436(17.66)	462(18.71)	642(26.00)	304(12.31)	2469(100)	161.222	<.0001
18.5~23.9	5523(17.33)	5094(15.99)	6399(20.08)	10209(32.04)	4642(14.57)	31867(100)		
24~27.9	2700(16.12)	2718(16.23)	3393(20.26)	5416(32.34)	2519(15.04)	16746(100)		
≥28	728(15.81)	740(16.07)	905(19.65)	1523(33.07)	709(15.40)	4605(100)		
DM, n(%)								
No	9050(16.89)	8606(16.06)	10751(20.07)	17257(32.21)	7908(14.76)	53572(100)	118.858	<.0001
Yes	526(24.87)	382(18.06)	408(19.29)	533(25.20)	266(12.58)	2115(100)		
CHD, n(%)								
No	9501(17.15)	8929(16.11)	11101(20.03)	17734(32.00)	8147(14.70)	55412(100)	37.361	<.0001
Yes	75(27.27)	59(21.45)	58(21.09)	56(20.36)	27(9.82)	275(100)		
Stroke, n(%)								
No	9509(17.17)	8941(16.14)	11115(20.06)	17719(31.99)	8112(14.64)	55396(100)	23.279	0.0001
Yes	67(23.02)	47(16.15)	44(15.12)	71(24.40)	62(21.31)	291(100)		
Hypertension, n(%)								
No	6530(15.67)	6527(15.66)	8473(20.33)	13865(33.27)	6278(15.06)	41673(100)	362.709	<.0001
Yes	3046(21.74)	2461(17.56)	2686(19.17)	3925(28.01)	1896(13.53)	14014(100)		
Age(years), Mean (SD)	56.30±9.94	53.37±10.19	50.8±10.07	48.60±10.09	47.9±10.31	51.03±10.54	1224.030	<.0001
MET(hours/day), Mean (SD)	21.08±11.47	21.98±11.89	22.67±12.03	22.72±11.65	21.12±11.70	22.07±11.76	51.170	<.0001
WC(cm), Mean (SD)	77.45±9.27	78.27±9.11	78.16±8.98	78.07±8.96	77.99±9.05	78.00±9.06	11.800	<.0001
BMI(Kg/m2), Mean (SD)	23.00±3.29	23.29±3.21	23.32±3.18	23.38±3.15	23.39±3.19	23.29±3.20	25.120	<.0001
Sedentary leisure time(hours/week), Mean (SD)	25.15±10.22	25.83±10.45	25.98±10.50	26.85±10.53	27.28±11.39	26.28±10.61	64.490	<.0001
SBP(mmHg), Mean (SD)	132.28±20.24	130.23±19.23	128.61±18.73	127.81±18.18	128.52±18.96	129.24±19.01	99.140	<.0001
DBP(mmHg), Mean (SD)	77.65±10.51	77.34±10.34	76.96±10.24	77.02±9.97	77.45±10.28	77.23±10.23	8.940	<.0001

For peer review only

For peer review only - http://bmjopen.bmj.com/site/about/guidelines.xhtml

The percentages in table are row percent. χ^2 tests were conducted to test for differences in percentage for categorical variables.One-way anova tests were conducted to test for differences in means for categorical variables. Abbreviations: BMI, body mass index; DM, diabetes mellitus; CHD, Coronary Heart Disease; WC, waist circumference; MET, Metabolic Equivalent; SBP, Systolic Blood Pressure; DBP, diastolic blood pressure.

The prevalence of hypertension among adults in southwest China

Results of prevalence of hypertension in different gender groups among 55687 adults were presented in **Table 2**. 25.17% of 55687 adults had hypertension. A quarter of participants were considered hypertensive, slightly more in men (28.67%) than in women (22.99%). The prevalence of hypertension in both men and women increased with age. The prevalence of hypertension decreased with increased education level. The prevalence of hypertension in both males and females decreased with increased annual household income and tea consumption. Among different alcohol intake and smoking status groups, the highest prevalences of hypertension were observed in ex-regular drinker and ex-regular smoker in males and females.

The prevalence of hypertension increased with decreased fresh fruit consumption in males and females. The prevalence of hypertension in both males and females increased with BMI and sleep snoring frequency. Higher prevalence of hypertension was also associated with insomnia, DM, stroke, CHD in both males and females. The prevalence of hypertension in DM patients (48.98%) was twice as high as that in non-DM subjects (24.23%). The prevalence of hypertension in stroke patients (73.54%) was nearly triple that in non-stroke subjects (24.91%). The prevalence of hypertension in both males and females decreased with sleep duration.

For peer review only - http://bmjopen.bmj.com/site/about/guidelines.xhtml

Table 2 Prevalence of hypertension in different gender groups among 55687 adults in southwest China

	Male		Female		Overall	
**Variables**	**n(Prevalence %)**	**P value**	**n(Prevalence %)**	**P value**	**n(Prevalence %)**	**P value**
Total	6111(28.67)	-	7903(22.99)	-	14014(25.17)	<.0001
Age(years)						
30~39	417(12.76)	<.0001	331(5.13)	<.0001	748(7.69)	<.0001
40~49	905(17.47)		1259(12.82)		2164(14.42)	
50~59	1965(28.45)		3243(29.04)		5208(28.81)	
60~69	2115(45.98)		2361(42.75)		4476(44.22)	
70~79	709(52.21)		709(50.43)		1418(51.30)	
Education level						
No formal school	847(41.52)	<.0001	2410(36.78)	<.0001	3257(37.91)	<.0001
Primary school	3481(31.07)		3994(24.12)		7475(26.92)	
Middle school	1235(21.21)		1190(13.13)		2425(16.29)	
High school and above	548(24.37)		309(14.08)		857(19.28)	
Annual household income(RMB, yuan)						
<5,000	2129(37.15)	<.0001	3000(31.83)	<.0001	5129(33.84)	<.0001
5,000~9,999	1852(26.25)		2563(20.29)		4415(22.43)	
≥10,000	2130(24.97)		2340(19.00)		4470(21.44)	
Tea consumption						
Never or almost never	482(33.04)	0.0207	2335(27.89)	<.0001	2817(28.66)	0.0281
Only occasionally	1908(28.19)		4396(21.56)		6304(23.22)	
Usually at least once a week	3721(28.43)		1172(20.87)		4893(26.16)	
Smoking status						
Never	726(28.96)	<.0001	5737(20.95)	<.0001	6463(21.62)	<.0001
Ex-regular smoker	857(35.34)		520(38.63)		1377(36.52)	
Occasional smoker	527(25.41)		710(30.14)		1237(27.92)	
Smoked daily or almost every day	4001(27.96)		936(28.54)		4937(28.07)	
Alcohol intake						
Never regular drinker	663(28.54)	<.0001	4175(24.88)	<.0001	4838(25.33)	<.0001
Ex-regular drinker	572(40.97)		257(38.47)		829(40.16)	
Occasional or seasonal drinker	1646(24.00)		2830(19.14)		4476(20.68)	
Usually at least once a week	3230(30.08)		641(29.91)		3871(30.05)	

For peer review only

For peer review only - http://bmjopen.bmj.com/site/about/guidelines.xhtml

Fresh fruit consumption						
Daily	549(26.99)	<.0001	819(18.84)	<.0001	1368(21.44)	<.0001
4-6 days per week	482(26.41)		755(19.92)		1237(22.03)	
1-3 days per week	2387(26.61)		3249(21.59)		5636(23.46)	
Monthly	1957(30.08)		2364(26.25)		4321(27.86)	
Never/rarely	736(37.13)		716(32.87)		1452(34.90)	
Insomnia						
No	4650(27.66)	<.0001	5244(21.01)	<.0001	9894(23.69)	<.0001
Yes	1461(32.42)		2659(28.24)		4120(29.59)	
Sleep snoring						
Never	2007(25.39)	<.0001	3391(18.08)	<.0001	5398(20.25)	<.0001
Occasionally	1481(27.88)		1870(24.94)		3351(26.16)	
Usually	2623(32.39)		2642(32.54)		5265(32.47)	
BMI (Kg/m2)						
<18.5	177(19.80)	<.0001	254(16.13)	<.0001	431(17.46)	<.0001
18.5~23.9	3331(24.51)		3170(17.34)		6501(20.40)	
24~27.9	1999(35.59)		3080(27.68)		5079(30.33)	
≥28	604(49.67)		1399(41.28)		2003(43.50)	
DM						
No	5726(27.90)	<.0001	7252(21.94)	<.0001	12978(24.23)	<.0001
Yes	385(48.67)		651(49.17)		1036(48.98)	
CHD						
No	6049(28.54)	<.0001	7839(22.91)	<.0001	13888(25.06)	<.0001
Yes	62(52.99)		64(40.51)		126(45.82)	
Stroke						
No	5999(28.35)	<.0001	7801(22.79)	<.0001	13800(24.91)	<.0001
Yes	112(73.20)		102(73.91)		214(73.54)	
Sleep duration(hours)						
<6	1113(33.68)	<.0001	1933(30.82)	<.0001	3046(31.81)	<.0001
6	1108(29.83)		1353(25.66)		2461(27.38)	
7	1263(27.67)		1423(21.58)		2686(24.07)	
8	1803(26.16)		2122(19.47)		3925(22.06)	
≥9	824(29.03)		1072(20.09)		1896(23.20)	

"n(Prevalence,%)" represents the number of hypertension and the prevalence. χ^2 tests were conducted to test for differences in prevalence for unordered categorical variables and Chi-square tests for trend were used to examine the trends for ordered categorical variables.

For peer review only

Results of prevalence of hypertension and distribution of hypertension patients in different sleep duration groups were presented in **Table 3**. In different sleep groups, the prevalences of hypertension were more in men than in women.The prevalence of hypertension increased with age. The prevalence of hypertension decreased with increased fresh fruit consumption. Among different alcohol intake and smoking status groups, the highest prevalences of hypertension were observed in ex-regular drinker and ex-regular smoker in different sleep duration groups. Among 7 and 8 hours sleepers, the prevalence of hypertension was higher among insomnia subjects than that among non-insomnia groups.

The percentages of the hypertension patients with sleep durations of <6, 6, 7, 8 and ≥ 9 hours were 21.74%, 17.56%, 19.17%, 28.00% and 13.53%, respectively. Patients with less hours of sleep were more likely to be female, eldly, never or almost never tea consumers, low frequency of fresh fruit consumption. With the increase of age, the proportion of hypertension patients who slept for equal to or more than 8 hours decreased.

For peer review only

For peer review only - http://bmjopen.bmj.com/site/about/guidelines.xhtml

Table 3 Prevalence of hypertension and distribution of hypertension patients in different sleep duration groups among 55687 adults in southwest China

	**Prevalence of hypertension(%)**					n(%)						
**Variables**	**<6**	**6**	**7**	**8**	**≥9**	**<6**	**6**	**7**	**8**	**≥9**	**overall**	**P value**
Total	31.81	27.38	24.07	22.06	23.20	3046(21.74)	2461(17.56)	2686(19.17)	3925(28.00)	1896(13.53)	14014(100)	<.0001
Gender												
Female	30.82	25.66	21.58	19.47	20.09	1933(24.46)	1353(17.12)	1423(18.01)	2122(26.85)	1072(13.56)	7903(100)	
Male	33.68	29.83	27.67	26.16	29.03	1113(18.21)	1108(18.13)	1263(20.67)	1803(29.51)	824(13.48)	6111(100)	
P value	0.0044	<.0001	<.0001	<.0001	<.0001							
Age(years)												
30~39	7.45	7.52	8.49	7.48	7.58	45(6.02)	75(10.03)	153(20.45)	310(41.44)	165(22.06)	748(100)	<.0001
40~49	15.38	13.18	13.85	14.39	15.60	254(11.74)	275(12.71)	439(20.29)	795(36.73)	401(18.53)	2164(100)	
50~59	30.17	27.79	27.10	28.18	32.67	1074(20.62)	911(17.49)	1034(19.85)	1483(28.48)	706(13.56)	5208(100)	
60~69	42.56	44.00	43.48	45.47	47.79	1181(26.39)	902(20.15)	853(19.06)	1064(23.77)	476(10.63)	4476(100)	
70~79	49.9	51.83	50.49	52.20	54.81	492(34.70)	298(21.01)	207(14.60)	273(19.25)	148(10.44)	1418(100)	
P value	<.0001	<.0001	<.0001	<.0001	<.0001							
Tea consumption												
Never or almost never	35.05	29.14	27.32	24.63	27.15	810(28.75)	447(15.87)	483(17.15)	663(23.53)	414(14.70)	2817(100)	<.0001
Only occasionally	30.38	26.23	22.10	19.79	19.95	1465(23.24)	1177(18.67)	1190(18.88)	1721(27.30)	751(11.91)	6304(100)	
Usually at least once a week	31.56	28.22	25.29	24.07	25.34	771(15.76)	837(17.11)	1013(20.70)	1541(31.49)	731(14.94)	4893(100)	
P value	0.0117	0.9427	0.7805	0.1173	0.955							
Smoking status												
Never	29.31	23.74	20.90	18.49	19.19	1435(22.20)	1049(16.23)	1206(18.66)	1849(28.61)	924(14.30)	6463(100)	<.0001
Ex-regular smoker	39.16	38.07	34.10	34.74	37.37	327(23.75)	257(18.66)	268(19.46)	346(25.13)	179(13.00)	1377(100)	
Occasional smoker	32.96	29.87	25.45	25.79	25.78	294(23.77)	247(19.97)	227(18.35)	328(26.51)	141(11.40)	1237(100)	

For peer review only

Smoked daily or almost every day	33.53	29.60	26.55	25.38	27.93	990(20.05)	908(18.39)	985(19.95)	1402(28.40)	652(13.21)	4937(100)	
P value	0.0001	<.0001	<.0001	<.0001	<.0001							
Alcohol consumption												
Never regular drinker	33.14	26.73	24.31	22.22	22.05	1190(24.60)	782(16.16)	869(17.96)	1340(27.70)	657(13.58)	4838(100)	<.0001
Ex-regular drinker	40.60	41.24	36.90	37.70	47.67	188(22.68)	153(18.46)	162(19.54)	193(23.28)	133(16.04)	829(100)	
Occasional or seasonal drinker	27.73	23.87	19.71	17.43	17.90	984(21.98)	825(18.43)	875(19.55)	1241(27.73)	551(12.31)	4476(100)	
Usually at least once a week	34.65	31.36	28.82	27.88	30.20	684(17.67)	701(18.11)	780(20.15)	1151(29.73)	555(14.34)	3871(100)	
P value	<.0001	<.0001	<.0001	<.0001	<.0001							
Fresh fruit consumption												
Daily	28.13	23.41	22.97	18.49	19.61	207(15.13)	202(14.77)	316(23.10)	410(29.97)	233(17.03)	1368(100)	<.0001
4-6 days per week	26.26	26.41	21.34	19.63	20.88	188(15.20)	215(17.38)	242(19.56)	379(30.64)	213(17.22)	1237(100)	
1-3 days per week	29.34	25.56	23.18	20.97	21.43	1082(19.20)	951(16.87)	1079(19.14)	1750(31.06)	774(13.73)	5636(100)	
Monthly	34.49	29.55	24.64	24.61	25.97	1173(27.15)	854(19.76)	791(18.31)	1049(24.27)	454(10.51)	4321(100)	
Never/rarely	38.26	34.09	32.87	32.66	36.57	396(27.27)	239(16.46)	258(17.77)	337(23.21)	222(15.29)	1452(100)	
P value	<.0001	<.0001	<.0001	<.0001	<.0001							
Insomnia												
No	30.70	27.66	23.59	21.77	23.06	634(6.41)	1621(16.38)	2248(22.72)	3617(36.56)	1774(17.93)	9894(100)	<.0001
Yes	32.11	26.85	26.89	26.24	25.42	2412(58.54)	840(20.39)	438(10.63)	308(7.48)	122(2.96)	4120(100)	
P value	0.2228	0.4132	0.004	0.0004	0.2347							

"n(%)" represents the number of hypertension and the percentages. The percentages in table are row percent. χ^2 tests were conducted to test for differences in percentage for categorical variables.

χ^2 tests were conducted to test for differences in prevalence for unordered categorical variables and Chi-square tests for trend were used to examine the trends for ordered categorical variables.

For peer review only

For peer review only - http://bmjopen.bmj.com/site/about/guidelines.xhtml

The relationship between sleep duration and hypertension

Results of the multivariable logistic regression analyses examined relationship between sleep duration and hypertension were presented in **Table 4**. Compared to 7-hours sleepers (reference group), the unadjusted analyses showed that short sleepers (< 6 hours and 6 hours) having higher odds of hypertension (OR = 1.47 and 1.19, respectively; $P < 0.05$). After adjustment for age, sex, education level, occupation, marital status, annual household income, tea consumption, smoking status, alcohol intake, MET, sedentary leisure time, fresh fruit consumption, insomnia, sleep snoring, BMI, WC, DM, stroke and CHD, the increased odds of having hypertension was across those who reported ≥ 9 hours of sleep (OR = 1.17, 95% CI: 1.08-1.26; $P < 0.05$). However, short sleep duration was no longer associated with hypertension. There were similar results in men and women after adjustment for potential confounding factors. Long sleep duration was associated with elevated hypertension prevalence, with long sleepers (≥ 9 hours) having higher odds of hypertension in men (OR = 1.16, 95% CI : 1.04-1.30; $P < 0.05$) and women (OR = 1.19, 95% CI : 1.08-1.32; $P < 0.05$). The observed association between sleep duration and hypertension was slightly attenuated among men compared with women.

For peer review only - http://bmjopen.bmj.com/site/about/guidelines.xhtml

Table 4 Multivariable adjusted odds ratio by logistic regression analyses of sleep duration associated with hypertension

	Sleep duration(hours)				
**Population**	**<6**	**6**	**7**	**8**	**≥9**
Total[a]					
Model1	1.47(1.38-1.56) *	1.19(1.12-1.27) *	1	0.89(0.84-0.94)	0.95(0.89-1.02)
Model2	1.02(0.96-1.09)	0.99(0.93-1.06)	1	1.05(0.99-1.11)	1.19(1.10-1.27) *
Model3	1.02(0.95-1.09)	0.99(0.93-1.06)	1	1.05(0.99-1.11)	1.18(1.10-1.27) *
Model4	0.98(0.91-1.05)	0.98(0.92-1.05)	1	1.05(0.99-1.12)	1.16(1.08-1.24) *
Model5	0.96(0.89-1.04)	0.96(0.90-1.03)	1	1.06(0.99-1.12)	1.17(1.08-1.26) *
Male[b]					
Model1	1.33(1.21-1.46) *	1.11(1.01-1.22) *	1	0.93(0.85-1.01)	1.07(0.96-1.19)
Model2	0.97(0.87-1.07)	0.97(0.88-1.07)	1	1.03(0.94-1.13)	1.19(1.07-1.33) *
Model3	0.96(0.86-1.06)	0.97(0.87-1.07)	1	1.03(0.95-1.13)	1.19(1.07-1.33) *
Model4	0.92(0.82-1.03)	0.95(0.86-1.05)	1	1.04(0.95-1.14)	1.17(1.05-1.31) *
Model5	0.89(0.79-1.00)	0.93(0.84-1.03)	1	1.04(0.95-1.14)	1.16(1.04-1.30) *
Female[b]					
Model1	1.62(1.50-1.75) *	1.25(1.15-1.37) *	1	0.88(0.82-0.95)	0.91(0.84-1.00)
Model2	1.05(0.97-1.15)	1.01(0.92-1.11)	1	1.07(0.98-1.15)	1.20(1.09-1.32) *
Model3	1.05(0.96-1.14)	1.01(0.92-1.11)	1	1.07(0.99-1.16)	1.20(1.09-1.32) *
Model4	1.01(0.91-1.11)	1.00(0.91-1.09)	1	1.07(0.98-1.16)	1.17(1.06-1.29) *
Model5	1.00(0.90-1.11)	0.98(0.89-1.07)	1	1.07(0.99-1.17)	1.19(1.08-1.32) *

Data were analyzed as odds ratios (95% CIs) for the "presence of hypertension" relative to "without hypertension" by logistic regression models using the 7 hours sleep duration as the reference group.

*$p < 0.05$.

a. Model 1 was unadjusted; Model 2 was adjusted for age and sex; Model 3 was adjusted for model 2 + education level, occupation, marital status, annual household income; Model 4 was adjusted for model 3 + tea consumption, smoking status, alcohol intake, MET, sedentary leisure time, fresh fruit consumption, insomnia, sleep snoring; Model 5 was adjusted for model 4 + BMI, WC, DM, stroke and CHD.

b. Model 1 was unadjusted; Model 2 was adjusted for age; Model 3 was adjusted for model 2 + education level, occupation, marital status, annual household income; Model 4 was adjusted for model 3 + tea consumption, smoking status, alcohol intake, MET, sedentary leisure time, fresh fruit consumption, insomnia, sleep snoring; Model 5 was adjusted for model 4 + BMI, WC, DM, stroke and CHD.

The relationship between sleep duration and hypertension Stratification by health-related factors

Results of covariate-adjusted analyses stratified by health-related factors examining relationship between sleep duration and hypertension were displayed in **Figure 1**. Long sleep was associated with elevated hypertension prevalence for 45-59 and 60-79 years old subgroups,

For peer review only

though the relationship was nominally stronger among 45-59 individuals. However, this association was not found among 30-44 years old subgroup. Among those usually at least once a week tea consumers, long sleep was associated with hypertension, and the relationship was not present across never/almost never or only occasionally tea consumers. Among those who were never regular alcohol drinker, ex-regular or usually at least once a week alcohol drinker, long sleep duration was associated with elevated hypertension prevalence. However, among occasional or seasonal alcohol drinker, the relationship with long sleep was not present. Among different smoking status subgroups, long sleep was associated with hypertension among never smoker and daily or almost every day smoker, and this association was not observed among ex-regular and occasional smoker. Long sleep was associated with elevated hypertension prevalence for non-regular fresh fruit consumers, while not among regular fresh fruit consumers. Long sleep was associated with elevated hypertension prevalence for insomnia subgroup, while not among non-insomnia subgroup.

The dose-response relationship between sleep duration and hypertension by restricted cubic splines regression

Results of restricted cubic splines regression to calculate and visualize the relationship of sleep duration with hypertension were displayed in **Figure 2**. The odds of hypertension was relatively flat until around 6.81 hours of sleep duration and then started to increase rapidly afterwards in subjects and we observed a J-shaped pattern (P for nonlinearity = 0.0023). Similar patterns were also observed in male subjects and more than 45 years old subgroups. However, the non-linear tests were not significant for males and subjects more than 60 years old (P for nonlinear > 0.05).There was a U-shaped relationship between sleep duration and hypertension in females (P for nonlinearity = 0.0081). Similar pattern was also observed in 30~44 years old subjects, but the non-linear test was not significant (P for nonlinearity = 0.2481).

For peer review only - http://bmjopen.bmj.com/site/about/guidelines.xhtml

The relationship between sleep duration and SBP and DBP

Age and gender-specific linear regression coefficients of sleep duration for SBP and DBP were presented in **Table 5**. After adjusting for covariates as mentioned in Table 4, sleep duration showed significant and positive associations with SBP and DBP both in females and males. Regression coefficients (SEs) of sleep duration and SBP were 0.474 (0.088) in males and 0.417 (0.070) in females. Regression coefficients (SEs) of sleep duration and DBP were 0.203 (0.051) in males and 0.102 (0.039) in females. Age-specific linear regression coefficients of sleep duration for SBP and DBP were elevated with age. The association patterns were substantially similar for SBP in two genders. However, no significant associations were found between sleep duration and SBP in 30~44 years group among males. Only the association parameters for DBP were significant in 45~59 years old males and 30~44 years old females.

Table 5 Regression coefficients (SEs) of sleep duration associated with SBP and DBP by gender and age groups

	SBP				DBP			
Variables	30~44	45~59	≥60	Total	30~44	45~59	≥60	Total
Male[a]	0.213(0.123)	0.566(0.134) ***	0.554(0.195) ***	0.474(0.088) ***	0.072(0.09)	0.320(0.079) ***	0.194(0.103)	0.203(0.051) ***
Female[a]	0.227(0.091) *	0.378(0.113) ***	0.716(0.178) ***	0.417(0.070) ***	0.139(0.061) *	0.070(0.061)	0.150(0.092)	0.102(0.039) **
Overall[b]	0.219(0.073) ***	0.424(0.087) ***	0.646(0.131) ***	0.408(0.055) ***	0.116(0.050) *	0.153(0.048) **	0.166(0.069) *	0.131(0.031) ***

*p < 0.05, **p < 0.01, **p < 0.001.

a. Age, education level, occupation, marital status, income, tea consumption, smoking status, alcohol intake, MET, sedentary leisure time, fresh fruit consumption, insomnia, sleep snoring, BMI, WC, DM, stroke and CHD were included in models for adjustment.

b. Age, sex, education level, occupation, marital status, income, tea consumption, smoking status, alcohol intake, MET, sedentary leisure time, fresh fruit consumption, insomnia, sleep snoring, BMI, WC, DM, stroke and CHD were included in models for adjustment.

Abbreviations: SBP, systolic blood pressure; DBP, diastolic blood pressure.

For peer review only - http://bmjopen.bmj.com/site/about/guidelines.xhtml

DISCUSSION

This study examined the relationship between sleep duration and hypertension among more than 50 000 rural adults in southwest China. Long sleep duration was associated with hypertension among men, women and the general population, which was consistent with several previous studies in different countries.[16-18 28] For example, the first study assessed the association between sleep duration and hypertension conducted by Gottlieb et. al[17] reported that compared to subjects sleeping 7 to less than 8 hours per night, the adjusted ORs (95% CIs) for hypertension of those sleeping 8 hours and more than 9 hours were 1.19 (1.04-1.37) and 1.30 (1.04-1.62), respectively. Long sleep duration of equal to or more than 8 hours per night was associated with hypertension in participants aged 40–100 years. The Nurse' health study[28] found long sleep duration (≥ 9 hours) was associated with elevated hypertension prevalence in cross-sectional study. A systematic review and meta-analysis conducted by Guo et. al[29] also found that long sleep duration was associated with hypertension (OR = 1.11, 95% CI: 1.04-1.18). Another meta-analysis conducted by Wang et. al[30] indicated that the OR (95% CI) for long sleep duration and hypertension was 1.11 (1.05-1.17).

However, numerous studies showed that compared with normal sleep duration, short sleep duration was more likely to be associated with hypertension than long sleep duration.[9-12 31 32] A cross-sectional study conducted among rural population in China showed that short sleep duration was associated with hypertension in women.[32] Another cross-sectional study in China including 43 655 participants found that short sleep duration of less than 6 hours was related to hypertension in both genders.[10] Results from the Kaiyuan study showed that short sleep duration (≤ 5 hours) was associated with an increased incidence of hypertension during the 3.98 years follow-up period among women and those aged less than 60 years old.[9] A prospective study conducted by Gangwisch et. al reported that short sleep duration (≤ 5 hours) could significantly elavate the risk of hypertension (HR = 2.10; 95% CI, 1.58 to 2.79) in subjects between 32 to 59 years old.[11]

In this study, restricted cubic splines regression was used to visualize the relationship of sleep duration and hypertension. The odds of hypertension was relatively flat until around 6.81

For peer review only - http://bmjopen.bmj.com/site/about/guidelines.xhtml

hours of sleep duration and then started to increase rapidly afterwards and a J-shaped pattern was observed among adults. There was a U-shaped association between sleep duration and hypertension in females. However, inconsistent with many previous studies,[15-17 33] the association between short sleep duration and hypertension was not obvious in this study. There were plausible explanations for the different results of these studies. First, different definition of sleep duration groups and adjustment of different possible influencing factors would lead to differences in statistical analysis. Second, the discrepancy may be due to the difference of study population. Different demographic factors and behavioral habits also would lead to the differences of the relationship between sleep duration and hypertension. Previous studies indicated that both insomnia[34] and snoring[35] could elevated risk of hypertension, and our study adjusted these factors for multivariate analysis. In addition, relationship of sleep duration and blood pressure was also explored in this study. After adjusting for potential confounders, sleep duration showed significant and positive associations with SBP and DBP both in females and males. Regression coefficients of sleep duration and SBP were 0.474 in males and 0.417 in females. Regression coefficients of sleep duration and DBP were 0.203 in males and 0.102 in females. These results revealed that sleep duration was more closely associated with SBP than DBP.

The association between sleep duration and hypertension could reflect a variety of mechanisms. Several studies indicated that potential pathophysiological mechanisms supporting the association between short sleep duration and hypertension in humans may be sleep deprivation. Sleep deprivation has been reported as a cause of over-activity of the sympathetic nervous system, which may elevate blood pressure.[11 36] Sleep deprivation could disrupt circadian rhythmicity, contribute to over-activity of the renin-angiotensin aldosterone system, pro-inflammatory responses, endothelial dysfunction, and renal impairment. Furthermore, sleep deprivation could increase appetite and suppress renal salt fluid excretion, leading to elevated caloric consumption, excessive salt intake and weight gain, which were proved to be risk factors of hypertension.[11 37] However, the mechanisms of long sleep duration with hypertension were even not known. Possible explanations may be as follows. First, some

For peer review only - http://bmjopen.bmj.com/site/about/guidelines.xhtml

other risk factors might impact this association. Long sleepers may have less physical activities,[38] and be related to sleep disorders (sleep apnea, etc.) or poor sleep quality, which could lead to adverse healthy outcomes.[16] In previous studies, long sleep was associated with increased all-cause mortality, high risk of diabetes and other unhealthy outcomes.[38-40] Second, sleep duration may reflect sleep need, which is different in individuals due to genetic, behavioral, and environmental factors.[17] Individuals with increased sleep needs may have a reduced physiologic reserve, reducing their ability to survive serious illness.[38] Third, sleep habits may be a marker of health status rather than a cause for disease, so it is likely that long sleep duration is an epiphenomenon instead of an etiological factor for hypertension.[18 41]

The relationship between sleep duration and hypertension may varied by health-related factors. This study made stratified analyses of sleep duration and hypertension stratified by age, tea consumption, alcohol intake, smoking status, fresh fruit consumption and insomnia. Long sleep were associated with elevated hypertension prevalence among participants aged 45 to 79 years old. However, this association was not found among those aged 30-44 years old. By restricted cubic splines regression, a J-shaped pattern was observed in 45~59 years old subgroups. These findings were different from several previous studies. For example, a Korean study found that short sleep duration was associated with hypertension prevalence only in those aged less than 60 years.[42] A Spanish study demonstrated that sleep duration was not associated with hypertension in older adults.[43] A cross sectional study conducted in northeast China observed an association between short sleep duration and hypertension in young adults aged 18 to 44 years, and the relationship was not found for participants aged 45 to 79 years.[19] Changes in sleep quality and quantity in different stage of life may be related to this age-dependent association.[44] In addition, healthy sleep was influenced by a lot of factors. Besides sleep duration, sleep disorders such as insomnia,[45 46] obstructive sleep apnea[47] and other sleep quality problems[48-50] have also been shown to be risk factors for hypertension. Moreover, the number of younger subjects was larger than that of older subjects, so the lack of an association between long sleep duration and hypertension in the younger subjects could be attribute to a lack of statistical power.

For peer review only - http://bmjopen.bmj.com/site/about/guidelines.xhtml

In this study, long sleep were associated with elevated hypertension prevalence for non-regular fresh fruit consumers, while not among subjects who ate fresh fruit regularly. Fresh fruit intake could reduce the risk and mortality of cardiovascular disease. Results from previous prospective analyses of CKB showed that compared with participants who never or rarely consumed fresh fruit, those who ate fresh fruit daily had lower systolic blood pressure (by 4 mm Hg, P<0.001). The adjusted hazard ratios (95% CIs) for daily consumers were 0.60 (0.54 to 0.67) for cardiovascular death, and 0.66 (0.58 to 0.75), 0.75 (0.72 to 0.79), and 0.64 (0.56 to 0.74) for incident major coronary events, ischemic stroke, and hemorrhagic stroke, respectively.[51] Another prospective study of CKB found that fresh fruit intake reduced all-cause mortality, cardiovascular disease (CVD), cancer and chronic obstructive pulmonary disease mortality.[52] These results may explain the difference of relationship between long sleep duration and hypertension among non-regular and regular fresh fruit consumers. The association between long sleep duration and hypertension was more pronounced in individuals who had insomnia than non-insomnia subgroup. Two factors may explain the differential associations. First, insomnia has been proved to be a risk factor for hypertension.[34] Among insomnia participants, long sleep duration was more likely to be an unusual schedule, which could affect health outcomes in humans. Second, the sample size of non-insomnia subgroup was much higher than that of the insomnia group, which may lead to a statistical difference.

Our study had several strengths. This study was part of CKB, which was a large prospective cohort study in China. CKB had a series of standardized procedures and good quality control measures, so the data was relatively reliable. The sample size was large in this study, which could be a representative rural population in southwest China. Although this study revealed an association between long sleep duration and hypertension, several potential limitations of this study should be noted and need to be addressed in future work. First, our study was a cross-sectional study, so the directionality of the causation could not be established which did not allow for inferences regarding causation to be made. Further prospective cohort studies would be needed to confirm this association. Second, the data of sleep duration was self-reported without more detailed and objective measures of sleep, which might have

For peer review only - http://bmjopen.bmj.com/site/about/guidelines.xhtml

information bias and was less accurate than actigraphy and polysomnography. Third, although we have attempted to adjust for various potential confounders, our results may still be subject to residual confounding from unmeasured or unknown influencing factors.

CONCLUSIONS

In conclusion, this study identified long sleep duration was significantly associated with hypertension and a J-shaped pattern was observed amongadults in southwest China. However, this association was not found between short sleep duration and hypertension. It is important to sustain a proper amount of sleep duration for health. Further prospective studies are required to confirm these findings.

For peer review only

Acknowledgments We thank Chinese Center for Disease Control and Prevention, Chinese Ministry of Health, National Health and Family Planning Commission of China, and 10 provincial/regional Health Administrative Departments. The most important acknowledgement is to the participants in the study and the members of the survey teams in each of the 10 regional centers, as well as to the project development and management teams based at Beijing, Oxford and the 10 regional centers.

Contributors XW and GL conceived and designed the paper. LL and ZC designed and supervised the conduct of the CKB study, obtained funding and together with YG and PP acquired the data. XC, XC and JJ analysed the data. XC, XC, QS and NZ drafted the manuscript. XC, XC, JJ, GL, XC, QS, NZ and XW contributed to the interpretation of the results and critical revision of the manuscript. All authors contributed to and approved the final manuscript. XW is the study guarantor.

Funding This work was supported by grants ("Tianfu ten thousand talents plan" fund in 2018) from Sichuan Provincial Leading Group Office for Talent Work, grants (2016YFC0900500, 2016YFC0900501, 2016YFC0900504) from the National Key Research and Development Program of China, grants from the Kadoorie Charitable Foundation in Hong Kong (N/A), grants (212946/Z/18/Z, 202922/Z/16/Z, 104085/Z/14/Z, 088158/Z/09/Z) from Wellcome Trust in the UK. The funders had no role in the study design, data collection, data analysis and interpretation, writing of the report or the decision to submit the article for publication.

Competing interests None declared.

Patient consent for publication Not required.

Ethics approval The Ethical Review Committee of the Chinese Center for Disease Control and Prevention (Beijing, China) (Ethics ID number of approval: 005/2004) and the Oxford Tropical Research Ethics Committee, University of Oxford (UK) (Ethics ID number of approval: OXTREC 025-04).

Provenance and peer review Not commissioned; externally peer reviewed.

Data availability statement The dataset supporting the conclusions of this article is available from the study website (http://www.ckbiobank.org), along with the access policy and

For peer review only

procedures.

REFERENCES

1 Organization WH. A global brief on hypertension : silent killer, global public health crisis: World Health Day 2013. In. Geneva: World Health Organization; 2013.

2 Wang Y, Peng X, Nie X*, et al.* Burden of hypertension in China over the past decades: Systematic analysis of prevalence, treatment and control of hypertension. *Eur J Prev Cardiol* 2016; 23:792-800.

3 Wang Z, Chen Z, Zhang L*, et al.* Status of Hypertension in China: Results From the China Hypertension Survey, 2012-2015. *Circulation* 2018; 137:2344-56.

4 Zhou M, Wang H, Zeng X*, et al.* Mortality, morbidity, and risk factors in China and its provinces, 1990-2017: a systematic analysis for the Global Burden of Disease Study 2017. *Lancet* 2019; 394:1145-58.

5 Cao D, Zhou Z, Si Y*, et al.* Prevalence and income-related equity in hypertension in rural China from 1991 to 2011: differences between self-reported and tested measures. *BMC Health Serv Res* 2019; 19:437.

6 Liu X, Zhang D, Liu Y*, et al.* Dose-Response Association Between Physical Activity and Incident Hypertension: A Systematic Review and Meta-Analysis of Cohort Studies. *Hypertension* 2017; 69:813-20.

7 Forman JP, Stampfer MJ, Curhan GC. Diet and lifestyle risk factors associated with incident hypertension in women. *Jama* 2009; 302:401-11.

8 Li H, Ren Y, Wu Y*, et al.* Correlation between sleep duration and hypertension: a dose-response meta-analysis. *J Hum Hypertens* 2019; 33:218-28.

9 Song Q, Liu X, Wang X*, et al.* Age- and gender-specific associations between sleep duration and incident hypertension in a Chinese population: the Kailuan study. *J Hum Hypertens* 2016; 30:503-7.

10 Guan Y, Zhang M, Zhang X*, et al.* Association between sleep duration and hypertension of migrant workers in China: a national cross-sectional surveillance study. *BMJ Open* 2019; 9:e031126.

11 Gangwisch JE, Heymsfield SB, Boden-Albala B*, et al.* Short sleep duration as a risk factor for hypertension: analyses of the first National Health and Nutrition Examination Survey. *Hypertension* 2006; 47:833-9.

12 Cappuccio FP, Stranges S, Kandala NB*, et al.* Gender-specific associations of short sleep duration with prevalent and incident hypertension: the Whitehall II Study. *Hypertension* 2007; 50:693-700.

13 Kim J, Jo I. Age-dependent association between sleep duration and hypertension in the adult Korean population. *Am J Hypertens* 2010; 23:1286-91.

14 Knutson KL, Van Cauter E, Rathouz PJ*, et al.* Association between sleep and blood pressure in midlife: the CARDIA sleep study. *Arch Intern Med* 2009; 169:1055-61.

15 Grandner M, Mullington JM, Hashmi SD*, et al.* Sleep Duration and Hypertension: Analysis of > 700,000 Adults by Age and Sex. *J Clin Sleep Med* 2018; 14:1031-39.

16 Fang J, Wheaton AG, Keenan NL*, et al.* Association of sleep duration and hypertension among US adults varies by age and sex. *Am J Hypertens* 2012; 25:335-41.

17 Gottlieb DJ, Redline S, Nieto FJ*, et al.* Association of usual sleep duration with hypertension: the Sleep Heart Health Study. *Sleep* 2006; 29:1009-14.

18 Magee CA, Kritharides L, Attia J*, et al.* Short and long sleep duration are associated with prevalent cardiovascular disease in Australian adults. *J Sleep Res* 2012; 21:441-7.

For peer review only - http://bmjopen.bmj.com/site/about/guidelines.xhtml

19 Li M, Yan S, Jiang S*, et al.* Relationship between sleep duration and hypertension in northeast China: a cross-sectional study. *BMJ Open* 2019; 9:e023916.

20 Chen Z, Lee L, Chen J*, et al.* Cohort profile: the Kadoorie Study of Chronic Disease in China (KSCDC). *Int J Epidemiol* 2005; 34:1243-9.

21 Chen Z, Chen J Fau - Collins R, Collins R Fau - Guo Y*, et al.* China Kadoorie Biobank of 0.5 million people: survey methods, baseline characteristics and long-term follow-up.

22 Chen X, Du H, Zhang J*, et al.* Adiposity and blood pressure among 55 000 relatively lean rural adults in southwest of China. *J Hum Hypertens* 2015; 29:522-9.

23 Lima-Costa MF, Peixoto SV, Rocha FL. Usual sleep duration is not associated with hypertension in Brazilian elderly: The Bambui Health Aging Study (BHAS). *Sleep Med* 2008; 9:806-7.

24 Zhou BF. Predictive Values of Body Mass Index and Waist Circumference for Risk Factors of Certain Related Diseases in Chinese Adults - Study on Optimal Cut-off Points of Body Mass Index and Waist Circumference in Chinese Adults. *Biomedical & Environmental Sciences* 2002; 11:S685-S93.

25 Tian X, Du H. Fruit consumption and physical activity in relation to all-cause and cardiovascular mortality among 70,000 Chinese adults with pre-existing vascular disease. PLoS One 2017; 12:e0173054..

26 Du H, Bennett D, Li L*, et al.* Physical activity and sedentary leisure time and their associations with BMI, waist circumference, and percentage body fat in 0.5 million adults: the China Kadoorie Biobank study. *Am J Clin Nutr* 2013; 97:487-96.

27 Zheng B, Yu C, Lin L*, et al.* Associations of domain-specific physical activities with insomnia symptoms among 0.5 million Chinese adults. *J Sleep Res* 2017; 26:330-37.

28 Gangwisch JE, Feskanich D, Malaspina D*, et al.* Sleep duration and risk for hypertension in women: results from the nurses' health study. *Am J Hypertens* 2013; 26:903-11.

29 Guo X, Zheng L, Wang J*, et al.* Epidemiological evidence for the link between sleep duration and high blood pressure: a systematic review and meta-analysis. *Sleep Med* 2013; 14:324-32.

30 Wang Q, Xi B, Liu M*, et al.* Short sleep duration is associated with hypertension risk among adults: a systematic review and meta-analysis. *Hypertens Res* 2012; 35:1012-8.

31 Makarem N, Shechter A, Carnethon MR*, et al.* Sleep Duration and Blood Pressure: Recent Advances and Future Directions. *Curr Hypertens Rep* 2019; 21:33.

32 Wang H, Zee P, Reid K*, et al.* Gender-specific association of sleep duration with blood pressure in rural Chinese adults. *Sleep Med* 2011; 12:693-9.

33 Kuciene R, Dulskiene V. Associations of short sleep duration with prehypertension and hypertension among Lithuanian children and adolescents: a cross-sectional study. *BMC Public Health* 2014; 14:255.

34 Jarrin DC, Alvaro PK, Bouchard MA*, et al.* Insomnia and hypertension: A systematic review. *Sleep Med Rev* 2018; 41:3-38.

35 Khazaie H, Negahban S, Ghadami MR, et al. Among middle-aged adults, snoring predicted hypertension independently of sleep apnoea. J Int Med Res 2018; 46:1187-96.

36 Lusardi P, Zoppi A, Preti P*, et al.* Effects of insufficient sleep on blood pressure in hypertensive patients: a 24-h study. *Am J Hypertens* 1999; 12:63-8.

37 Spiegel K, Tasali E, Penev P*, et al.* Brief communication: Sleep curtailment in healthy young men is associated with decreased leptin levels, elevated ghrelin levels, and increased hunger and appetite. *Ann Intern Med* 2004; 141:846-50.

For peer review only - http://bmjopen.bmj.com/site/about/guidelines.xhtml

38 Ayas NT, White DP, Al-Delaimy WK, *et al.* A prospective study of self-reported sleep duration and incident diabetes in women. *Diabetes Care* 2003; 26:380-4.

39 Ayas NT, White DP, Manson JE, *et al.* A prospective study of sleep duration and coronary heart disease in women. *Arch Intern Med* 2003; 163:205-9.

40 Suzuki E, Yorifuji T, Ueshima K, *et al.* Sleep duration, sleep quality and cardiovascular disease mortality among the elderly: a population-based cohort study. *Prev Med* 2009; 49:135-41.

41 Cappuccio FP, Cooper D, D'Elia L, *et al.* Sleep duration predicts cardiovascular outcomes: a systematic review and meta-analysis of prospective studies. *Eur Heart J* 2011; 32:1484-92.

42 Choi KM, Lee JS, Park HS, *et al.* Relationship between sleep duration and the metabolic syndrome: Korean National Health and Nutrition Survey 2001. *Int J Obes (Lond)* 2008; 32:1091-7.

43 Lopez-Garcia E, Faubel R, Guallar-Castillon P, *et al.* Self-reported sleep duration and hypertension in older Spanish adults. *J Am Geriatr Soc* 2009; 57:663-8.

44 Crowley K. Sleep and sleep disorders in older adults. *Neuropsychol Rev* 2011; 21:41-53.

45 Fernandez-Mendoza J, Vgontzas AN, Liao D, *et al.* Insomnia with objective short sleep duration and incident hypertension: the Penn State Cohort. *Hypertension* 2012; 60:929-35.

46 Budhiraja R, Roth T, Hudgel DW, *et al.* Prevalence and polysomnographic correlates of insomnia comorbid with medical disorders. *Sleep* 2011; 34:859-67.

47 Floras JS. Hypertension and Sleep Apnea. *Can J Cardiol* 2015; 31:889-97.

48 Kara B, Tenekeci EG. Sleep Quality and Associated Factors in Older Turkish Adults With Hypertension: A Pilot Study. *J Transcult Nurs* 2017; 28:296-305.

49 Liu RQ, Qian Z, Trevathan E, *et al.* Poor sleep quality associated with high risk of hypertension and elevated blood pressure in China: results from a large population-based study. *Hypertens Res* 2016; 39:54-9.

50 Fiorentini A, Valente R, Perciaccante A, *et al.* Sleep's quality disorders in patients with hypertension and type 2 diabetes mellitus. *Int J Cardiol* 2007; 114:E50-2.

51 Du H, Li L, Bennett D, *et al.* Fresh Fruit Consumption and Major Cardiovascular Disease in China. *N Engl J Med* 2016; 374:1332-43.

52 Du H, Li L, Bennett D, *et al.* Fresh fruit consumption and all-cause and cause-specific mortality: findings from the China Kadoorie Biobank. *Int J Epidemiol* 2017; 46:1444-55.

Figure 1 Adjusted relationships between sleep duration and hypertension stratified by health-related factors. ORs in each group are presented as points with 95% CIs represented by extended lines. The horizontal reference dashed line represents an OR value of '1'. The full line represents the effects of different sleep durations on hypertension. A, 30-44 years old subgroup; B, 45-59 years old subgroup; C, 60-79 years old subgroup; D, Never or almost never tea consumers; E, Only occasionally tea consumers; F, Usually at least once a week tea consumers; G, Never regular alcohol drinker; H, Ex-regular alcohol drinker; I, Occasional or seasonal alcohol drinker; J, Usually at least once a week alcohol drinker; K, Never smoker; L, Ex-regular smoker; M, Occasional smoker; N, Smoked daily or almost every day; O, Non-regular fresh fruit consumers; P, regular fresh fruit consumers; Q, insomnia subgroup; R, non-insomnia subgroup. Multivariable logistic model adjusted age, sex, education level, occupation, marital status, income, tea, smoking status, alcohol intake, MET, sedentary leisure time, fresh fruit consumption, insomnia, sleep snoring, BMI, WC, DM, stroke and CHD.

For peer review only - http://bmjopen.bmj.com/site/about/guidelines.xhtml

Figure 2 Adjusted relationships between sleep duration and hypertension by using restricted cubic splines regression in different gender and age subgroups, adjusted by age, sex, education level, occupation, marital status, income, tea consumption, smoking status, alcohol intake, MET, sedentary leisure time, fresh fruit consumption, insomnia, sleep snoring, BMI, WC, DM, stroke and CHD. ORs are presented as full line with 95% CIs represented by gray area. The horizontal reference dashed line represents an OR value of '1'. The vertical dashed line represents the sleep duration where OR equals 1.

For peer review only

For peer review only - http://bmjopen.bmj.com/site/about/guidelines.xhtml

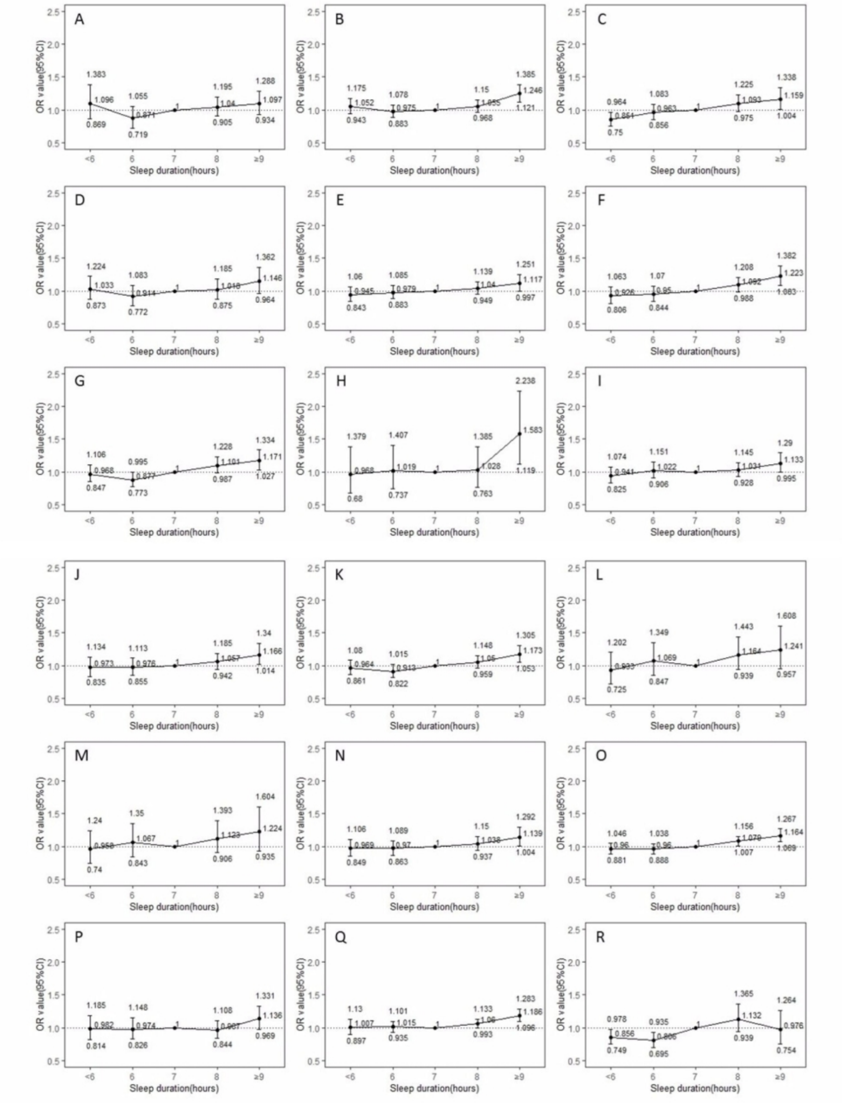

Figure 1 Adjusted relationships between sleep duration and hypertension stratified by health-related factors. ORs in each group are presented as points with 95% CIs represented by extended lines. The horizontal reference dashed line represents an OR value of ‘1’. The full line represents the effects of different sleep durations on hypertension. A, 30-44 years old subgroup; B, 45-59 years old subgroup; C, 60-79 years old subgroup; D, Never or almost never tea consumers; E, Only occasionally tea consumers; F, Usually at least once a week tea consumers; G, Never regular alcohol drinker; H, Ex-regular alcohol drinker; I, Occasional or seasonal alcohol drinker; J, Usually at least once a week alcohol drinker; K, Never smoker; L, Ex-regular smoker; M, Occasional smoker; N, Smoked daily or almost every day; O, Non-regular fresh fruit consumers; P, regular fresh fruit consumers; Q, insomnia subgroup; R, non-insomnia subgroup. Multivariable logistic model adjusted age, sex, education level, occupation, marital status, income, tea, smoking status, alcohol intake, MET, sedentary leisure time, fresh fruit consumption, insomnia, sleep snoring, BMI, WC, DM, stroke and CHD.

118x157mm (300 x 300 DPI)

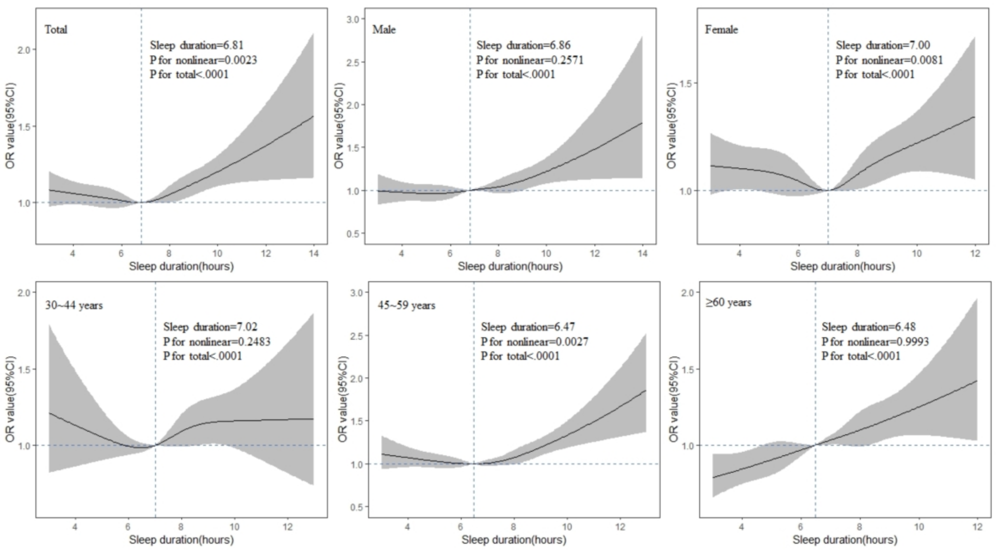

Figure 2 Adjusted relationships between sleep duration and hypertension by using restricted cubic splines regression in different gender and age subgroups, adjusted by age, sex, education level, occupation, marital status, income, tea consumption, smoking status, alcohol intake, MET, sedentary leisure time, fresh fruit consumption, insomnia, sleep snoring, BMI, WC, DM, stroke and CHD. ORs are presented as full line with 95% CIs represented by gray area. The horizontal reference dashed line represents an OR value of '1'. The vertical dashed line represents the sleep duration where OR equals 1.

472x265mm (300 x 300 DPI)

STROBE Statement—Checklist of items that should be included in reports of ***cross-sectional studies***

	Item No	Recommendation	Page No
**Title and abstract**	1	(*a*) Indicate the study's design with a commonly used term in the title or the abstract	1
		(*b*) Provide in the abstract an informative and balanced summary of what was done and what was found	2-3
**Introduction**			
Background/rationale	2	Explain the scientific background and rationale for the investigation being reported	4
Objectives	3	State specific objectives, including any prespecified hypotheses	4
**Methods**			
Study design	4	Present key elements of study design early in the paper	5
Setting	5	Describe the setting, locations, and relevant dates, including periods of recruitment, exposure, follow-up, and data collection	5
Participants	6	(*a*) Give the eligibility criteria, and the sources and methods of selection of participants	5
Variables	7	Clearly define all outcomes, exposures, predictors, potential confounders, and effect modifiers. Give diagnostic criteria, if applicable	5-7
Data sources/ measurement	8*	For each variable of interest, give sources of data and details of methods of assessment (measurement). Describe comparability of assessment methods if there is more than one group	5-7
Bias	9	Describe any efforts to address potential sources of bias	5-7
Study size	10	Explain how the study size was arrived at	5
Quantitative variables	11	Explain how quantitative variables were handled in the analyses. If applicable, describe which groupings were chosen and why	5-7
Statistical methods	12	(*a*) Describe all statistical methods, including those used to control for confounding	7-8
		(*b*) Describe any methods used to examine subgroups and interactions	7-8
		(*c*) Explain how missing data were addressed	None
		(*d*) If applicable, describe analytical methods taking account of sampling strategy	5-8
		(*e*) Describe any sensitivity analyses	6-8
**Results**			
Participants	13*	(a) Report numbers of individuals at each stage of study—eg numbers potentially eligible, examined for eligibility, confirmed eligible, included in the study, completing follow-up, and analysed	9-11
		(b) Give reasons for non-participation at each stage	None
		(c) Consider use of a flow diagram	None
Descriptive data	14*	(a) Give characteristics of study participants (eg demographic, clinical, social) and information on exposures and potential confounders	9-12
		(b) Indicate number of participants with missing data for each variable of interest	9-12
Outcome data	15*	Report numbers of outcome events or summary measures	12-21

For peer review only

For peer review only - http://bmjopen.bmj.com/site/about/guidelines.xhtml

Main results	16	(*a*) Give unadjusted estimates and, if applicable, confounder-adjusted estimates and their precision (eg, 95% confidence interval). Make clear which confounders were adjusted for and why they were included	12-21
		(*b*) Report category boundaries when continuous variables were categorized	12-21
		(*c*) If relevant, consider translating estimates of relative risk into absolute risk for a meaningful time period	12-21
Other analyses	17	Report other analyses done—eg analyses of subgroups and interactions, and sensitivity analyses	19-21
**Discussion**			
Key results	18	Summarise key results with reference to study objectives	22-26
Limitations	19	Discuss limitations of the study, taking into account sources of potential bias or imprecision. Discuss both direction and magnitude of any potential bias	25-26
Interpretation	20	Give a cautious overall interpretation of results considering objectives, limitations, multiplicity of analyses, results from similar studies, and other relevant evidence	22-26
Generalisability	21	Discuss the generalisability (external validity) of the study results	22-26
**Other information**			
Funding	22	Give the source of funding and the role of the funders for the present study and, if applicable, for the original study on which the present article is based	27

*Give information separately for exposed and unexposed groups.

Note: An Explanation and Elaboration article discusses each checklist item and gives methodological background and published examples of transparent reporting. The STROBE checklist is best used in conjunction with this article (freely available on the Web sites of PLoS Medicine at http://www.plosmedicine.org/, Annals of Internal Medicine at http://www.annals.org/, and Epidemiology at http://www.epidem.com/). Information on the STROBE Initiative is available at www.strobe-statement.org.

For peer review only

BMJ Open

The association between sleep duration and hypertension in southwest China: a population-based cross-sectional study

Journal:	*BMJ Open*
Manuscript ID	bmjopen-2021-052193.R2
Article Type:	Original research
Date Submitted by the Author:	21-Feb-2022
Complete List of Authors:	Chang, Xiaoyu; Sichuan Center for Disease Control and Prevention, Department of Chronic and Non-communicable Disease Control and Prevention<br>Chen, Xiaofang; Chengdu Medical College, Department of Epidemiology and Statistics<br>Ji, John; Tsinghua University, Vanke School of Public Health; Duke Kunshan University, Global Health Research Center<br>Luo, Guojin; Pengzhou Center For Disease Control And Prevention<br>Chen, Xiaofang; Pengzhou Center For Disease Control And Prevention<br>Sun, Qiang; Pengzhou Center For Disease Control And Prevention<br>Zhang, Ningmei; Sichuan Center for Disease Control and Prevention<br>Guo, Yu; Chinese Academy of Medical Sciences<br>Pei, Pei; Chinese Academy of Medical Sciences<br>Li, Liming; Peking University, Department of Epidemiology & Biostatistics, School of Public Health; Peking University, Peking University Center for Public Health and Epidemic Preparedness & Response<br>Chen, Zhengming; University of Oxford, Clinical Trial Service Unit and Epidemiological Studies Unit (CTSU), Nuffield Department of Population Health<br>Wu, Xianping ; Sichuan Center for Disease Control and Prevention
**Primary Subject Heading**:	Epidemiology
Secondary Subject Heading:	Public health
Keywords:	Hypertension < CARDIOLOGY, SLEEP MEDICINE, EPIDEMIOLOGY, PUBLIC HEALTH

SCHOLARONE™ Manuscripts

I, the Submitting Author has the right to grant and does grant on behalf of all authors of the Work (as defined in the below author licence), an exclusive licence and/or a non-exclusive licence for contributions from authors who are: i) UK Crown employees; ii) where BMJ has agreed a CC-BY licence shall apply, and/or iii) in accordance with the terms applicable for US Federal Government officers or employees acting as part of their official duties; on a worldwide, perpetual, irrevocable, royalty-free basis to BMJ Publishing Group Ltd ("BMJ") its licensees and where the relevant Journal is co-owned by BMJ to the co-owners of the Journal, to publish the Work in this journal and any other BMJ products and to exploit all rights, as set out in our licence.

The Submitting Author accepts and understands that any supply made under these terms is made by BMJ to the Submitting Author unless you are acting as an employee on behalf of your employer or a postgraduate student of an affiliated institution which is paying any applicable article publishing charge ("APC") for Open Access articles. Where the Submitting Author wishes to make the Work available on an Open Access basis (and intends to pay the relevant APC), the terms of reuse of such Open Access shall be governed by a Creative Commons licence – details of these licences and which Creative Commons licence will apply to this Work are set out in our licence referred to above.

Other than as permitted in any relevant BMJ Author's Self Archiving Policies, I confirm this Work has not been accepted for publication elsewhere, is not being considered for publication elsewhere and does not duplicate material already published. I confirm all authors consent to publication of this Work and authorise the granting of this licence.

For peer review only

The association between sleep duration and hypertension in southwest China: a population-based cross-sectional study

Xiaoyu Chang,[1] Xiaofang Chen,[2] John S Ji,[3 4] Guojin Luo,[5] Xiaofang Chen,[5] Qiang Sun,[5] Ningmei Zhang,[1] Yu Guo,[6] Pei Pei,[6] Liming Li,[7] Zhengming Chen,[8] Xianping Wu[1]

Correspondence to Professor Xianping Wu; wwwuxp@163.com

Name: Xianping Wu; Postal address: No. 6 Zhongxue Road, Wuhou District, Chengdu, Sichuan Province, China (Postal Code: 610041); E-mail: wwwuxp@163.com; Telephone: 86-28-85589582; Fax numbers: 86-28-85582320.

Author affiliations

[1] Department of Chronic and Non-communicable Disease Control and Prevention, Sichuan Center for Disease Control and Prevention, Chengdu, China

[2] Department of Epidemiology and Statistics, Chengdu Medical College, Chengdu, China

[3] Vanke School of Public Health, Tsinghua University, Beijing, China.

[4] Global Health Research Center, Duke Kunshan University, Kunshan, China

[5] Pengzhou Center for Disease Control and Prevention, Pengzhou, China

[6] Chinese Academy of Medical Sciences, Beijing, China

[7] Department of Epidemiology & Biostatistics, School of Public Health, Peking University Health Science Center , Beijing, China; Peking University Center for Public Health and Epidemic Preparedness & Response, Beijing, China

[8] Clinical Trial Service Unit and Epidemiological Studies Unit (CTSU), Nuffield Department of Population Health, University of Oxford, Oxford, UK

Word count: 5027

For peer review only - http://bmjopen.bmj.com/site/about/guidelines.xhtml

ABSTRACT

Objective Hypertension is a major risk factor and cause of many non-communicable diseases in China. While there have been studies on various diet and lifestyle risk factors, we do not know whether sleep duration has an association to blood pressure in southwest China. This predictor is useful in low-resource rural settings. We examined the association between sleep duration and hypertension in southwest China.

Design Population-based cross-sectional study

Setting This study was part of the baseline survey of a large ongoing prospective cohort study, the China Kadoorie Biobank. Participants were enrolled in 15 townships of Pengzhou city in Sichuan province during 2004-2008.

Participants 55 687 participants aged 30 to 79 years were included. Sleep duration was assessed by a self-reported questionnaire.

Main outcome measures Hypertension was defined as systolic blood pressure ≥ 140 mm Hg and/or diastolic blood pressure ≥ 90 mm Hg, or prior physician-diagnosed hypertension in hospitals at the township (community) level or above.

Results The prevalence of hypertension was 25.17%. The percentages of subjects with sleep durations of <6, 6, 7, 8 and ≥ 9 hours were 17.20%, 16.14%, 20.04%, 31.95% and 14.68%, respectively. In multivariable adjusted analyses, the increased odds ratios (95% CIs) of having hypertension was across those who reported ≥ 9 hours of sleep (men: 1.16, 1.04 -1.30; women: 1.19, 1.08 -1.32; general population: 1.17, 1.08 -1.26). The odds of hypertension was relatively flat until around 6.81 hours of sleep duration and then started to increase rapidly afterwards in subjects and a J-shaped pattern was observed. There was a U-shaped relationship between sleep duration and hypertension in females.

Conclusion Long sleep duration was significantly associated with hypertension and a J-shaped pattern was observed among rural adults in southwest China, independent of potential confounders. However, this association was not obvious between short sleep duration and hypertension.

Strengths and limitations of this study

For peer review only - http://bmjopen.bmj.com/site/about/guidelines.xhtml

This study examined the association between sleep duration and hypertension in southwest China, and this predictor was useful in low-resource rural settings.

A large sample of rural population was obtained, which could represent most of the rural places in southwest China.

A broad range of covariates were controlled in the analysis, including age, sex, education, occupation, marital status, income, tea, smoking, alcohol, MET, sedentary leisure time, fresh fruit consumption, insomnia, sleep snoring, BMI, WC, DM, stroke and CHD.

The dose-response relationship between sleep duration and hypertension was explored and visualized by restricted cubic splines regression.

The sleep duration was self-reported without more detailed and objective measures of sleep, which was less accurate than actigraphy and polysomnography.

For peer review only - http://bmjopen.bmj.com/site/about/guidelines.xhtml

INTRODUCTION

Hypertension is a condition in which the blood vessels have persistently raised pressure.[1] It is the leading risk factor for global cardiovascular disease burden and mortality.[2] In China, hypertension is also a serious public health problem. The China hypertension survey showed that the prevalence of hypertension and pre-hypertension were 23.2% and 41.3% among Chinese adults, which means that there is one hypertensive patient per four people.[3] High systolic blood pressure was the leading risk factors contributing to deaths and DALYs.[4] In addition, hypertension is a growing problem not only in urban but also in rural China. The prevalence of hypertension has sharply increased in rural China,[5] which is similar in rural and urban settings (23.4% versus 23.1%).[3] Disease burden of hypertension is increasing globally, especially as places of poverty become more prosperous.[2]

Previous studies about influencing factors of hypertension mainly focus on diet (high dietary sodium intake), exercise and other lifestyle factors,[6 7] while some studies in recent years documented the relationship between sleep and hypertension. Sleep status influence blood pressure by altering autonomic nervous system function and other physiologic events. Unhealthy sleep status such as sleep apnea, insomnia, long and short sleep duration can increase risk of hypertension. Even though a variety of studies examined sleep duration to be associated with hypertension, the results were not completely consistent. Some studies showed that short sleep duration increased the risk of hypertension,[8-14] others showed that long sleep duration was related to hypertension or the association were J- or U-shaped curves.[15-18] However, most of the study populations were from western countries. A small number of studies were conducted in Asian countries,[9 10 13 19] and there was limited information about the association between sleep duration and hypertension in southwest China. Therefore, the aim of this study is to analyze the baseline data from a large prospective cohort study of the China Kadoorie Biobank to explore the association of sleep duration with hypertension among more than 50 000 adults in southwest China.

For peer review only - http://bmjopen.bmj.com/site/about/guidelines.xhtml

SUBJECTS AND METHODS

Study population

This study utilizes a large, ongoing prospective cohort study, the China Kadoorie Biobank (CKB). The baseline survey of CKB was conducted in five urban and five rural areas across China from 2004 to 2008. Our data was derived from the baseline survey of CKB in Sichuan province. A total of 55 687 residents aged 30 to 79 years in 15 townships of Pengzhou city in Sichuan province had completed baseline information collection, including a questionnaire, anthropometric measurements, blood sample collection, and a written informed consent. More details about the study design are available in previous publications.[20-22]

Data collection

The baseline survey took place in temporary study clinics, specially set up near participants' residential areas. Information on sociodemographic characteristics (age, sex, education level, occupation, marital status, annual household income), behavioural risk factors for chronic diseases (diet, tea consumption, smoking status, alcohol intake, physical activity, sedentary leisure time, sleep), and medical history were collected through an interviewer-administered computerized questionnaire by face-to-face interview with trained interviewers according to standard protocols and procedures. A range of physical measurements were also undertaken, including height, weight, waist circumference and so on.

Outcome measurement ascertainment

Participants were asked to report the number of hours they slept a day during the last 12 months. Sleep duration was assessed by a self-reported questionnaire with the following question: "On average, how many hours do you typically sleep per day (including daytime naps)?". Respondents could report in only 1-hour increments. To maintain consistency with previous literatures,[10 17 18 23] sleep duration was categorized as five groups of <6, 6, 7 (which regarded as the reference group for our analysis), 8 and ≥ 9 hours.

As described in the previous study,[22] blood pressure was measured twice using a UA-779

For peer review only - http://bmjopen.bmj.com/site/about/guidelines.xhtml

digital monitor recommended by the British Hypertension Society (A & D Instruments Ltd., Abingdon, UK). If the systolic blood pressure (SBP) difference between the first two measurements was more than 10 mm Hg, a third measurement was obtained and the last two measurements were recorded. The mean of two blood pressure measurements was used for this study. Hypertension was defined as systolic blood pressure (SBP) ≥ 140 mm Hg and/or diastolic blood pressure (DBP) ≥ 90 mm Hg, or prior physician-diagnosed hypertension in hospitals at the township (community) level or above.

Several variables were used both as covariates in adjusted models and as variables for stratification. Sociodemographic characteristics included age at baseline survey (30-79 years old), sex (male or female), education level (no formal school, primary school, middle school, high school and above), occupation (famer or others), marital status (married or never married/widowed/separated/divorced), annual household income (< 5 000, 5 000~9 999, ≥10 000, RMB, yuan). Behavioural risk factors included tea consumption (never or almost never, only occasionally, usually at least once a week), smoking status (never, ex-regular, occasionally, daily or almost every day), alcohol intake (never regular, ex-regular, occasionally or seasonally, usually at least once a week), fresh fruit consumption (daily, 4-6 days per week, 1-3 days per week , monthly, never/rarely), physical activity, sedentary leisure time (hours/week), insomnia symptoms (yes or no), sleep snoring (never, occasionally, usually) , waist circumference (WC) and body mass index (BMI). Medical history included diabetes mellitus (DM), stroke and coronary heart disease (CHD). BMI was calculated as weight in kilograms (kg) / height in metres $(m)^2$. According to the Guidelines for Prevention and Control of Overweight and Obesity in Adults in China,[24] BMI was divided into four groups: underweight (BMI <18.5 kg/m^2), normal weight (18.5 ≤ BMI <24 kg/m^2), overweight (24 ≤ BMI <28 kg/m^2) and obesity (BMI ≥28 kg/m^2). Waist circumference beyond 85 cm for men and 80 cm for women were recommended as central obesity.

Information about type, frequency, and duration of occupational, commuting-related, household and active recreational (leisure-time) physical activities were used to calculate total physical activity in metabolic equivalents (MET, hours/day).[25 26] Sedentary leisure time

For peer review only - http://bmjopen.bmj.com/site/about/guidelines.xhtml

(hours/week) was assessed by the question of "About how many hours per week did you watch TV or read?". Insomnia symptoms were determined by three questions: Taking >30 minutes to fall asleep after going to bed or waking up in the middle of the night; Waking up early and not being able to go back to sleep; Having difficulty staying alert while at work, eating or meeting people during daytime. Those reporting "Yes" for one or more of the three questions were classified as having insomnia symptoms.[27] Participants consumed fresh fruit more than 3 days/week were defined as regular consumers, otherwise, those were never or occasionally consumers.[25]

Statistical Analysis

Continuous variables were expressed as mean and standard deviation (SD). Frequency and percentage distributions were reported for categorical variables. Continuous variables included age, MET, sedentary leisure time, BMI, WC, SBP, DBP. Categorical variables included age groups, gender, education level, occupation, marital status, income, tea consumption, smoking status, alcohol intake, fresh fruit consumption, insomnia, sleep snoring, BMI groups, DM, stroke and CHD. Prevalence of hypertension was calculated for the overall population and different subgroups, including sleep duration and some potential confounding factors. Chi-square tests were conducted to test for group differences in prevalence or percentage, and the Cochran-Armitage tests for trend were used to examine the trends for ordered categorical variables. After examining normality of the continuous covariates, the analysis of variance was used for continuous variables of study participants by different sleep durations. The statistical significance of associations for the continuous and categorical variables with sleep duration was estimated. Multivariable logistic regression models were used to calculate the odds ratios (ORs) and 95% confidence intervals (CIs) for hypertension between sleep duration subgroups adjusted for potential confounders, including age, gender, education level, occupation, marital status, income, tea consumption, smoking status, alcohol intake, MET, sedentary leisure time, fresh fruit consumption, insomnia, sleep snoring, BMI, WC, DM, stroke and CHD. In addition, we performed subgroup analysis stratified by health-related factors, such as age, sex, tea

For peer review only - http://bmjopen.bmj.com/site/about/guidelines.xhtml

consumption, smoking status, alcohol intake, insomnia. Potential nonlinear association between sleep duration and hypertension was examined with restricted cubic splines regression. We used restricted cubic splines regression with five knots at the 5th, 35th, 50th, 65th, and 95th centiles to flexibly model the association of sleep duration and hypertension.[28-30] Multiple linear regression model was used to explore the relationship for sleep duration with SBP and DBP. A two-tailed *P* value of less than 0.05 was considered statistically significant. All data processing and statistical analyses were performed using Statistical Analysis Software, version 9.4 (SAS Institute, Cary, NC) and R version 4.0.3 (R Foundation for Statistical Computing, Vienna, Austria).

Patient and public involvement

No patients were involved in the present study, the interpretation of study results or write-up of the manuscript. The results were presented to study participants by the website of the CKB (http://www.ckbiobank.org/site/) and newsletters.

For peer review only - http://bmjopen.bmj.com/site/about/guidelines.xhtml

RESULTS

Different demographic characteristics of study population across sleep duration groups

The baseline characteristics of study population for subgroups according to sleep duration were presented in **Table 1**. A total of 55 687 adults completed baseline information, including 34 372 women and 21 315 men. The mean (±SD) age of study population was 51.03±10.54 years (range: 30–79 years). The mean (±SD) sleep duration was 7.05±1.68 hours, which were 7.07±1.61 hours in men and 7.04±1.72 hours in women. The overall means (±SD) of SBP and DBP were 129.24±19.01 mm Hg and 77.23 ± 10.23 mm Hg, respectively. The percentages of the subjects with sleep durations of <6, 6, 7, 8 and ≥ 9 hours were 17.20%, 16.14%, 20.04%, 31.95% and 14.68%, respectively.

Individuals with less hours of sleep per day were more likely to be female, eldly, low education level, famer, single, low annual household income, never or almost never tea consumers, low frequency of fresh fruit consumption, low BMI. Short sleepers were more likely to be DM, CHD, stroke or hypertensive subjects. With the increase of age, the proportion of subjects who slept for equal to or more than 8 hours decreased, while the proportion of subjects who slept for equal to or less than 6 hours increased. With the increase of education level, the proportion of subjects with less than 6 hours of sleep duration decreased. With the increase of annual household income and tea consumption, the proportion of subjects who slept for less than 6 hours decreased. With the increase of BMI and fresh fruit consumption, the proportion of subjects who slept for less than 6 hours decreased, while the proportion of subjects who slept for equal to or more than 9 hours increased.

For peer review only - http://bmjopen.bmj.com/site/about/guidelines.xhtml

Table 1 Distributions of sleep duration by categorical basic characteristics and descriptive statistics of continuous covariates in the sleep duration groups

Variables	Sleep duration(hours) <6	6	7	8	≥9	Overall	χ ²/F	P value
Number of subjects, n(%)	9576(17.2)	8988(16.14)	11159(20.04)	17790(31.95)	8174(14.68)	55687(100)		
Gender, n(%)								
Female	6271(18.24)	5273(15.34)	6594(19.18)	10898(31.71)	5336(15.52)	34372(100)	171.051	<.0001
Male	3305(15.51)	3715(17.43)	4565(21.42)	6892(32.33)	2838(13.31)	21315(100)		
Age(years), n(%)								
30~39	604(6.21)	998(10.27)	1802(18.54)	4142(42.60)	2176(22.38)	9722(100)	4406.276	<.0001
40~49	1651(11.01)	2087(13.91)	3170(21.13)	5523(36.82)	2571(17.14)	15002(100)		
50~59	3560(19.69)	3278(18.13)	3815(21.11)	5262(29.11)	2161(11.96)	18076(100)		
60~69	2775(27.41)	2050(20.25)	1962(19.38)	2340(23.12)	996(9.84)	10123(100)		
70~79	986(35.67)	575(20.80)	410(14.83)	523(18.92)	270(9.77)	2764(100)		
Education level, n(%)								
No formal school	2610(30.38)	1555(18.10)	1452(16.90)	1942(22.60)	1033(12.02)	8592(100)	2440.546	<.0001
Primary school	5146(18.53)	4759(17.14)	5539(19.95)	8401(30.26)	3920(14.12)	27765(100)		
Middle school	1444(9.70)	2040(13.70)	3059(20.55)	5722(38.44)	2621(17.61)	14886(100)		
High school and above	376(8.46)	634(14.27)	1109(24.95)	1725(38.82)	600(13.50)	4444(100)		
Occupation, n(%)								
Famer	8674(17.73)	7862(16.07)	9629(19.68)	15531(31.74)	7236(14.79)	48932(100)	102.183	<.0001
Others	902(13.35)	1126(16.67)	1530(22.65)	2259(33.44)	938(13.89)	6755(100)		
Marital status, n(%)								
Married	8040(15.91)	7990(15.81)	10236(20.26)	16610(32.87)	7650(15.14)	50526(100)	797.729	<.0001
Never Married/Widowed /Separated/Divorced	1536(29.76)	998(19.34)	923(17.88)	1180(22.86)	524(10.15)	5161(100)		
Annual household income (RMB, yuan) , n(%)								
<5,000	3730(24.61)	2454(16.19)	2761(18.22)	3996(26.37)	2215(14.61)	15156(100)	991.831	<.0001
5000~9,999	3145(15.98)	3168(16.09)	3867(19.65)	6532(33.18)	2972(15.10)	19684(100)		
≥10,000	2701(12.96)	3366(16.15)	4531(21.73)	7262(34.83)	2987(14.33)	20847(100)		
Tea consumption, n(%)								
Never or almost never	2311(23.51)	1534(15.61)	1768(17.99)	2692(27.39)	1525(15.51)	9830(100)	581.800	<.0001
Only occasionally	4822(17.76)	4488(16.53)	5385(19.83)	8695(32.02)	3764(13.86)	27154(100)		
Usually at least once a week	2443(13.06)	2966(15.86)	4006(21.42)	6403(34.24)	2885(15.43)	18703(100)		
Smoking status, n(%)								
Never	4896(16.38)	4418(14.78)	5771(19.30)	9998(33.44)	4814(16.10)	29897(100)	350.690	<.0001
Ex-regular smoker	835(22.14)	675(17.90)	786(20.84)	996(26.41)	479(12.70)	3771(100)		
Occasional smoker	892(20.14)	827(18.67)	892(20.14)	1272(28.71)	547(12.35)	4430(100)		
Daily or almost every day	2953(16.79)	3068(17.44)	3710(21.09)	5524(31.41)	2334(13.27)	17589(100)		
Alcohol intake, n(%)								
Never regular drinker	3591(18.80)	2926(15.32)	3575(18.72)	6031(31.57)	2979(15.60)	19102(100)	204.099	<.0001

For peer review only - http://bmjopen.bmj.com/site/about/guidelines.xhtml

Ex-regular drinker	463(22.43)	371(17.97)	439(21.27)	512(24.81)	279(13.52)	2064(100)		
Occasional or seasonal drinker	3548(16.40)	3456(15.97)	4439(20.51)	7118(32.89)	3078(14.22)	21639(100)		
Usually at least once a week	1974(15.32)	2235(17.35)	2706(21.01)	4129(32.05)	1838(14.27)	12882(100)		
Fresh fruit consumption, n(%)								
Daily	736(11.53)	863(13.52)	1376(21.56)	2218(34.76)	1188(18.62)	6381(100)	1185.806	<.0001
4-6 days per week	716(12.75)	814(14.50)	1134(20.20)	1931(34.39)	1020(18.17)	5615(100)		
1-3 days per week	3688(15.35)	3720(15.49)	4654(19.38)	8347(34.75)	3611(15.03)	24020(100)		
Monthly	3401(21.93)	2890(18.63)	3210(20.69)	4262(27.48)	1748(11.27)	15511(100)		
Never/rarely	1035(24.88)	701(16.85)	785(18.87)	1032(24.81)	607(14.59)	4160(100)		
BMI(Kg/m2), n(%)								
<18.5	625(25.31)	436(17.66)	462(18.71)	642(26.00)	304(12.31)	2469(100)	161.222	<.0001
18.5~23.9	5523(17.33)	5094(15.99)	6399(20.08)	10209(32.04)	4642(14.57)	31867(100)		
24~27.9	2700(16.12)	2718(16.23)	3393(20.26)	5416(32.34)	2519(15.04)	16746(100)		
≥28	728(15.81)	740(16.07)	905(19.65)	1523(33.07)	709(15.40)	4605(100)		
DM, n(%)								
No	9050(16.89)	8606(16.06)	10751(20.07)	17257(32.21)	7908(14.76)	53572(100)	118.858	<.0001
Yes	526(24.87)	382(18.06)	408(19.29)	533(25.20)	266(12.58)	2115(100)		
CHD, n(%)								
No	9501(17.15)	8929(16.11)	11101(20.03)	17734(32.00)	8147(14.70)	55412(100)	37.361	<.0001
Yes	75(27.27)	59(21.45)	58(21.09)	56(20.36)	27(9.82)	275(100)		
Stroke, n(%)								
No	9509(17.17)	8941(16.14)	11115(20.06)	17719(31.99)	8112(14.64)	55396(100)	23.279	0.0001
Yes	67(23.02)	47(16.15)	44(15.12)	71(24.40)	62(21.31)	291(100)		
Hypertension, n(%)								
No	6530(15.67)	6527(15.66)	8473(20.33)	13865(33.27)	6278(15.06)	41673(100)	362.709	<.0001
Yes	3046(21.74)	2461(17.56)	2686(19.17)	3925(28.01)	1896(13.53)	14014(100)		
Age(years), Mean (SD)	56.30±9.94	53.37±10.19	50.8±10.07	48.60±10.09	47.9±10.31	51.03±10.54	1224.030	<.0001
MET(hours/day), Mean (SD)	21.08±11.47	21.98±11.89	22.67±12.03	22.72±11.65	21.12±11.70	22.07±11.76	51.170	<.0001
WC(cm), Mean (SD)	77.45±9.27	78.27±9.11	78.16±8.98	78.07±8.96	77.99±9.05	78.00±9.06	11.800	<.0001
BMI(Kg/m2), Mean (SD)	23.00±3.29	23.29±3.21	23.32±3.18	23.38±3.15	23.39±3.19	23.29±3.20	25.120	<.0001
Sedentary leisure time(hours/week), Mean (SD)	25.15±10.22	25.83±10.45	25.98±10.50	26.85±10.53	27.28±11.39	26.28±10.61	64.490	<.0001
SBP(mmHg), Mean (SD)	132.28±20.24	130.23±19.23	128.61±18.73	127.81±18.18	128.52±18.96	129.24±19.01	99.140	<.0001
DBP(mmHg), Mean (SD)	77.65±10.51	77.34±10.34	76.96±10.24	77.02±9.97	77.45±10.28	77.23±10.23	8.940	<.0001

For peer review only

For peer review only - http://bmjopen.bmj.com/site/about/guidelines.xhtml

The percentages in table are row percent. χ^2 tests were conducted to test for differences in percentage for categorical variables.One-way anova tests were conducted to test for differences in means for categorical variables. Abbreviations: BMI, body mass index; DM, diabetes mellitus; CHD, Coronary Heart Disease; WC, waist circumference; MET, Metabolic Equivalent; SBP, Systolic Blood Pressure; DBP, diastolic blood pressure.

The prevalence of hypertension among adults in southwest China

Results of prevalence of hypertension in different gender groups among 55687 adults were presented in **Table 2**. 25.17% of 55687 adults had hypertension. A quarter of participants were considered hypertensive, slightly more in men (28.67%) than in women (22.99%). The prevalence of hypertension in both men and women increased with age. The prevalence of hypertension decreased with increased education level. The prevalence of hypertension in both males and females decreased with increased annual household income and tea consumption. Among different alcohol intake and smoking status groups, the highest prevalences of hypertension were observed in ex-regular drinker and ex-regular smoker in males and females.

The prevalence of hypertension increased with decreased fresh fruit consumption in males and females. The prevalence of hypertension in both males and females increased with BMI and sleep snoring frequency. Higher prevalence of hypertension was also associated with insomnia, DM, stroke, CHD in both males and females. The prevalence of hypertension in DM patients (48.98%) was twice as high as that in non-DM subjects (24.23%). The prevalence of hypertension in stroke patients (73.54%) was nearly triple that in non-stroke subjects (24.91%). The prevalence of hypertension in both males and females decreased with sleep duration.

For peer review only

For peer review only - http://bmjopen.bmj.com/site/about/guidelines.xhtml

Table 2 Prevalence of hypertension in different gender groups among 55687 adults in southwest China

Variables	Male n(Prevalence %)	Male P value	Female n(Prevalence %)	Female P value	Overall n(Prevalence %)	Overall P value
Total	6111(28.67)	-	7903(22.99)	-	14014(25.17)	<.0001
Age(years)						
30~39	417(12.76)	<.0001	331(5.13)	<.0001	748(7.69)	<.0001
40~49	905(17.47)		1259(12.82)		2164(14.42)	
50~59	1965(28.45)		3243(29.04)		5208(28.81)	
60~69	2115(45.98)		2361(42.75)		4476(44.22)	
70~79	709(52.21)		709(50.43)		1418(51.30)	
Education level						
No formal school	847(41.52)	<.0001	2410(36.78)	<.0001	3257(37.91)	<.0001
Primary school	3481(31.07)		3994(24.12)		7475(26.92)	
Middle school	1235(21.21)		1190(13.13)		2425(16.29)	
High school and above	548(24.37)		309(14.08)		857(19.28)	
Annual household income(RMB, yuan)						
<5,000	2129(37.15)	<.0001	3000(31.83)	<.0001	5129(33.84)	<.0001
5,000~9,999	1852(26.25)		2563(20.29)		4415(22.43)	
≥10,000	2130(24.97)		2340(19.00)		4470(21.44)	
Tea consumption						
Never or almost never	482(33.04)	0.0207	2335(27.89)	<.0001	2817(28.66)	0.0281
Only occasionally	1908(28.19)		4396(21.56)		6304(23.22)	
Usually at least once a week	3721(28.43)		1172(20.87)		4893(26.16)	
Smoking status						
Never	726(28.96)	<.0001	5737(20.95)	<.0001	6463(21.62)	<.0001
Ex-regular smoker	857(35.34)		520(38.63)		1377(36.52)	
Occasional smoker	527(25.41)		710(30.14)		1237(27.92)	
Smoked daily or almost every day	4001(27.96)		936(28.54)		4937(28.07)	
Alcohol intake						
Never regular drinker	663(28.54)	<.0001	4175(24.88)	<.0001	4838(25.33)	<.0001
Ex-regular drinker	572(40.97)		257(38.47)		829(40.16)	
Occasional or seasonal drinker	1646(24.00)		2830(19.14)		4476(20.68)	
Usually at least once a week	3230(30.08)		641(29.91)		3871(30.05)	

For peer review only
For peer review only - http://bmjopen.bmj.com/site/about/guidelines.xhtml

Fresh fruit consumption						
Daily	549(26.99)	<.0001	819(18.84)	<.0001	1368(21.44)	<.0001
4-6 days per week	482(26.41)		755(19.92)		1237(22.03)	
1-3 days per week	2387(26.61)		3249(21.59)		5636(23.46)	
Monthly	1957(30.08)		2364(26.25)		4321(27.86)	
Never/rarely	736(37.13)		716(32.87)		1452(34.90)	
Insomnia						
No	4650(27.66)	<.0001	5244(21.01)	<.0001	9894(23.69)	<.0001
Yes	1461(32.42)		2659(28.24)		4120(29.59)	
Sleep snoring						
Never	2007(25.39)	<.0001	3391(18.08)	<.0001	5398(20.25)	<.0001
Occasionally	1481(27.88)		1870(24.94)		3351(26.16)	
Usually	2623(32.39)		2642(32.54)		5265(32.47)	
BMI (Kg/m2)						
<18.5	177(19.80)	<.0001	254(16.13)	<.0001	431(17.46)	<.0001
18.5~23.9	3331(24.51)		3170(17.34)		6501(20.40)	
24~27.9	1999(35.59)		3080(27.68)		5079(30.33)	
≥28	604(49.67)		1399(41.28)		2003(43.50)	
DM						
No	5726(27.90)	<.0001	7252(21.94)	<.0001	12978(24.23)	<.0001
Yes	385(48.67)		651(49.17)		1036(48.98)	
CHD						
No	6049(28.54)	<.0001	7839(22.91)	<.0001	13888(25.06)	<.0001
Yes	62(52.99)		64(40.51)		126(45.82)	
Stroke						
No	5999(28.35)	<.0001	7801(22.79)	<.0001	13800(24.91)	<.0001
Yes	112(73.20)		102(73.91)		214(73.54)	
Sleep duration(hours)						
<6	1113(33.68)	<.0001	1933(30.82)	<.0001	3046(31.81)	<.0001
6	1108(29.83)		1353(25.66)		2461(27.38)	
7	1263(27.67)		1423(21.58)		2686(24.07)	
8	1803(26.16)		2122(19.47)		3925(22.06)	
≥9	824(29.03)		1072(20.09)		1896(23.20)	

"n(Prevalence,%)" represents the number of hypertension and the prevalence. χ^2 tests were conducted to test for differences in prevalence for unordered categorical variables and Chi-square tests for trend were used to examine the trends for ordered categorical variables.

For peer review only - http://bmjopen.bmj.com/site/about/guidelines.xhtml

Results of prevalence of hypertension and distribution of hypertension patients in different sleep duration groups were presented in **Table 3**. In different sleep groups, the prevalences of hypertension were more in men than in women.The prevalence of hypertension increased with age. The prevalence of hypertension decreased with increased fresh fruit consumption. Among different alcohol intake and smoking status groups, the highest prevalences of hypertension were observed in ex-regular drinker and ex-regular smoker in different sleep duration groups. Among 7 and 8 hours sleepers, the prevalence of hypertension was higher among insomnia subjects than that among non-insomnia groups.

The percentages of the hypertension patients with sleep durations of <6, 6, 7, 8 and ≥ 9 hours were 21.74%, 17.56%, 19.17%, 28.00% and 13.53%, respectively. Patients with less hours of sleep were more likely to be female, eldly, never or almost never tea consumers, low frequency of fresh fruit consumption. With the increase of age, the proportion of hypertension patients who slept for equal to or more than 8 hours decreased.

For peer review only - http://bmjopen.bmj.com/site/about/guidelines.xhtml

Table 3 Prevalence of hypertension and distribution of hypertension patients in different sleep duration groups among 55687 adults in southwest China

	Prevalence of hypertension(%)					n (%)						
**Variables**	**<6**	**6**	**7**	**8**	**≥9**	**<6**	**6**	**7**	**8**	**≥9**	**overall**	**P value**
Total	31.81	27.38	24.07	22.06	23.20	3046(21.74)	2461(17.56)	2686(19.17)	3925(28.00)	1896(13.53)	14014(100)	<.0001
Gender												
Female	30.82	25.66	21.58	19.47	20.09	1933(24.46)	1353(17.12)	1423(18.01)	2122(26.85)	1072(13.56)	7903(100)	<.0001
Male	33.68	29.83	27.67	26.16	29.03	1113(18.21)	1108(18.13)	1263(20.67)	1803(29.51)	824(13.48)	6111(100)	
P value	0.0044	<.0001	<.0001	<.0001	<.0001							
Age(years)												
30~39	7.45	7.52	8.49	7.48	7.58	45(6.02)	75(10.03)	153(20.45)	310(41.44)	165(22.06)	748(100)	<.0001
40~49	15.38	13.18	13.85	14.39	15.60	254(11.74)	275(12.71)	439(20.29)	795(36.73)	401(18.53)	2164(100)	
50~59	30.17	27.79	27.10	28.18	32.67	1074(20.62)	911(17.49)	1034(19.85)	1483(28.48)	706(13.56)	5208(100)	
60~69	42.56	44.00	43.48	45.47	47.79	1181(26.39)	902(20.15)	853(19.06)	1064(23.77)	476(10.63)	4476(100)	
70~79	49.9	51.83	50.49	52.20	54.81	492(34.70)	298(21.01)	207(14.60)	273(19.25)	148(10.44)	1418(100)	
P value	<.0001	<.0001	<.0001	<.0001	<.0001							
Tea consumption												
Never or almost never	35.05	29.14	27.32	24.63	27.15	810(28.75)	447(15.87)	483(17.15)	663(23.53)	414(14.70)	2817(100)	<.0001
Only occasionally	30.38	26.23	22.10	19.79	19.95	1465(23.24)	1177(18.67)	1190(18.88)	1721(27.30)	751(11.91)	6304(100)	
Usually at least once a week	31.56	28.22	25.29	24.07	25.34	771(15.76)	837(17.11)	1013(20.70)	1541(31.49)	731(14.94)	4893(100)	
P value	0.0117	0.9427	0.7805	0.1173	0.955							
Smoking status												
Never	29.31	23.74	20.90	18.49	19.19	1435(22.20)	1049(16.23)	1206(18.66)	1849(28.61)	924(14.30)	6463(100)	<.0001
Ex-regular smoker	39.16	38.07	34.10	34.74	37.37	327(23.75)	257(18.66)	268(19.46)	346(25.13)	179(13.00)	1377(100)	
Occasional smoker	32.96	29.87	25.45	25.79	25.78	294(23.77)	247(19.97)	227(18.35)	328(26.51)	141(11.40)	1237(100)	

For peer review only

Smoked daily or almost every day	33.53	29.60	26.55	25.38	27.93	990(20.05)	908(18.39)	985(19.95)	1402(28.40)	652(13.21)	4937(100)	
P value	0.0001	<.0001	<.0001	<.0001	<.0001							
Alcohol consumption												
Never regular drinker	33.14	26.73	24.31	22.22	22.05	1190(24.60)	782(16.16)	869(17.96)	1340(27.70)	657(13.58)	4838(100)	<.0001
Ex-regular drinker	40.60	41.24	36.90	37.70	47.67	188(22.68)	153(18.46)	162(19.54)	193(23.28)	133(16.04)	829(100)	
Occasional or seasonal drinker	27.73	23.87	19.71	17.43	17.90	984(21.98)	825(18.43)	875(19.55)	1241(27.73)	551(12.31)	4476(100)	
Usually at least once a week	34.65	31.36	28.82	27.88	30.20	684(17.67)	701(18.11)	780(20.15)	1151(29.73)	555(14.34)	3871(100)	
P value	<.0001	<.0001	<.0001	<.0001	<.0001							
Fresh fruit consumption												
Daily	28.13	23.41	22.97	18.49	19.61	207(15.13)	202(14.77)	316(23.10)	410(29.97)	233(17.03)	1368(100)	<.0001
4-6 days per week	26.26	26.41	21.34	19.63	20.88	188(15.20)	215(17.38)	242(19.56)	379(30.64)	213(17.22)	1237(100)	
1-3 days per week	29.34	25.56	23.18	20.97	21.43	1082(19.20)	951(16.87)	1079(19.14)	1750(31.06)	774(13.73)	5636(100)	
Monthly	34.49	29.55	24.64	24.61	25.97	1173(27.15)	854(19.76)	791(18.31)	1049(24.27)	454(10.51)	4321(100)	
Never/rarely	38.26	34.09	32.87	32.66	36.57	396(27.27)	239(16.46)	258(17.77)	337(23.21)	222(15.29)	1452(100)	
P value	<.0001	<.0001	<.0001	<.0001	<.0001							
Insomnia												
No	30.70	27.66	23.59	21.77	23.06	634(6.41)	1621(16.38)	2248(22.72)	3617(36.56)	1774(17.93)	9894(100)	<.0001
Yes	32.11	26.85	26.89	26.24	25.42	2412(58.54)	840(20.39)	438(10.63)	308(7.48)	122(2.96)	4120(100)	
P value	0.2228	0.4132	0.004	0.0004	0.2347							

"n(%)" represents the number of hypertension and the percentages. The percentages in table are row percent. χ^2 tests were conducted to test for differences in percentage for categorical variables.

χ^2 tests were conducted to test for differences in prevalence for unordered categorical variables and Chi-square tests for trend were used to examine the trends for ordered categorical variables.

For peer review only

For peer review only - http://bmjopen.bmj.com/site/about/guidelines.xhtml

The relationship between sleep duration and hypertension

Results of the multivariable logistic regression analyses examined relationship between sleep duration and hypertension were presented in **Table 4**. Compared to 7-hours sleepers (reference group), the unadjusted analyses showed that short sleepers (< 6 hours and 6 hours) having higher odds of hypertension (OR = 1.47 and 1.19, respectively; $P < 0.05$). After adjustment for age, sex, education level, occupation, marital status, annual household income, tea consumption, smoking status, alcohol intake, MET, sedentary leisure time, fresh fruit consumption, insomnia, sleep snoring, BMI, WC, DM, stroke and CHD, the increased odds of having hypertension was across those who reported ≥ 9 hours of sleep (OR = 1.17, 95% CI: 1.08-1.26; $P < 0.05$). However, short sleep duration was no longer associated with hypertension. There were similar results in men and women after adjustment for potential confounding factors. Long sleep duration was associated with elevated hypertension prevalence, with long sleepers (≥ 9 hours) having higher odds of hypertension in men (OR = 1.16, 95% CI : 1.04-1.30; $P < 0.05$) and women (OR = 1.19, 95% CI : 1.08-1.32; $P < 0.05$). The observed association between sleep duration and hypertension was slightly attenuated among men compared with women.

For peer review only - http://bmjopen.bmj.com/site/about/guidelines.xhtml

Table 4 Multivariable adjusted odds ratio by logistic regression analyses of sleep duration associated with hypertension

**Population**	**Sleep duration(hours)**				
	**<6**	**6**	**7**	**8**	**≥9**
Total[a], n/N	3046/9576	2461/8988	2686/11159	3925/17790	1896/8174
Model1	**1.47(1.38-1.56)**	**1.19(1.12-1.27)**	1	0.89(0.84-0.94)	0.95(0.89-1.02)
Model2	1.02(0.96-1.09)	0.99(0.93-1.06)	1	1.05(0.99-1.11)	**1.19(1.10-1.27)**
Model3	1.02(0.95-1.09)	0.99(0.93-1.06)	1	1.05(0.99-1.11)	**1.18(1.10-1.27)**
Model4	0.98(0.91-1.05)	0.98(0.92-1.05)	1	1.05(0.99-1.12)	**1.16(1.08-1.24)**
Model5	0.96(0.89-1.04)	0.96(0.90-1.03)	1	1.06(0.99-1.12)	**1.17(1.08-1.26)**
Male[b], n/N	1113/3305	1108/3715	1263/4565	1803/6892	824/2838
Model1	**1.33(1.21-1.46)**	**1.11(1.01-1.22)**	1	0.93(0.85-1.01)	1.07(0.96-1.19)
Model2	0.97(0.87-1.07)	0.97(0.88-1.07)	1	1.03(0.94-1.13)	**1.19(1.07-1.33)**
Model3	0.96(0.86-1.06)	0.97(0.87-1.07)	1	1.03(0.95-1.13)	**1.19(1.07-1.33)**
Model4	0.92(0.82-1.03)	0.95(0.86-1.05)	1	1.04(0.95-1.14)	**1.17(1.05-1.31)**
Model5	0.89(0.79-1.00)	0.93(0.84-1.03)	1	1.04(0.95-1.14)	**1.16(1.04-1.30)**
Female[b], n/N	1933/6271	1353/5273	1423/6594	2122/10898	1072/5336
Model1	**1.62(1.50-1.75)**	**1.25(1.15-1.37)**	1	0.88(0.82-0.95)	0.91(0.84-1.00)
Model2	1.05(0.97-1.15)	1.01(0.92-1.11)	1	1.07(0.98-1.15)	**1.20(1.09-1.32)**
Model3	1.05(0.96-1.14)	1.01(0.92-1.11)	1	1.07(0.99-1.16)	**1.20(1.09-1.32)**
Model4	1.01(0.91-1.11)	1.00(0.91-1.09)	1	1.07(0.98-1.16)	**1.17(1.06-1.29)**
Model5	1.00(0.90-1.11)	0.98(0.89-1.07)	1	1.07(0.99-1.17)	**1.19(1.08-1.32)**

Data were analyzed as odds ratios (95% CIs) for the "presence of hypertension" relative to "without hypertension" by logistic regression models using the 7 hours sleep duration as the reference group.

a. Model 1 was unadjusted; Model 2 was adjusted for age and sex; Model 3 was adjusted for model 2 + education level, occupation, marital status, annual household income; Model 4 was adjusted for model 3 + tea consumption, smoking status, alcohol intake, MET, sedentary leisure time, fresh fruit consumption, insomnia, sleep snoring; Model 5 was adjusted for model 4 + BMI, WC, DM, stroke and CHD.

b. Model 1 was unadjusted; Model 2 was adjusted for age; Model 3 was adjusted for model 2 + education level, occupation, marital status, annual household income; Model 4 was adjusted for model 3 + tea consumption, smoking status, alcohol intake, MET, sedentary leisure time, fresh fruit consumption, insomnia, sleep snoring; Model 5 was adjusted for model 4 + BMI, WC, DM, stroke and CHD.

"n/N" represents the number of hypertension and the number of participants in each subgroup.

For peer review only

The relationship between sleep duration and hypertension stratification by health-related factors

Results of covariate-adjusted analyses stratified by health-related factors examining relationship between sleep duration and hypertension were displayed in **Figure 1**. Long sleep was associated with elevated hypertension prevalence for 45-59 and 60-79 years old subgroups,

though the relationship was nominally stronger among 45-59 individuals. However, this association was not found among 30-44 years old subgroup. Among those usually at least once a week tea consumers, long sleep was associated with hypertension, and the relationship was not present across never/almost never or only occasionally tea consumers. Among those who were never regular alcohol drinker, ex-regular or usually at least once a week alcohol drinker, long sleep duration was associated with elevated hypertension prevalence. However, among occasional or seasonal alcohol drinker, the relationship with long sleep was not present. Among different smoking status subgroups, long sleep was associated with hypertension among never smoker and daily or almost every day smoker, and this association was not observed among ex-regular and occasional smoker. Long sleep was associated with elevated hypertension prevalence for non-regular fresh fruit consumers, while not among regular fresh fruit consumers. Long sleep was associated with elevated hypertension prevalence for insomnia subgroup, while not among non-insomnia subgroup.

The dose-response relationship between sleep duration and hypertension by restricted cubic splines regression

Results of restricted cubic splines regression to calculate and visualize the relationship of sleep duration with hypertension were displayed in **Figure 2**. The odds of hypertension was relatively flat until around 6.81 hours of sleep duration and then started to increase rapidly afterwards in subjects and a J-shaped pattern was observed (P for nonlinearity = 0.0023). Similar patterns were also observed in male subjects and more than 45 years old subgroups. However, the non-linear tests were not significant for males and subjects more than 60 years old (P for nonlinear > 0.05).There was a U-shaped relationship between sleep duration and hypertension in females (P for nonlinearity = 0.0081). Similar pattern was also observed in 30~44 years old subjects, but the non-linear test was not significant (P for nonlinearity = 0.2481).

For peer review only - http://bmjopen.bmj.com/site/about/guidelines.xhtml

The relationship between sleep duration and blood pressure

Age and gender-specific linear regression coefficients of sleep duration for SBP and DBP were presented in **Table 5**. We estimated regression models separately for different age groups in two genders and then in the general population. After adjusting for covariates as mentioned in Table 4, sleep duration showed significant and positive associations with SBP and DBP both in males and females. Regression coefficients (SEs) of sleep duration and SBP were 0.474 (0.088), 0.417 (0.070) and 0.408(0.055) in males, females and general population, respectively. Regression coefficients (SEs) of sleep duration and DBP were 0.203 (0.051),0.102 (0.039) and 0.131(0.031) in males, females, and general population, respectively. There was a positive relationship of sleep duration with blood pressure, with 1 hour higher sleep duration associated with 0.474/0.203, 0.417/0.102 and 0.408/0.131 mm Hg higher SBP/DBP in males, females, and general population, respectively. Age-specific linear regression coefficients of sleep duration for SBP and DBP were elevated with age. The association patterns were substantially similar for SBP in two genders. However, no significant associations were found between sleep duration and SBP in 30~44 years group among males. Only the association parameters for DBP were significant in 45~59 years old males and 30~44 years old females.

Table 5 Regression coefficients (SEs) of sleep duration associated with SBP and DBP by gender and age groups

Variables	SBP				DBP			
	30~44 years	45~59 years	≥60 years	Total	30~44 years	45~59 years	≥60 years	Total
Overall [b]								
Number of participants	18787	24013	12887	55687	18787	24013	12887	55687
Regression coefficients (SEs)	0.219(0.073)	0.424(0.087)	0.646(0.131)	0.408(0.055)	0.116(0.050)	0.153(0.048)	0.166(0.069)	0.131(0.031)
P value	< 0.001	< 0.001	< 0.001	< 0.001	< 0.05	< 0.01	< 0.05	< 0.001
Male [a]								
Number of participants	6364	8993	5958	21315	6364	8993	5958	21315
Regression coefficients (SEs)	0.213(0.123)	0.566(0.134)	0.554(0.195)	0.474(0.088)	0.072(0.09)	0.320(0.079)	0.194(0.103)	0.203(0.051)

For peer review only - http://bmjopen.bmj.com/site/about/guidelines.xhtml

P value	> 0.05	< 0.001	< 0.001	< 0.001	> 0.05	< 0.001	> 0.05	< 0.001
Female [a]								
Number of participants	12423	15020	6929	34372	12423	15020	6929	34372
Regression coefficients (SEs)	0.227(0.091)	0.378(0.113)	0.716(0.178)	0.417(0.070)	0.139(0.061)	0.070(0.061)	0.150(0.092)	0.102(0.039)
P value	< 0.05	< 0.001	< 0.001	< 0.001	< 0.05	> 0.05	> 0.05	< 0.01

a. Age, education level, occupation, marital status, income, tea consumption, smoking status, alcohol intake, MET, sedentary leisure time, fresh fruit consumption, insomnia, sleep snoring, BMI, WC, DM, stroke and CHD were included in models for adjustment.

b. Age, sex, education level, occupation, marital status, income, tea consumption, smoking status, alcohol intake, MET, sedentary leisure time, fresh fruit consumption, insomnia, sleep snoring, BMI, WC, DM, stroke and CHD were included in models for adjustment.

Abbreviations: SBP, systolic blood pressure; DBP, diastolic blood pressure.

For peer review only

DISCUSSION

This study examined the relationship between sleep duration and hypertension among more than 50 000 rural adults in southwest China. Long sleep duration was associated with hypertension among men, women and the general population, which was consistent with several previous studies in different countries.[16-18 31] For example, the first study assessed the association between sleep duration and hypertension conducted by Gottlieb et. al[17] reported that compared to subjects sleeping 7 to less than 8 hours per night, the adjusted ORs (95% CIs) for hypertension of those sleeping 8 hours and more than 9 hours were 1.19 (1.04-1.37) and 1.30 (1.04-1.62), respectively. Long sleep duration of equal to or more than 8 hours per night was associated with hypertension in participants aged 40-100 years. The Nurse' health study[31] found long sleep duration ($\geq$ 9 hours) was associated with elevated hypertension prevalence in cross-sectional study. A systematic review and meta-analysis conducted by Guo et. al[32] also found that long sleep duration was associated with hypertension (OR = 1.11, 95% CI: 1.04-1.18). Another meta-analysis conducted by Wang et. al[33] indicated that the OR (95% CI) for long sleep duration and hypertension was 1.11 (1.05-1.17).

However, numerous studies showed that compared with normal sleep duration, short sleep duration was more likely to be associated with hypertension than long sleep duration.[9-12 34 35] A cross-sectional study conducted among rural population in China showed that short sleep duration was associated with hypertension in women.[35] Another cross-sectional study in China including 43 655 participants found that short sleep duration of less than 6 hours was related to hypertension in both genders.[10] Results from the Kaiyuan study showed that short sleep duration ($\leq$ 5 hours) was associated with an increased incidence of hypertension during the 3.98 years follow-up period among women and those aged less than 60 years old.[9] A prospective study conducted by Gangwisch et. al reported that short sleep duration ($\leq$ 5 hours) could significantly elavate the risk of hypertension (HR = 2.10, 95% CI: 1.58-2.79) in subjects between 32 to 59 years old.[11]

In this study, restricted cubic splines regression was used to visualize the relationship of sleep duration and hypertension. The odds of hypertension was relatively flat until around 6.81

For peer review only - http://bmjopen.bmj.com/site/about/guidelines.xhtml

hours of sleep duration and then started to increase rapidly afterwards and a J-shaped pattern was observed among adults. There was a U-shaped association between sleep duration and hypertension in females. However, inconsistent with many previous studies,[15-17 36] the association between short sleep duration and hypertension was not obvious in this study. There were plausible explanations for the different results of these studies. First, different definition of sleep duration groups and adjustment of different possible influencing factors would lead to differences in statistical analysis. Second, the discrepancy may be due to the difference of study population. Different demographic factors and behavioral habits also would lead to the differences of the relationship between sleep duration and hypertension. Previous studies indicated that both insomnia[37] and snoring[38] could elevated risk of hypertension, and our study adjusted these factors for multivariate analysis. In addition, relationship of sleep duration and blood pressure was also explored in this study. After adjusting for potential confounders, sleep duration showed positive associations with blood pressure both in females and males. Regression coefficients of sleep duration and SBP/DBP were 0.474/0.203 in males and 0.417/0.102 in females. These results revealed that sleep duration was more closely associated with SBP than DBP.

The association between sleep duration and hypertension could reflect a variety of mechanisms. Several studies indicated that potential pathophysiological mechanisms supporting the association between short sleep duration and hypertension in humans may be sleep deprivation. Sleep deprivation has been reported as a cause of over-activity of the sympathetic nervous system, which may elevate blood pressure.[11 39] Sleep deprivation could disrupt circadian rhythmicity, contribute to over-activity of the renin-angiotensin aldosterone system, pro-inflammatory responses, endothelial dysfunction, and renal impairment. Furthermore, sleep deprivation could increase appetite and suppress renal salt fluid excretion, leading to elevated caloric consumption, excessive salt intake and weight gain, which were proved to be risk factors of hypertension.[11 40] However, the mechanisms of long sleep duration with hypertension were even not known. Possible explanations may be as follows. First, some other risk factors might impact this association. Long sleepers may have less physical

For peer review only - http://bmjopen.bmj.com/site/about/guidelines.xhtml

activities,[41] and be related to sleep disorders (sleep apnea, etc.) or poor sleep quality, which could lead to adverse healthy outcomes.[16] In previous studies, long sleep was associated with increased all-cause mortality, high risk of diabetes and other unhealthy outcomes.[41-43] Second, sleep duration may reflect sleep need, which is different in individuals due to genetic, behavioral, and environmental factors.[17] Individuals with increased sleep needs may have a reduced physiologic reserve, reducing their ability to survive serious illness.[41] Third, sleep habits may be a marker of health status rather than a cause for disease, so it is likely that long sleep duration is an epiphenomenon instead of an etiological factor for hypertension.[18 44]

The relationship between sleep duration and hypertension may varied by health-related factors. This study made stratified analyses of sleep duration and hypertension stratified by age, tea consumption, alcohol intake, smoking status, fresh fruit consumption and insomnia. Long sleep were associated with elevated hypertension prevalence among participants aged 45 to 79 years old. However, this association was not found among those aged 30-44 years old. By restricted cubic splines regression, a J-shaped pattern was observed in 45-59 years old subgroups. These findings were different from several previous studies. For example, a Korean study found that short sleep duration was associated with hypertension prevalence only in those aged less than 60 years.[45] A Spanish study demonstrated that sleep duration was not associated with hypertension in older adults.[46] A cross sectional study conducted in northeast China observed an association between short sleep duration and hypertension in young adults aged 18 to 44 years, and the relationship was not found for participants aged 45 to 79 years subgroup.[19] Changes in sleep quality and quantity in different stage of life may be related to this age-dependent association.[47] In addition, healthy sleep was influenced by a lot of factors. Besides sleep duration, sleep disorders such as insomnia,[48 49] obstructive sleep apnea[50] and other sleep quality problems[51-53] have also been shown to be risk factors for hypertension. Moreover, the number of younger subjects was larger than that of older subjects, so the lack of an association between long sleep duration and hypertension in the younger subjects could be attribute to a lack of statistical power.

In this study, long sleep was associated with elevated hypertension prevalence for non-

For peer review only - http://bmjopen.bmj.com/site/about/guidelines.xhtml

regular fresh fruit consumers, while not among subjects who ate fresh fruit regularly. Fresh fruit intake could reduce the risk and mortality of cardiovascular disease. Results from previous prospective analyses of CKB showed that compared with participants who never or rarely consumed fresh fruit, those who ate fresh fruit daily had lower systolic blood pressure (by 4 mm Hg, P<0.001). The adjusted hazard ratios (95% CIs) for daily consumers were 0.60 (0.54 to 0.67) for cardiovascular death, and 0.66 (0.58 to 0.75), 0.75 (0.72 to 0.79), and 0.64 (0.56 to 0.74) for incident major coronary events, ischemic stroke, and hemorrhagic stroke, respectively.[54] Another prospective study of CKB found that fresh fruit intake reduced all-cause mortality, cardiovascular disease (CVD), cancer and chronic obstructive pulmonary disease mortality.[55] These results may explain the difference of relationship between long sleep duration and hypertension among non-regular and regular fresh fruit consumers. The association between long sleep duration and hypertension was more pronounced in individuals who had insomnia than non-insomnia subgroup. Two factors may explain the differential associations. First, insomnia has been proved to be a risk factor for hypertension.[37] Among insomnia participants, long sleep duration was more likely to be an unusual schedule, which could affect health outcomes in humans. Second, the sample size of non-insomnia subgroup was much higher than that of the insomnia group, which may lead to a statistical difference.

Our study had several strengths. This study was part of CKB, which was a large prospective cohort study in China. CKB had a series of standardized procedures and good quality control measures, so the data was relatively reliable. The sample size was large in this study, which could be a representative rural population in southwest China. Although this study revealed an association between long sleep duration and hypertension, several potential limitations of this study should be noted and need to be addressed in future work. First, our study was a cross-sectional study, so the directionality of the causation could not be established which did not allow for inferences regarding causation to be made. Further prospective cohort studies would be needed to confirm this association. Second, the data of sleep duration was self-reported without more detailed and objective measures of sleep, which might have information bias and was less accurate than actigraphy and polysomnography. Third, although

For peer review only - http://bmjopen.bmj.com/site/about/guidelines.xhtml

we have attempted to adjust for various potential confounders, our results may still be subject to residual confounding from unmeasured or unknown influencing factors.

CONCLUSIONS

In conclusion, this study identified long sleep duration was significantly associated with hypertension and a J-shaped pattern was observed amongadults in southwest China. However, this association was not found between short sleep duration and hypertension. It is important to sustain a proper amount of sleep duration for health. Further prospective studies are required to confirm these findings.

For peer review only

For peer review only - http://bmjopen.bmj.com/site/about/guidelines.xhtml

Acknowledgments We thank Chinese Center for Disease Control and Prevention, Chinese Ministry of Health, National Health and Family Planning Commission of China, and 10 provincial/regional Health Administrative Departments. The most important acknowledgement is to the participants in the study and the members of the survey teams in each of the 10 regional centers, as well as to the project development and management teams based at Beijing, Oxford and the 10 regional centers.

Contributors XW and GL conceived and designed the paper. LL and ZC designed and supervised the conduct of the CKB study, obtained funding and together with YG and PP acquired the data. XC, XC and JJ analysed the data. XC, XC, QS and NZ drafted the manuscript. XC, XC, JJ, GL, XC, QS, NZ and XW contributed to the interpretation of the results and critical revision of the manuscript. All authors contributed to and approved the final manuscript. XW is the study guarantor.

Funding This work was supported by grants ("Tianfu ten thousand talents plan" fund in 2018) from Sichuan Provincial Leading Group Office for Talent Work, grants (2016YFC0900500, 2016YFC0900501, 2016YFC0900504) from the National Key Research and Development Program of China, grants from the Kadoorie Charitable Foundation in Hong Kong (N/A), grants (212946/Z/18/Z, 202922/Z/16/Z, 104085/Z/14/Z, 088158/Z/09/Z) from Wellcome Trust in the UK. The funders had no role in the study design, data collection, data analysis and interpretation, writing of the report or the decision to submit the article for publication.

Competing interests None declared.

Patient consent for publication Not required.

Ethics approval The Ethical Review Committee of the Chinese Center for Disease Control and Prevention (Beijing, China) (Ethics ID number of approval: 005/2004) and the Oxford Tropical Research Ethics Committee, University of Oxford (UK) (Ethics ID number of approval: OXTREC 025-04).

Provenance and peer review Not commissioned; externally peer reviewed.

Data availability statement The dataset supporting the conclusions of this article is available from the study website (http://www.ckbiobank.org), along with the access policy and

For peer review only - http://bmjopen.bmj.com/site/about/guidelines.xhtml

procedures.

REFERENCES

1 Organization WH. A global brief on hypertension : silent killer, global public health crisis: World Health Day 2013. In. Geneva: World Health Organization 2013.

2 Wang Y, Peng X, Nie X*, et al*. Burden of hypertension in China over the past decades: Systematic analysis of prevalence, treatment and control of hypertension. *Eur J Prev Cardiol* 2016;23:792-800.

3 Wang Z, Chen Z, Zhang L*, et al*. Status of Hypertension in China: Results From the China Hypertension Survey, 2012-2015. *Circulation* 2018;137:2344-56.

4 Zhou M, Wang H, Zeng X*, et al*. Mortality, morbidity, and risk factors in China and its provinces, 1990-2017: a systematic analysis for the Global Burden of Disease Study 2017. *Lancet* 2019;394:1145-58.

5 Cao D, Zhou Z, Si Y*, et al*. Prevalence and income-related equity in hypertension in rural China from 1991 to 2011: differences between self-reported and tested measures. *BMC Health Serv Res* 2019;19:437.

6 Liu X, Zhang D, Liu Y*, et al*. Dose-Response Association Between Physical Activity and Incident Hypertension: A Systematic Review and Meta-Analysis of Cohort Studies. *Hypertension* 2017;69:813-20.

7 Forman JP, Stampfer MJ, Curhan GC. Diet and lifestyle risk factors associated with incident hypertension in women. *Jama* 2009;302:401-11.

8 Li H, Ren Y, Wu Y*, et al*. Correlation between sleep duration and hypertension: a dose-response meta-analysis. *J Hum Hypertens* 2019;33:218-28.

9 Song Q, Liu X, Wang X*, et al*. Age- and gender-specific associations between sleep duration and incident hypertension in a Chinese population: the Kailuan study. *J Hum Hypertens* 2016;30:503-7.

10 Guan Y, Zhang M, Zhang X*, et al*. Association between sleep duration and hypertension of migrant workers in China: a national cross-sectional surveillance study. *BMJ Open* 2019;9:e031126.

11 Gangwisch JE, Heymsfield SB, Boden-Albala B*, et al*. Short sleep duration as a risk factor for hypertension: analyses of the first National Health and Nutrition Examination Survey. *Hypertension* 2006;47:833-9.

12 Cappuccio FP, Stranges S, Kandala NB*, et al*. Gender-specific associations of short sleep duration with prevalent and incident hypertension: the Whitehall II Study. *Hypertension* 2007;50:693-700.

13 Kim J, Jo I. Age-dependent association between sleep duration and hypertension in the adult Korean population. *Am J Hypertens* 2010;23:1286-91.

14 Knutson KL, Van Cauter E, Rathouz PJ*, et al*. Association between sleep and blood pressure in midlife: the CARDIA sleep study. *Arch Intern Med* 2009;169:1055-61.

15 Grandner M, Mullington JM, Hashmi SD*, et al*. Sleep Duration and Hypertension: Analysis of > 700,000 Adults by Age and Sex. *J Clin Sleep Med* 2018;14:1031-39.

16 Fang J, Wheaton AG, Keenan NL*, et al*. Association of sleep duration and hypertension among US adults varies by age and sex. *Am J Hypertens* 2012;25:335-41.

17 Gottlieb DJ, Redline S, Nieto FJ*, et al*. Association of usual sleep duration with hypertension: the Sleep Heart Health Study. *Sleep* 2006;29:1009-14.

18 Magee CA, Kritharides L, Attia J*, et al*. Short and long sleep duration are associated with prevalent cardiovascular disease in Australian adults. *J Sleep Res* 2012;21:441-7.

For peer review only - http://bmjopen.bmj.com/site/about/guidelines.xhtml

19 Li M, Yan S, Jiang S, *et al*. Relationship between sleep duration and hypertension in northeast China: a cross-sectional study. *BMJ Open* 2019;9:e023916.

20 Chen Z, Lee L, Chen J, *et al*. Cohort profile: the Kadoorie Study of Chronic Disease in China (KSCDC). *Int J Epidemiol* 2005;34:1243-9.

21 Chen Z, Chen J Fau - Collins R, Collins R Fau - Guo Y, *et al*. China Kadoorie Biobank of 0.5 million people: survey methods, baseline characteristics and long-term follow-up.

22 Chen X, Du H, Zhang J, *et al*. Adiposity and blood pressure among 55 000 relatively lean rural adults in southwest of China. *J Hum Hypertens* 2015;29:522-9.

23 Lima-Costa MF, Peixoto SV, Rocha FL. Usual sleep duration is not associated with hypertension in Brazilian elderly: The Bambui Health Aging Study (BHAS). *Sleep Med* 2008;9:806-7.

24 Zhou BF. Predictive Values of Body Mass Index and Waist Circumference for Risk Factors of Certain Related Diseases in Chinese Adults - Study on Optimal Cut-off Points of Body Mass Index and Waist Circumference in Chinese Adults. *Biomedical & Environmental Sciences* 2002;11:S685-S93.

25 Tian X, Du H. Fruit consumption and physical activity in relation to all-cause and cardiovascular mortality among 70,000 Chinese adults with pre-existing vascular disease. 2017;12:e0173054.

26 Du H, Bennett D, Li L, *et al*. Physical activity and sedentary leisure time and their associations with BMI, waist circumference, and percentage body fat in 0.5 million adults: the China Kadoorie Biobank study. *Am J Clin Nutr* 2013;97:487-96.

27 Zheng B, Yu C, Lin L, *et al*. Associations of domain-specific physical activities with insomnia symptoms among 0.5 million Chinese adults. *J Sleep Res* 2017;26:330-37.

28 Durrleman S, Simon R. Flexible regression models with cubic splines. *Stat Med* 1989;8:551-61.

29 Smith, Patricia L. Splines As a Useful and Convenient Statistical Tool. *American Statistician* 1979;33:57-62.

30 Desquilbet L, Mariotti F. Dose-response analyses using restricted cubic spline functions in public health research. *Stat Med* 2010;29:1037-57.

31 Gangwisch JE, Feskanich D, Malaspina D, *et al*. Sleep duration and risk for hypertension in women: results from the nurses' health study. *Am J Hypertens* 2013;26:903-11.

32 Guo X, Zheng L, Wang J, *et al*. Epidemiological evidence for the link between sleep duration and high blood pressure: a systematic review and meta-analysis. *Sleep Med* 2013;14:324-32.

33 Wang Q, Xi B, Liu M, *et al*. Short sleep duration is associated with hypertension risk among adults: a systematic review and meta-analysis. *Hypertens Res* 2012;35:1012-8.

34 Makarem N, Shechter A, Carnethon MR, *et al*. Sleep Duration and Blood Pressure: Recent Advances and Future Directions. *Curr Hypertens Rep* 2019;21:33.

35 Wang H, Zee P, Reid K, *et al*. Gender-specific association of sleep duration with blood pressure in rural Chinese adults. *Sleep Med* 2011;12:693-9.

36 Kuciene R, Dulskiene V. Associations of short sleep duration with prehypertension and hypertension among Lithuanian children and adolescents: a cross-sectional study. *BMC Public Health* 2014;14:255.

37 Jarrin DC, Alvaro PK, Bouchard MA, *et al*. Insomnia and hypertension: A systematic review. *Sleep Med Rev* 2018;41:3-38.

38 Khazaie H, Negahban S, Ghadami MR, *et al*. Among middle-aged adults, snoring predicted hypertension independently of sleep apnoea. 2018;46:1187-96.

For peer review only - http://bmjopen.bmj.com/site/about/guidelines.xhtml

39 Lusardi P, Zoppi A, Preti P, *et al*. Effects of insufficient sleep on blood pressure in hypertensive patients: a 24-h study. *Am J Hypertens* 1999;12:63-8.

40 Spiegel K, Tasali E, Penev P, *et al*. Brief communication: Sleep curtailment in healthy young men is associated with decreased leptin levels, elevated ghrelin levels, and increased hunger and appetite. *Ann Intern Med* 2004;141:846-50.

41 Ayas NT, White DP, Al-Delaimy WK, *et al*. A prospective study of self-reported sleep duration and incident diabetes in women. *Diabetes Care* 2003;26:380-4.

42 Ayas NT, White DP, Manson JE, *et al*. A prospective study of sleep duration and coronary heart disease in women. *Arch Intern Med* 2003;163:205-9.

43 Suzuki E, Yorifuji T, Ueshima K, *et al*. Sleep duration, sleep quality and cardiovascular disease mortality among the elderly: a population-based cohort study. *Prev Med* 2009;49:135-41.

44 Cappuccio FP, Cooper D, D'Elia L, *et al*. Sleep duration predicts cardiovascular outcomes: a systematic review and meta-analysis of prospective studies. *Eur Heart J* 2011;32:1484-92.

45 Choi KM, Lee JS, Park HS, *et al*. Relationship between sleep duration and the metabolic syndrome: Korean National Health and Nutrition Survey 2001. *Int J Obes (Lond)* 2008;32:1091-7.

46 Lopez-Garcia E, Faubel R, Guallar-Castillon P, *et al*. Self-reported sleep duration and hypertension in older Spanish adults. *J Am Geriatr Soc* 2009;57:663-8.

47 Crowley K. Sleep and sleep disorders in older adults. *Neuropsychol Rev* 2011;21:41-53.

48 Fernandez-Mendoza J, Vgontzas AN, Liao D, *et al*. Insomnia with objective short sleep duration and incident hypertension: the Penn State Cohort. *Hypertension* 2012;60:929-35.

49 Budhiraja R, Roth T, Hudgel DW, *et al*. Prevalence and polysomnographic correlates of insomnia comorbid with medical disorders. *Sleep* 2011;34:859-67.

50 Floras JS. Hypertension and Sleep Apnea. *Can J Cardiol* 2015;31:889-97.

51 Kara B, Tenekeci EG. Sleep Quality and Associated Factors in Older Turkish Adults With Hypertension: A Pilot Study. *J Transcult Nurs* 2017;28:296-305.

52 Liu RQ, Qian Z, Trevathan E, *et al*. Poor sleep quality associated with high risk of hypertension and elevated blood pressure in China: results from a large population-based study. *Hypertens Res* 2016;39:54-9.

53 Fiorentini A, Valente R, Perciaccante A, *et al*. Sleep's quality disorders in patients with hypertension and type 2 diabetes mellitus. *Int J Cardiol* 2007;114:E50-2.

54 Du H, Li L, Bennett D, *et al*. Fresh Fruit Consumption and Major Cardiovascular Disease in China. *N Engl J Med* 2016;374:1332-43.

55 Du H, Li L, Bennett D, *et al*. Fresh fruit consumption and all-cause and cause-specific mortality: findings from the China Kadoorie Biobank. *Int J Epidemiol* 2017;46:1444-55.

Figure 1 Adjusted relationships between sleep duration and hypertension stratified by health-related factors. ORs in each group are presented as points with 95% CIs represented by extended lines. The horizontal reference dashed line represents an OR value of '1'. The full line represents the effects of different sleep durations on hypertension. A, 30-44 years old subgroup; B, 45-59 years old subgroup; C, 60-79 years old subgroup; D, Never or almost never tea consumers; E, Only occasionally tea consumers; F, Usually at least once a week tea consumers; G, Never regular alcohol drinker; H, Ex-regular alcohol drinker; I, Occasional or seasonal alcohol drinker; J, Usually at least once a week alcohol drinker; K, Never smoker; L, Ex-regular smoker; M, Occasional smoker; N, Smoked daily or almost every day; O, Non-regular fresh fruit consumers; P, regular fresh fruit consumers; Q,

For peer review only - http://bmjopen.bmj.com/site/about/guidelines.xhtml

insomnia subgroup; R, non-insomnia subgroup. Multivariable logistic model adjusted age, sex, education level, occupation, marital status, income, tea, smoking status, alcohol intake, MET, sedentary leisure time, fresh fruit consumption, insomnia, sleep snoring, BMI, WC, DM, stroke and CHD.

Figure 2 Adjusted relationships between sleep duration and hypertension by using restricted cubic splines regression in different gender and age subgroups, adjusted by age, sex, education level, occupation, marital status, income, tea consumption, smoking status, alcohol intake, MET, sedentary leisure time, fresh fruit consumption, insomnia, sleep snoring, BMI, WC, DM, stroke and CHD. ORs are presented as full line with 95% CIs represented by gray area. The horizontal reference dashed line represents an OR value of '1'. The vertical dashed line represents the sleep duration where OR equals 1.

For peer review only

For peer review only - http://bmjopen.bmj.com/site/about/guidelines.xhtml

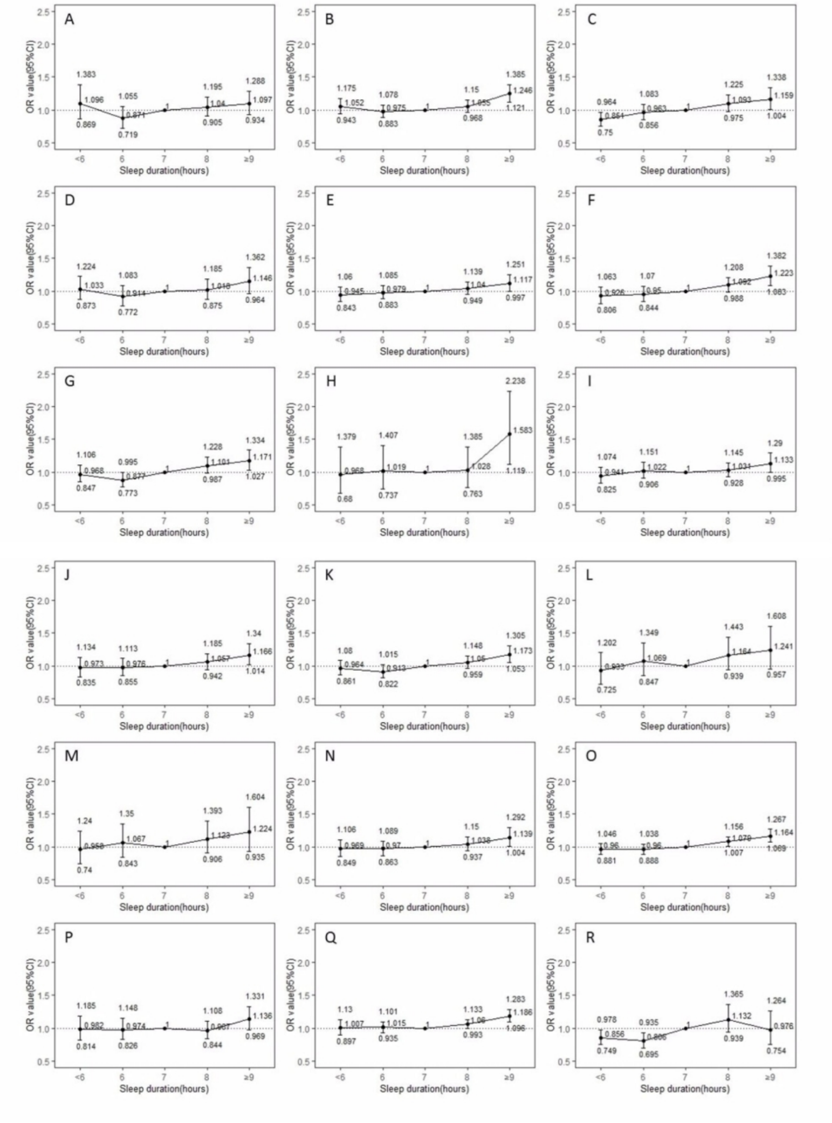

Figure 1 Adjusted relationships between sleep duration and hypertension stratified by health-related factors. ORs in each group are presented as points with 95% CIs represented by extended lines. The horizontal reference dashed line represents an OR value of '1'. The full line represents the effects of different sleep durations on hypertension. A, 30-44 years old subgroup; B, 45-59 years old subgroup; C, 60-79 years old subgroup; D, Never or almost never tea consumers; E, Only occasionally tea consumers; F, Usually at least once a week tea consumers; G, Never regular alcohol drinker; H, Ex-regular alcohol drinker; I, Occasional or seasonal alcohol drinker; J, Usually at least once a week alcohol drinker; K, Never smoker; L, Ex-regular smoker; M, Occasional smoker; N, Smoked daily or almost every day; O, Non-regular fresh fruit consumers; P, regular fresh fruit consumers; Q, insomnia subgroup; R, non-insomnia subgroup. Multivariable logistic model adjusted age, sex, education level, occupation, marital status, income, tea, smoking status, alcohol intake, MET, sedentary leisure time, fresh fruit consumption, insomnia, sleep snoring, BMI, WC, DM, stroke and CHD.

118x157mm (300 x 300 DPI)

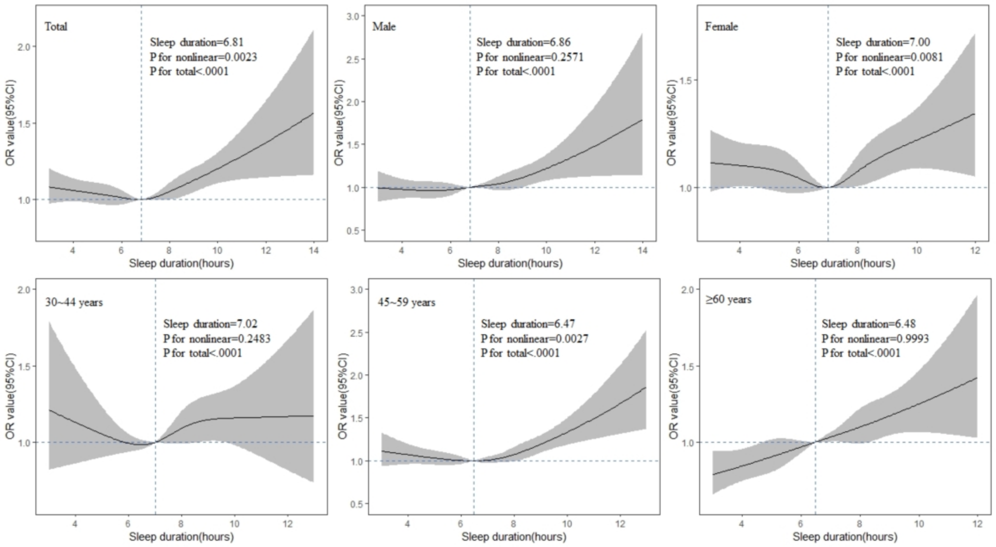

Figure 2 Adjusted relationships between sleep duration and hypertension by using restricted cubic splines regression in different gender and age subgroups, adjusted by age, sex, education level, occupation, marital status, income, tea consumption, smoking status, alcohol intake, MET, sedentary leisure time, fresh fruit consumption, insomnia, sleep snoring, BMI, WC, DM, stroke and CHD. ORs are presented as full line with 95% CIs represented by gray area. The horizontal reference dashed line represents an OR value of ‘1’. The vertical dashed line represents the sleep duration where OR equals 1.

472x265mm (300 x 300 DPI)

STROBE Statement—Checklist of items that should be included in reports of ***cross-sectional studies***

	Item No	Recommendation	Page No
**Title and abstract**	1	(*a*) Indicate the study's design with a commonly used term in the title or the abstract	1
		(*b*) Provide in the abstract an informative and balanced summary of what was done and what was found	2-3
**Introduction**			
Background/rationale	2	Explain the scientific background and rationale for the investigation being reported	4
Objectives	3	State specific objectives, including any prespecified hypotheses	4
**Methods**			
Study design	4	Present key elements of study design early in the paper	5
Setting	5	Describe the setting, locations, and relevant dates, including periods of recruitment, exposure, follow-up, and data collection	5
Participants	6	(*a*) Give the eligibility criteria, and the sources and methods of selection of participants	5
Variables	7	Clearly define all outcomes, exposures, predictors, potential confounders, and effect modifiers. Give diagnostic criteria, if applicable	5-7
Data sources/ measurement	8*	For each variable of interest, give sources of data and details of methods of assessment (measurement). Describe comparability of assessment methods if there is more than one group	5-7
Bias	9	Describe any efforts to address potential sources of bias	5-7
Study size	10	Explain how the study size was arrived at	5
Quantitative variables	11	Explain how quantitative variables were handled in the analyses. If applicable, describe which groupings were chosen and why	5-7
Statistical methods	12	(*a*) Describe all statistical methods, including those used to control for confounding	7-8
		(*b*) Describe any methods used to examine subgroups and interactions	7-8
		(*c*) Explain how missing data were addressed	None
		(*d*) If applicable, describe analytical methods taking account of sampling strategy	5-8
		(*e*) Describe any sensitivity analyses	5-8
**Results**			
Participants	13*	(a) Report numbers of individuals at each stage of study—eg numbers potentially eligible, examined for eligibility, confirmed eligible, included in the study, completing follow-up, and analysed	9-11
		(b) Give reasons for non-participation at each stage	None
		(c) Consider use of a flow diagram	None
Descriptive data	14*	(a) Give characteristics of study participants (eg demographic, clinical, social) and information on exposures and potential confounders	9-12
		(b) Indicate number of participants with missing data for each variable of interest	9-12
Outcome data	15*	Report numbers of outcome events or summary measures	12-22

For peer review only

For peer review only - http://bmjopen.bmj.com/site/about/guidelines.xhtml

Main results	16	(*a*) Give unadjusted estimates and, if applicable, confounder-adjusted estimates and their precision (eg, 95% confidence interval). Make clear which confounders were adjusted for and why they were included	12-22
		(*b*) Report category boundaries when continuous variables were categorized	12-22
		(*c*) If relevant, consider translating estimates of relative risk into absolute risk for a meaningful time period	12-22
Other analyses	17	Report other analyses done—eg analyses of subgroups and interactions, and sensitivity analyses	19-22
**Discussion**			
Key results	18	Summarise key results with reference to study objectives	23-27
Limitations	19	Discuss limitations of the study, taking into account sources of potential bias or imprecision. Discuss both direction and magnitude of any potential bias	26-27
Interpretation	20	Give a cautious overall interpretation of results considering objectives, limitations, multiplicity of analyses, results from similar studies, and other relevant evidence	23-27
Generalisability	21	Discuss the generalisability (external validity) of the study results	23-27
**Other information**			
Funding	22	Give the source of funding and the role of the funders for the present study and, if applicable, for the original study on which the present article is based	28

*Give information separately for exposed and unexposed groups.

Note: An Explanation and Elaboration article discusses each checklist item and gives methodological background and published examples of transparent reporting. The STROBE checklist is best used in conjunction with this article (freely available on the Web sites of PLoS Medicine at http://www.plosmedicine.org/, Annals of Internal Medicine at http://www.annals.org/, and Epidemiology at http://www.epidem.com/). Information on the STROBE Initiative is available at www.strobe-statement.org.

For peer review only

For peer review only - http://bmjopen.bmj.com/site/about/guidelines.xhtml
